# Efficacy of a low-dose candidate malaria vaccine, R21 in adjuvant Matrix-M, with seasonal administration to children in Burkina Faso: a randomised controlled trial

**DOI:** 10.1016/S0140-6736(21)00943-0

**Published:** 2021-05-15

**Authors:** Mehreen S Datoo, Magloire H Natama, Athanase Somé, Ousmane Traoré, Toussaint Rouamba, Duncan Bellamy, Prisca Yameogo, Daniel Valia, Moubarak Tegneri, Florence Ouedraogo, Rachidatou Soma, Seydou Sawadogo, Faizatou Sorgho, Karim Derra, Eli Rouamba, Benedict Orindi, Fernando Ramos Lopez, Amy Flaxman, Federica Cappuccini, Reshma Kailath, Sean Elias, Ekta Mukhopadhyay, Andres Noe, Matthew Cairns, Alison Lawrie, Rachel Roberts, Innocent Valéa, Hermann Sorgho, Nicola Williams, Gregory Glenn, Louis Fries, Jenny Reimer, Katie J Ewer, Umesh Shaligram, Adrian V S Hill, Halidou Tinto

**Affiliations:** a Centre for Clinical Vaccinology and Tropical Medicine, The Jenner Institute, University of Oxford and the NIHR Oxford Biomedical Research Centre, Oxford, UK; b Unité de Recherche Clinique de Nanoro, Institut de Recherche en Sciences de la Santé, Nanoro, Burkina Faso; c The Jenner Institute Laboratories, University of Oxford, UK; d Department of Primary Care, University of Oxford, UK; e KEMRI-Wellcome Trust Research Programme, Kilifi, Kenya; f London School of Hygiene & Tropical Medicine, London, UK; g Novavax, Gaithersburg, MD, USA; h Novavax, Uppsala, Sweden; i Serum Institute of India, Pune, India

## Abstract

**Background:**

Stalled progress in controlling *Plasmodium falciparum* malaria highlights the need for an effective and deployable vaccine. RTS,S/AS01, the most effective malaria vaccine candidate to date, demonstrated 56% efficacy over 12 months in African children. We therefore assessed a new candidate vaccine for safety and efficacy.

**Methods:**

In this double-blind, randomised, controlled, phase 2b trial, the low-dose circumsporozoite protein-based vaccine R21, with two different doses of adjuvant Matrix-M (MM), was given to children aged 5–17 months in Nanoro, Burkina Faso—a highly seasonal malaria transmission setting. Three vaccinations were administered at 4-week intervals before the malaria season, with a fourth dose 1 year later. All vaccines were administered intramuscularly into the thigh. Group 1 received 5 μg R21 plus 25 μg MM, group 2 received 5 μg R21 plus 50 μg MM, and group 3, the control group, received rabies vaccinations. Children were randomly assigned (1:1:1) to groups 1–3. An independent statistician generated a random allocation list, using block randomisation with variable block sizes, which was used to assign participants. Participants, their families, and the local study team were all masked to group allocation. Only the pharmacists preparing the vaccine were unmasked to group allocation. Vaccine safety, immunogenicity, and efficacy were evaluated over 1 year. The primary objective assessed protective efficacy of R21 plus MM (R21/MM) from 14 days after the third vaccination to 6 months. Primary analyses of vaccine efficacy were based on a modified intention-to-treat population, which included all participants who received three vaccinations, allowing for inclusion of participants who received the wrong vaccine at any timepoint. This trial is registered with ClinicalTrials.gov, NCT03896724.

**Findings:**

From May 7 to June 13, 2019, 498 children aged 5–17 months were screened, and 48 were excluded. 450 children were enrolled and received at least one vaccination. 150 children were allocated to group 1, 150 children were allocated to group 2, and 150 children were allocated to group 3. The final vaccination of the primary series was administered on Aug 7, 2019. R21/MM had a favourable safety profile and was well tolerated. The majority of adverse events were mild, with the most common event being fever. None of the seven serious adverse events were attributed to the vaccine. At the 6-month primary efficacy analysis, 43 (29%) of 146 participants in group 1, 38 (26%) of 146 participants in group 2, and 105 (71%) of 147 participants in group 3 developed clinical malaria. Vaccine efficacy was 74% (95% CI 63–82) in group 1 and 77% (67–84) in group 2 at 6 months. At 1 year, vaccine efficacy remained high, at 77% (67–84) in group 1. Participants vaccinated with R21/MM showed high titres of malaria-specific anti-Asn-Ala-Asn-Pro (NANP) antibodies 28 days after the third vaccination, which were almost doubled with the higher adjuvant dose. Titres waned but were boosted to levels similar to peak titres after the primary series of vaccinations after a fourth dose administered 1 year later.

**Interpretation:**

R21/MM appears safe and very immunogenic in African children, and shows promising high-level efficacy.

**Funding:**

The European & Developing Countries Clinical Trials Partnership, Wellcome Trust, and National Institute for Health Research Oxford Biomedical Research Centre.

## Introduction

Malaria remains one of the leading causes of morbidity and mortality worldwide. *Plasmodium falciparum* is a complex pathogen with numerous immune evasion mechanisms. Development of an efficacious vaccine against this parasite has remained elusive for many decades. The leading malaria vaccine candidate, RTS,S/AS01, induces partial efficacy through induction of antibodies against the central repeat (Asn-Ala-Asn-Pro [NANP]) of the circumsporozoite protein (CSP).^1^ Efficacy was assessed in a phase 3 study of 15 460 children and infants living in seven sub-Saharan African countries between 2009 and 2013.^2^, ^3^ Overall vaccine efficacy for children aged 5–17 months, with a median follow-up of 48 months, was 36% for children administered RTS,S/AS01 at months 0, 1, 2, and 20, and 28% for children given the vaccine at months 0, 1, and 2. For infants aged 6–12 weeks, efficacy was 26% for children given the vaccine at months 0, 1, 2, and 20, and 18% for children given the vaccine at months 0, 1, and 2.^2^ These studies showed modest efficacy, leading to a positive scientific opinion by the European Medicines Agency; however, possible safety signals of increased incidence of meningitis, cerebral malaria cases,^2^, ^4^, ^5^ and increased female mortality in malaria vaccine groups were also observed.^6^, ^7^ The question of feasibility of a four-dose schedule requiring new contacts also arose. Therefore, RTS,S/AS01 has not yet been prequalified for use by WHO, but instead a malaria vaccine implementation programme was launched in three countries over the course of 2019.^8^

**Research in context**

**Evidence before this study**

There are currently no licensed vaccines to protect against *Plasmodium falciparum* malaria. We searched PubMed from database inception to March 23, 2021, for published research articles using the terms “malaria vaccine”, “clinical trial”, “phase III”, AND “efficacy”. No language restrictions were applied. The search identified one published large phase 3 clinical trial describing a trial of the pre-erythrocytic malaria vaccine candidate, RTS,S/AS01 (Mosquirix), done at 11 sites in seven countries across sub-Saharan Africa. This vaccine candidate has now progressed to pilot implementation trials after showing efficacy of 36% after four doses, over a median of 48 months follow-up. Efficacy of 56% in children aged 5–17 months was observed over the first year.

Due to the high burden and wide geographical distribution of *P falciparum*, in the most recent update to the Malaria Vaccine Technology Roadmap, WHO called for the development of malaria vaccine candidates with a protective efficacy of at least 75% against clinical malaria by 2030, to address this unmet priority public health goal.

**Added value of this study**

This study reports vaccine efficacy of a novel pre-erythrocytic candidate malaria vaccine in a phase 2 trial in children living in a malaria endemic area with high transmission in Burkina Faso. This new vaccine, R21 adjuvanted with 50 μg Matrix-M (R21/MM), administered before the malaria season, demonstrates high-level efficacy, reaching the WHO-specified efficacy goal of at least 75% in the target population of African children over 1 year. Furthermore, R21/MM demonstrates a favourable safety profile. It is well tolerated with the majority of local and systemic adverse events graded mild, and no serious adverse events related to vaccination in the trial. Importantly, although this vaccine immunogen is similar to RTS,S, it does not have the excess HBsAg found in RTS,S and provides a higher density of circumsporozoite protein epitopes on the particle surface, resulting in high levels of malaria-specific anti-Asn-Ala-Asn-Pro (NANP) antibodies. These antibodies were effectively boosted 1 year later to levels similar to those after the primary series of vaccinations.

This phase 2 trial is currently continuing for a second malaria season, after the booster vaccine, to determine whether high vaccine efficacy can be maintained.

**Implications of all the available evidence**

These initial findings with the new R21/MM vaccine candidate appear to improve on the efficacy in children of all other malaria vaccines. These data support the further evaluation of this promising malaria vaccine candidate in a phase 3 trial that will embrace different malaria transmission settings, the coadministration of seasonal malaria chemoprevention, and encompass a wider age range with a high incidence of malaria. An important additional advantage of the new R21/MM malaria vaccine candidate is its potential for large-scale manufacturing and low-cost supply, to support global efforts to better control, sustainably eliminate, and finally eradicate malaria.

There remains an urgent need to identify and develop improved vaccine candidates that could achieve the WHO goal of 75% efficacy against clinical malaria by 2030.^9^ R21 is a novel pre-erythrocytic candidate malaria vaccine. R21 and RTS,S both include HBsAg fused to the C-terminus and central repeats of the CSP, which self-assemble into virus-like particles in yeast. R21 lacks the excess HBsAg found in RTS,S. R21 comprises only fusion protein moieties, in contrast to RTS,S, which comprises 20% with the remaining 80% being HBsAg monomers expressed alone, thereby likely diminishing CSP coverage of the virus-like particle surface.^10^, ^11^

Following preclinical studies of R21 plus multiple adjuvants, Matrix-M (R21/MM) was selected for clinical development based on high immunogenicity.^10^ It is a saponin-based adjuvant that stimulates both humoral and cellular immune responses to vaccines.^10^, ^12^ In phase 1/2a clinical trials, R21/MM showed a good safety profile and strong antibody responses to the CSP central repeat, NANP, using a dose of 5 μg R21. Importantly, sterile efficacy rates of 63–78% were observed during controlled human malaria infection trials after three doses of 10 μg R21/MM, administered intramuscularly 4 weeks apart.^13^, ^14^

Following an age de-escalation trial of R21/MM in Kenyan adults, children, and infants,^15^ which has shown a well tolerated safety profile and potent immunogenicity, we initiated a phase 1/2b safety, immunogenicity, and efficacy trial of this novel pre-erythrocytic malaria vaccine candidate in children aged 5–17 months in Nanoro, Burkina Faso. To ensure antibody responses were highest during the seasonal peak of malaria transmission, resulting in potentially increased vaccine efficacy, we administered three doses in the primary vaccination series largely before the malaria season.^16^

## Methods

### Study design and participants

This was a phase 2b, randomised, controlled, double-blind trial done at the Institut de Recherche en Sciences de la Santé, Nanoro, Burkina Faso. Participants aged 5–17 months were recruited from the Health and Demographic Surveillance System catchment area of Nanoro, which covers 24 villages, with an approximate population of 65 000 inhabitants. Nanoro is an area of high malaria transmission, with transmission occurring throughout the year, but with a marked peak during the rainy season (June to November).

Eligible participants were recruited into three groups. Group 1 received 5 μg R21/25 μg MM, group 2 received 5 μg R21/50 μg MM, and group 3, the control group, received rabies vaccinations. Doses were administered before the seasonal peak of malaria transmission starting in July. Safety, immunogenicity, and vaccine efficacy are being assessed over 24 months, with the primary efficacy endpoint after 6 months, after the primary series of vaccinations (three doses). All participants also received a booster vaccination approximately 12 months after their third vaccination, before the start of the next malaria season. Field workers collected data on indoor residual spraying of households, insecticide treated net use (and if the nets were adequate, according to if holes were present), number of doses of seasonal malaria chemoprevention taken by the participant per month, and number of months seasonal malaria chemoprevention was taken during the malaria season.

After community sensitisation, a list of eligible children was drawn from the Health and Demographic Surveillance System database, and parents or legally authorised guardians who expressed interest were invited to screening visits. During recruitment, parents or legally authorised guardians of participants provided written or thumb-printed consent, which was verbally checked at each study visit. Inclusion criteria specified that participants should be aged 5–17 months at enrolment, parents should provide written informed consent, and aim to be living in the study area for the trial duration. Exclusion criteria included significant comorbidities and participation in other malaria intervention studies and clinical trials. Further details are given in the protocol (appendix pp 13–91).

The trial was approved by the Comité d'Ethique pour la Recherche en Santé, Burkina Faso (reference number 2019-01-012), and the national regulatory authority, Agence National de Régulation Pharmaceutique, Burkina Faso (reference number 5005420193EC0000). Ethical approval was also granted in the UK by the Oxford Tropical Research Ethics Committee (reference number 19-19).

### Randomisation and masking

Children aged 5–17 months were randomly assigned (1:1:1) to groups 1–3. An independent statistician generated a random allocation list, using block randomisation with variable block sizes. A person independent of the trial prepared and sealed the envelopes using this list, which was then given to the pharmacist to assign to participants. Both malaria and control vaccines were prepared by the pharmacist using the same type of syringe, and the contents of the syringe were covered with an opaque label. The trial was double-blinded; participants, their families, and the local study team were all masked to group allocation. Only the pharmacists preparing the vaccine were unmasked to group allocation.

### Procedures

R21 was produced by expressing recombinant HBsAg virus-like particles in *Hansenula polymorpha*, comprising the central repeat and the C-terminus of the CSP fused to the N-terminal end of HBsAg^10^ and manufactured by the Serum Institute of India (Pune, India). R21 was mixed immediately before administration with MM, a saponin-based vaccine adjuvant produced by Novavax (Uppsala, Sweden). A rabies vaccine (Rabivax-S), manufactured by the Serum Institute of India, was the control vaccine. All vaccines were administered intramuscularly into the thigh.

On the day of vaccination, participants were tested for malaria if they had a fever of 37·5°C or higher. If their blood film was positive for *Plasmodium* spp, they were treated for malaria in accordance with national guidelines before having a vaccination.

After each vaccination, local and systemic solicited adverse events were collected for 7 days. Intensity of symptoms was evaluated following standardised methods. Unsolicited adverse events were collected for 28 days after vaccinations, and safety laboratory values were measured at 28 days after the first and third vaccinations to look for deviations from baseline. Serious adverse events are being recorded for the duration of the study. Clinical judgment by study clinicians was used to assess causality of adverse events and relationship to vaccine. All adverse events were followed up until resolution.

Data safety monitoring board reviews were held after the vaccination of the first 30 participants, and after completion of the primary series of three vaccinations.

Parents of participants were advised to attend the community health facility if their child had any illness, or a temperature of 37·5°C or higher or history of fever within the last 24 h, or both, for review and assessment for malaria. After the third vaccination, participants were visited by field workers every 30 days until 6 months after the third vaccination, when, if they had a temperature of 37·5°C or higher or history of fever within the last 24 h, or both, blood sampling was done for blood film microscopy to detect *Plasmodium* spp. Two independent microscopists, who were masked to the vaccination status of all participants, analysed each blood film, with a third microscopist adjudicating in cases of discrepancy.

Anti-NANP antibodies were measured by ELISA before first vaccination, as previously described,^17^, ^18^ 28 days after first vaccination; 28 days, 6 months, and 1 year after the third vaccination; and 28 days after the booster dose administered 1 year later.

### Outcomes

The primary objective assessed protective efficacy of R21/MM against clinical malaria from 14 days after the third vaccination to 6 months. The primary case definition of clinical malaria was presence of an axillary temperature of 37·5°C or higher and *P falciparum* asexual parasite density of more than 5000 parasites per μL. The secondary case definition was presence of an axillary temperature of 37·5°C or higher or history of fever during the last 24 h, or both, and *P falciparum* parasite density of more than 0 parasites per μL. The secondary objective assessed protective efficacy of R21/MM from 14 days after the third vaccination to 12 months. In addition, cross-sectional asymptomatic *P falciparum* infection was analysed at months 6 and 12, defined as the presence of axillary temperature of less than 37·5°C, absence of history of fever within the last 24 h, and *P falciparum* parasite density of more than 0 parasites per μL. Safety, reactogenicity, and humoral immunogenicity of R21/MM were evaluated.

### Statistical analysis

The study was powered to provide an initial point estimate of the efficacy of the malaria vaccine in either group 1 or 2, assuming that the vaccine efficacy over 6 months was greater than 50%. Due to an unexpectedly high participant retention rate, we had power to detect efficacy greater than 37%.

Cox regression models were used to analyse first episodes of clinical malaria from 14 days after the third vaccination to 6 months and 1 year. For participants without an episode of clinical malaria, their time was censored at the date of their withdrawal or the date of their 6-month or 12-month blood sampling. The primary comparisons were prespecified as being between groups 1 and 3 and groups 2 and 3, with comparison of groups 1 and 2 to 3 as a supplementary analysis. A secondary analysis adjusted for confounding factors of sex, age at randomisation (5–9 months, 10–12 months, or >12 months) and bednet use (adequate or not). Vaccine efficacy was calculated as 1 minus the hazard ratio (HR).

Primary analyses of vaccine efficacy were based on a modified intention-to-treat population, which included all participants who received three vaccinations, allowing for inclusion of participants who received the wrong vaccine at any timepoint. Because all vaccines were administered correctly, this is equivalent to a per-protocol analysis.

The secondary outcomes of asymptomatic malaria infection at months 6 and 12 were analysed using a log binomial model, including randomised group as a covariate. Relative risks and 95% CIs were reported for comparisons of groups 1 and 3, and groups 2 and 3. This analysis was also done with adjustment for the confounding factors previously described.

To search for an immunological correlate of protection, we divided participants by tertile on their antibody response 4 weeks after the third dose and searched for differences in risk of clinical malaria.^2^

To facilitate masking, analyses were done by statisticians external to the investigator teams.

All statistical analyses were done using Stata, version 16.1.

This study is registered with ClinicalTrials.gov, NCT03896724.

### Role of the funding source

The funder of the study had no role in study design, data collection, data analysis, data interpretation, or writing of the report. All authors had full access to all the data in the study and had final responsibility for the decision to submit for publication.

## Results

**Figure 1:**
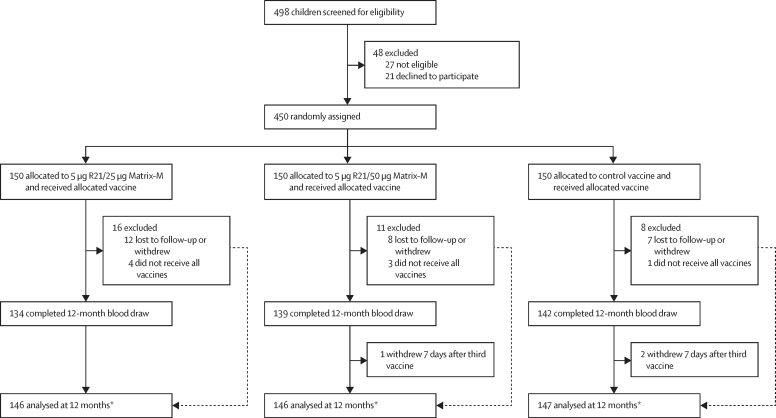
Trial profile The main reason for withdrawal or not completing vaccination regimen was relocation outside of the study area. The parent of one participant withdrew consent after the first vaccination and two participants died during the course of the study, unrelated to vaccination. *All participants who received the third vaccination were analysed for the primary outcome, because participants with no event were censored at date of 12-month blood draw or date of withdrawal, except for three participants who withdrew within 14 days of third vaccination.

From May 7 to June 13, 2019, 498 children aged 5–17 months were screened (figure 1). 48 children were excluded, leaving 450 children who were enrolled and received at least one vaccination. 150 children were allocated to 5 μg R21 plus 25 μg MM (group 1), 150 children were allocated to 5 μg R21 plus 50 μg MM (group 2), and 150 children were allocated to the control vaccine (group 3). The final vaccination of the primary series was administered on Aug 7, 2019. Baseline demographic characteristics were similar across the groups and the combined mean age of children completing vaccinations was 11·6 months (SD 3·8), with 220 male participants and 222 female participants (appendix p 1). Eight of the 450 participants enrolled withdrew before the third vaccination and three at 7 days after the third vaccination. 383 (87%) of 442 participants adequately used insecticide treated nets before the malaria season. Indoor residual spraying was done in 65 (15%) of 441 households, and 300 (68%) of 442 participants had at least one round (ie, three consecutive doses per day in 1 month; appendix p 2) of seasonal malaria chemoprevention.

**Figure 2:**
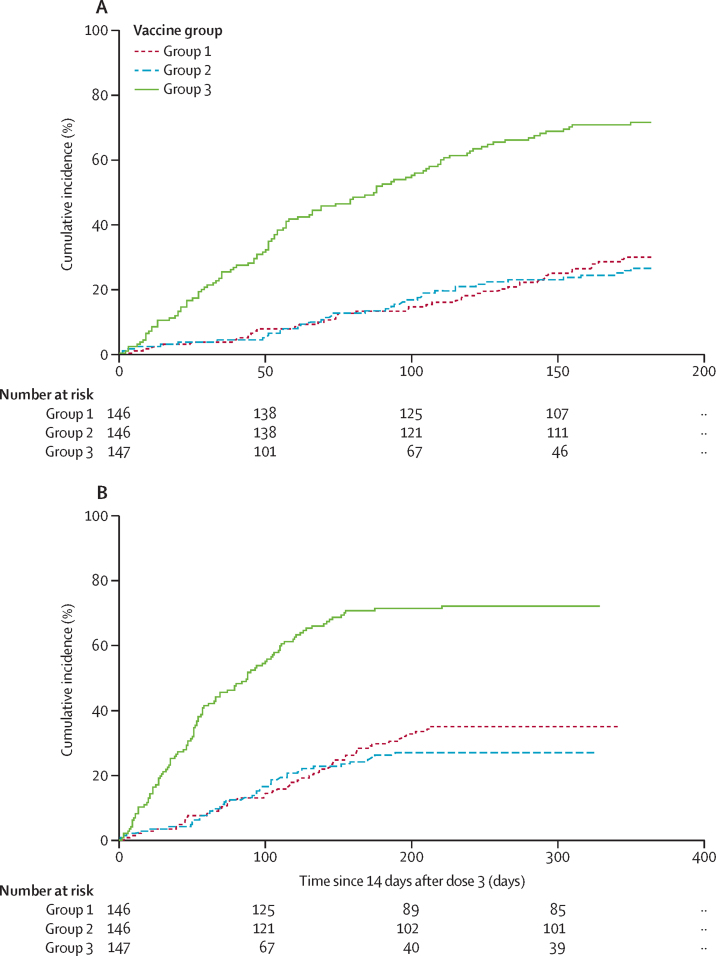
Kaplan-Meier estimates of the time to first episode of clinical malaria The primary analysis was based on a modified intention-to-treat population. Group 1 received 5 μg R21/25 μg MM, group 2 received 5 μg R21/50 μg MM, and group 3, the control group, received rabies vaccinations (Rabivax-S). (A) Data beginning from 14 days to 6 months after third vaccination. (B) Data beginning from 14 days to 12 months after third vaccination. MM=Matrix-M.

186 participants had clinical malaria according to the primary case definition when assessing the primary objective of efficacy against clinical malaria of R21/MM from 14 days after the third vaccination to 6 months. These cases of clinical malaria occurred in 43 (29%) of 146 participants in group 1, 38 (26%) of 146 participants in group 2, and 105 (71%) of 147 participants in group 3. A Cox regression model comparing group 1 with group 3 resulted in vaccine efficacy of 74% (95% CI 63–82; p<0·0001). Comparing group 2 with group 3 resulted in 77% efficacy (67–84; p<0·0001; figure 2).

Efficacy was further assessed at 12 months (range 329–369 days) after the third vaccination. 195 participants had clinical malaria—an increase of nine participants from the primary 6-month analysis (the period of low malaria transmission). These cases occurred in 50 (34%) of 146 participants in group 1, 39 (27%) of 146 participants in group 2, and 106 (72%) of 147 participants in group 3. Cox regression showed vaccine efficacy of 71% (95% CI 59–79; p<0·0001) for group 1, and 77% (67–84; p<0·0001) for group 2 (figure 2). No significant difference in disease incidence was found between groups 1 and 2 at either 6 months or 12 months. Calculation of the numbers of cases that would be averted^19^ by the vaccination regimens, based on numbers of all malaria episodes and case incidence rates over 12 months (appendix p 2), indicated a rate reduction of 1393 cases (95% CI 1043–1744) per 1000 children-years in group 1, and 1523 cases (1172–1875) per 1000 children-years in group 2 (appendix p 2).

224 participants had a first episode of clinical malaria by 12 months according to the secondary case definition, including clinical cases with parasitaemia of more than 0 parasites per μL. A Cox regression model comparing group 1 with group 3 showed vaccine efficacy of 70% (95% CI 60–78; p<0·0001), and 80% (72–86; p<0·0001) when comparing group 2 with group 3 (table 1).

**Table 1:** Time to first episode of malaria meeting case definitions of clinical malaria episode, from 14 days to 6 and 12 months following third vaccination

	**Timepoint (months)**	**Number with at least one episode of clinical malaria**	**Unadjusted efficacy**	Adjusted efficacy*
**Group 1**				
Primary (>5000 parasites per μL)	6	43/146 (30%)	74% (63–82)	74% (63–82)
Primary (>5000 parasites per μL)	12	50/146 (34%)	71% (59–79)	71% (59–79)
Secondary (>0 parasites per μL)	6	53/146 (36%)	73% (63–81)	74% (64–81)
Secondary (>0 parasites per μL)	12	61/145 (42%)	70% (60–78)	71% (60–79)
**Group 2**				
Primary (>5000 parasites per μL)	6	38/146 (26%)	77% (67–84)	76% (65–84)
Primary (>5000 parasites per μL)	12	39/146 (27%)	77% (67–84)	76% (65–84)
Secondary (>0 parasites per μL)	6	43/146 (30%)	79% (70–85)	78% (69–85)
Secondary (>0 parasites per μL)	12	43/146 (30%)	80% (72–86)	80% (71–86)
**Groups 1 and 2 combined**				
Primary (>5000 parasites per μL)	6	81/292 (28%)	76% (67–82)	75% (67–82)
Primary (>5000 parasites per μL)	12	89/292 (31%)	74% (65–80)	73% (65–80)
Secondary (>0 parasites per μL)	6	96/292 (33%)	76% (69–82)	76% (69–82)
Secondary (>0 parasites per μL)	12	104/291 (36%)	75% (68–81)	75% (67–81)
**Group 3 (control group)**				
Primary (>5000 parasites per μL)	6	105/147 (71%)	NA	NA
Primary (>5000 parasites per μL)	12	106/147 (72%)	NA	NA
Secondary (>0 parasites per μL)	6	118/147 (80%)	NA	NA
Secondary (>0 parasites per μL)	12	120/147 (82%)	NA	NA

Data are n/N (%) or % (95% CI), unless stated otherwise. Group 1 received 5 μg R21/25 μg Matrix-M, group 2 received 5 μg R21/50 μg Matrix-M, and group 3 received Rabivax-S. Primary analysis was based on a modified intention-to-treat population. Primary case definition of clinical malaria is presence of axillary temperature of 37·5°C or higher and *Plasmodium falciparum* parasite density of more than 5000 asexual forms per μL. Secondary case definition of clinical malaria is presence of axillary temperature of 37·5°C or higher or history of fever within the last 24 h, or both, and *P falciparum* parasite density of more than 0. The Cox proportional hazards model was used to calculate hazard ratio. Vaccine efficacy was calculated by 1 minus the hazard ratio and expressed as a percentage. NA=not applicable.

* Cox proportional hazards model, adjusted for sex, age category (5–9 months, 10–12 months, and >12 months) and adequate insecticide treated net use. All p values comparing vaccination groups to the control group for efficacy were less than 0·0001.

Secondary analyses of vaccine efficacy, according to the primary case definition, were done between 14 days after the third vaccination and 6 months or 12 months, adjusting for potentially confounding factors of sex, age at randomisation, and adequate bednet use. Using a Cox regression model, comparing group 1 with group 3 showed a vaccine efficacy of 75% (95% CI 67–81; p<0·0001) and comparing group 2 with group 3 showed a vaccine efficacy of 77% (65–80; p<0·0001) at 12 months (table 1). Further adjustment for use of seasonal malaria chemoprevention showed no change to the vaccine efficacy estimates.

Cross-sectional blood films were done after 6 months and 12 months of follow-up. At 6 months, 28 (19%) of 147 participants in group 3 had asymptomatic parasitaemia, with fewer participants in groups 1 (12 [9%] of 140; p=0·01), and 2 (13 [9%] of 145; p=0·01) with asymptomatic parasitaemia. At 12 months, this number was reduced to six (4%) of 142 participants in group 3, three (2%) of 132 participants (p=0·37) in group 1; and two (1%) of 141 participants (p=0·18) in group 2 (appendix p 4).

Seven serious adverse events were reported in participants (appendix p 3) and all were deemed unrelated to vaccination. One serious adverse event was a participant who presented 17 days after their first R21/MM vaccination with fever, a convulsion, and generally unwell. They were severely anaemic with a blood film positive for *P falciparum*. They were diagnosed and treated for severe malaria, and transferred to the referral hospital but died shortly thereafter.

Local adverse events of redness, swelling, and pain were reported in a small proportion of participants. 25 (2%) episodes of pain were noted after 1159 R21/MM vaccinations (table 2; appendix pp 5–6). Fever, loss of appetite, irritability, and drowsiness were the systemic adverse events collected for 7 days after each vaccination. Fever was the most common adverse event for all groups, occurring in 14 (9%) of 150 participants in group 1, 28 (19%) of 150 participants in group 2, and 13 (9%) of 150 participants in group 3 after the first vaccination. Fever occurred in 18 (12%) of 149 participants in group 1, 44 (30%) of 147 participants in group 2, and seven (5%) of 149 participants in group 3 after the second vaccination; and in 18 (12%) of 146 participants in group 1, 29 (20%) of 147 participants in group 2, and 16 (11%) of 149 participants in group 3 after the third vaccination (table 2; appendix p 5). After the booster dose, rates of fever were similar, occurring in 19 (14%) of 132 participants in group 1, 34 (25%) of 138 participants in group 2, and eight (6%) of 140 participants in group 3 (table 2; appendix p 6). Only two participants had grade 3 fever (>39·0°C), and these events were after the third vaccination in groups 1 and 2. Overall, there were significantly more fevers in group 2 (odds ratio 2·214 [95% CI 1·614–3·026]; p<0·0001) than group 1 (table 2; appendix pp 7–8). No participants experienced febrile convulsions.

**Table 2:** Incidence of adverse events across all the groups by number of doses

		**Group 1**	**Group 2**	**Group 3**
**Local adverse event**				
Pain				
	1	6 (4%)	9 (6%)	3 (2%)
	2	3 (2%)	3 (2%)	0 (0%)
	3	0 (0%)	0 (0%)	0 (0%)
	4	4 (3%)	0 (0%)	0 (0%)
Redness				
	1	2 (1%)	1 (1%)	0 (0%)
	2	10 (7%)	14 (10%)	2 (1%)
	3	2 (2%)	2 (1%)	1 (1%)
	4	3 (2%)	0 (0%)	0 (0%)
Swelling				
	1	3 (2%)	5 (3%)	2 (1%)
	2	14 (9%)	23 (16%)	10 (7%)
	3	8 (6%)	11 (8%)	4 (3%)
	4	3 (2%)	0 (0%)	0 (0%)
**Systemic adverse event**				
Fever				
	1	14 (9%)	28 (19%)	13 (9%)
	2	18 (12%)	44 (30%)	7 (5%)
	3	18 (12%)	29 (20%)	16 (11%)
	4	19 (14%)	34 (25%)	8 (6%)
Irritability				
	1	2 (1%)	5 (3%)	0 (0%)
	2	0 (0%)	4 (3%)	0 (0%)
	3	0 (0%)	0 (0%)	1 (1%)
	4	1 (1%)	1 (1%)	1 (1%)
Drowsiness				
	1	0 (0%)	3 (2%)	1 (1%)
	2	2 (1%)	6 (4%)	2 (1%)
	3	0 (0%)	0 (0%)	0 (0%)
	4	0 (0%)	2 (1%)	0 (0%)
Loss of appetite				
	1	1 (1%)	2 (1%)	1 (1%)
	2	0 (0%)	3 (2%)	1 (1%)
	3	0 (0%)	0 (0%)	0 (0%)
	4	0 (0%)	1 (1%)	1 (1%)

Data are n or n (%). Group 1 received 5 μg R21/25 μg Matrix-M, group 2 received 5 μg R21/50 μg Matrix-M, and group 3, the control group, received Rabivax-S. All solicited local and systemic adverse events were collected for 7 days after each vaccination. 150 participants in each group received the first dose of the vaccination. 149 participants in group 1, 147 participants in group 2 and 149 participants in group 3 received a second dose. 146 participants in group 1, 147 participants in group 2, and 149 participants in group 3 received a third dose. 132 participants in group 1, 138 participants in group 2, and 140 participants in group 3 received a fourth dose. Fever was defined as a temperature of 37·5°C or above. One participant in group 1 and one participant in group 2 had a severe fever (>39°C) after the third dose; all other adverse events were graded as mild or moderate. The grading of adverse events is given in the appendix (pp 13–91).

Unsolicited adverse events were collected for 28 days after each vaccination and were categorised according to the Medical Dictionary for Regulatory Activities preferred terms. 811 terms were assigned after three vaccinations, and there were no significant differences in number of events per group (appendix pp 7–9). Laboratory safety tests did not reveal any significant difference in the frequency of out-of-range values between the three treatment groups. Anaemia and leukocytosis were noted across the groups, but these events were assessed by masked study clinicians as not related to vaccinations. The only episodes of severe anaemia that occurred were during acute illness reported as serious adverse events and none were deemed related to vaccination (appendix p 3).

**Figure 3:**
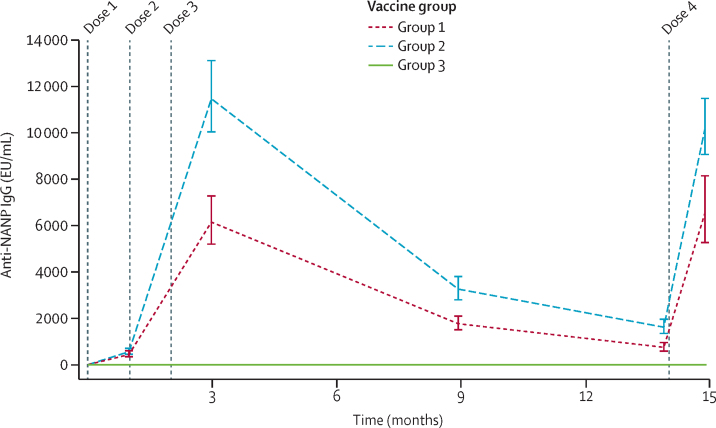
Antibody responses to R21/MM (A) Geometric mean antibody titres (95% CI). Anti-NANP antibodies were measured by ELISA at baseline; 28 days after first vaccination; 28 days, 6 months, and 1 year after the third vaccination; and 28 days after the booster (fourth) dose administered 1 year after the third dose. Group 1 received 5 μg R21/25 μg MM, group 2 received 5 μg R21/50 μg MM, and group 3, the control group, received Rabivax-S. MM=Matrix-M. NANP=Asn-Ala-Asn-Pro.

At baseline, no participant had detectable NANP IgG antibody levels. In group 1, titres reached a geometric mean of 6133 (95% CI 5161–7289) at 28 days after the third vaccination—about three times the level observed in vaccinated adults in the UK.^17^ In group 2, who received the higher dose of adjuvant, the level was significantly higher at 11 438 (9985–13 102; p<0·0001). These titres dropped over the following 12 months, but in both groups 1 and 2, 28 days after the fourth vaccination, antibodies were boosted to levels similar to those after the third vaccination (figure 3).

The antibody levels 28 days after the third vaccination were assessed for correlation with vaccine efficacy. After dividing antibody response levels to NANP of the combined group 1 and 2 participants into tertiles—an approach used successfully to identify an immune correlate of vaccine efficacy for RTS,S/AS01^2^—there was a significantly reduced risk of malaria over 6 months for participants in the upper tertile compared with participants in the lower tertile (HR 0·34, 95% CI 0·19–0·63; p<0·0001), and for participants in the upper tertile compared with participants in the middle tertile (0·46, 0·25–0·86; p<0·015).

## Discussion

We report malaria vaccine efficacy that reaches the WHO-specified efficacy goal of 75% or more^20^ in the target population of African children, over 12 months of follow-up. Efficacy of 77% was observed in the children who received R21 plus the higher dose of MM adjuvant, and it was associated with an 86% increase in antibody titres to the CSP repeat, NANP, compared with the lower adjuvant dose after the third vaccination (figure 3). This efficacy has been studied in an area of highly seasonal malaria transmission.

R21/MM vaccinations were given before and during the start of the malaria season. It remains unclear whether, and by how much, vaccine efficacy might be increased by this approach in seasonal areas, but this is being studied with RTS,S/AS01 in Mali and Burkina Faso (NCT03143218).^21^ It is possible that vaccine efficacy might be further improved if administered earlier, so the primary series of vaccinations (all three doses) are completed some weeks before the season.

Despite the policy recommendation of four monthly courses of seasonal malaria chemoprevention in Burkina Faso,^22^ we found that most participants did not receive all doses from national programme administration during the malaria season. This study did not aim to ensure seasonal malaria chemoprevention delivery as per the national policy recommendation, but rather to document actual uptake and allow vaccine efficacy to be measured in this real-world context. A 2020 study concluded that malaria burden was still high in Burkina Faso, despite the introduction of seasonal malaria chemoprevention and a fifth monthly course would be of value.^23^ We are unaware of evidence that different levels of seasonal malaria chemoprevention use might alter vaccine efficacy, and adjusting for seasonal malaria chemoprevention use here did not alter vaccine efficacy estimates (data not shown).

MM^24^ has been administered as an adjuvant to tens of thousands of adults in vaccine trials for multiple diseases, including influenza and COVID-19,^25^, ^26^ but this is the first report on its use in children. The vaccine was well tolerated at both adjuvant doses. Humoral immunogenicity after three doses was good, antibody titres were two-times higher in group 2 compared with group 1 with the lower adjuvant dose. Levels were reboosted to comparable titres with a fourth dose at 12 months after the primary series of vaccinations.

R21 provides a high density of CSP epitopes on the particle surface, aiming to induce a high magnitude, and potentially better avidity, of antibodies to the central repeat of the CSP, by reducing antigenic competition with HBsAg sequences.^10^ HBsAg response rates have been very low in preclinical and UK phase 1/2 trials evaluating R21/MM, which have also shown high sterile efficacy rates of 63–78% using various vaccination regimens in controlled human malaria infection trials. This high efficacy in adults was observed with a low-dose R21 regimen of 10 μg.^13^, ^14^

The most advanced malaria vaccine candidate, RTS,S/AS01, has progressed to pilot implementation trials^8^ after a large phase 3 trial showed efficacy of 36% (95% CI 32–41) over a median follow-up of 48 months,^2^ but with 56% efficacy (97·5% CI 51–60) in children aged 5–17 months in the first year.^27^ Possible safety signals are being assessed further in current implementation studies.^7^ The phase 3 RTS,S/AS01 trials administered vaccines all year round, but with R21/MM there was planned seasonal vaccine administration, which might contribute to the higher observed efficacy.

Our trial site, Nanoro, Burkina Faso, was one of 11 sites to do the RTS,S/AS01 phase 3 trial. Efficacy with R21 and the higher adjuvant MM dose, administered largely before the malaria season, was 77% (95% CI 67–84), compared with 44% (37–50) reported for RTS,S/AS01 at this site, without planned or implemented seasonal administration, over 12 months of follow-up.^19^ This efficacy was achieved with use of 5 μg doses of R21 compared with 25 μg of RTS,S.

The most common local and systemic adverse events with RTS,S/AS01 across the phase 3 trial were pain at the injection site, after 12% (95% CI 11·4–13·4) of the primary series doses, and fever after 31% (30–33) of the primary series doses.^27^ These events appeared to be less frequent with R21/MM in this trial (table 2), 2% (group 1) and 3% (group 2) for pain and 12% (group 1) and 23% (group 2) for fever, consistent with safety datasets from R21/MM phase 1/2a trials.^13^, ^15^

We also found that antibodies to the central repeat of the CSP correlate strongly with protection, and the antibody titres in group 2 with the higher adjuvant dose were about six-times higher than in adults vaccinated in the UK,^17^ most of whom were protected in controlled human malaria infection trials.^14^ Levels of immunogenicity in adults vaccinated in Europe receiving RTS,S/AS01^18^, ^28^ and R21/MM^17^ appear similar. These levels of immunogenicity increase four times when RTS,S/AS01 is administered to African children,^2^ and six times for R21/MM in group 2 of this trial.

A surprising finding of the kinetics of antibody response and efficacy with RTS,S/AS01 was that antibody immunogenicity after a fourth booster dose peaked at only about half the level observed after the third vaccination, and efficacy waned considerably over time.^2^ This reduced reboosting of antibody levels might contribute to the increased incidence of malaria in the vaccinated children compared with controls observed in extended follow-up (years 4 to 7 after primary vaccinations) in the phase 2b and 3 RTS,S/AS01 trials.^29^, ^30^ After a fourth dose of R21/MM, antibody titres are comparable to those measured after the third dose, suggesting that efficacy with this newer vaccine candidate could be better maintained, at least through a second year of follow-up; this is currently being assessed.

This study has some limitations, including the short period of follow-up, although we have now extended the follow-up of this phase 2 trial. The age range of participants was limited to 5–17 months and a wider age range will be investigated in the future. There is also a need to evaluate larger numbers of participants to assess more fully the safety of a relatively new adjuvant formulation and document efficacy at sites with more perennial malaria transmission using non-seasonal vaccine administration.

An important advantage of R21/MM relates to its potential for large-scale manufacturing, which will be critical for the supply of hundreds of millions of doses of vaccine required annually for each birth cohort of children in malaria endemic regions of Africa. The R21 paediatric dose is just 5 μg.^31^ The saponin adjuvant, MM, lacks the monophosphoryl lipid A adjuvant component,^24^ which is found in other adjuvants^2^ and is less complex to manufacture, and this enables large-scale and low-cost supply of R21/MM. These factors contribute to the future potential of R21/MM as a vaccine in countries where malaria is a major public health concern.

Follow-up of this phase 2 trial is currently continuing for a second malaria season, after a booster dose in June, 2020, to determine the durability of this high vaccine efficacy. A phase 3 trial across five African sites of differing malaria transmission and seasonality is underway, with the aim of licensure of a safe, low-cost, high efficacy vaccine, which can substantially reduce the malaria disease burden.

## Data sharing

The study protocol is provided in the appendix. Anonymised participant data will be made available when the trial is complete, upon requests directed to a corresponding author. Proposals will be reviewed and approved by the sponsor, investigators, and collaborators on the basis of scientific merit. After approval of a proposal, data can be shared through a secure online platform after signing a data access agreement. All data will be made available for a minimum of 5 years from the end of the trial.

## Declaration of interests

AVSH and KJE are named as coinventors on patent applications related to R21. GG, LF, and JR are employees of Novavax, developers of the MM adjuvant, and US is an employee of the Serum Institute of India, codeveloper of the R21/MM vaccine. The other authors declare no competing interests.

## References

[1] White MT, Verity R, Griffin JT (2015). Immunogenicity of the RTS,S/AS01 malaria vaccine and implications for duration of vaccine efficacy: secondary analysis of data from a phase 3 randomised controlled trial. Lancet Infect Dis.

[2] RTS,S Clinical Trials Partnership (2015). Efficacy and safety of RTS,S/AS01 malaria vaccine with or without a booster dose in infants and children in Africa: final results of a phase 3, individually randomised, controlled trial. Lancet.

[3] Agnandji ST, Lell B, Fernandes JF (2012). A phase 3 trial of RTS,S/AS01 malaria vaccine in African infants. N Engl J Med.

[4] Gessner BD, Wraith DC, Finn A (2016). CNS infection safety signal of RTS,S/AS01 and possible association with rabies vaccine. Lancet.

[5] Vekemans J, Marsh K, Greenwood B (2011). Assessment of severe malaria in a multicenter, phase III, RTS, S/AS01 malaria candidate vaccine trial: case definition, standardization of data collection and patient care. Malar J.

[6] Klein SL, Shann F, Moss WJ, Benn CS, Aaby P (2016). RTS,S malaria vaccine and increased mortality in girls. MBio.

[7] Guerra Mendoza Y, Garric E, Leach A (2019). Safety profile of the RTS,S/AS01 malaria vaccine in infants and children: additional data from a phase III randomized controlled trial in sub-Saharan Africa. Hum Vaccin Immunother.

[8] Adepoju P (2019). RTS,S malaria vaccine pilots in three African countries. Lancet.

[9] Nkumama IN, O'Meara WP, Osier FHA (2017). Changes in malaria epidemiology in Africa and new challenges for elimination. Trends Parasitol.

[10] Collins KA, Snaith R, Cottingham MG, Gilbert SC, Hill AVS (2017). Enhancing protective immunity to malaria with a highly immunogenic virus-like particle vaccine. Sci Rep.

[11] Regules JA, Cummings JF, Ockenhouse CF (2011). The RTS,S vaccine candidate for malaria. Expert Rev Vaccines.

[12] Magnusson SE, Reimer JM, Karlsson KH, Lilja L, Bengtsson KL, Stertman L (2013). Immune enhancing properties of the novel Matrix-M™ adjuvant leads to potentiated immune responses to an influenza vaccine in mice. Vaccine.

[13] Datoo MS, Madhavan M, Bellamy D (2020). Looking ahead in malaria: R21/Matrix-M, an exciting new vaccine candidate. Am J Trop Med Hyg.

[14] Venkatraman N, Bowyer G, Edwards N (2017). High level efficacy in humans of a next-generation *Plasmodium falciparum* anti-sporozoite vaccine: R21 in Matrix-M (TM) adjuvant. Am J Trop Med Hyg.

[15] Njau IW, Datoo MS, Sang S (2020). A Phase Ib, open-label, age de-escalation, dose escalation study to evaluate the safety and tolerability of different doses of a candidate malaria vaccine adjuvanted R21 (R21/MM) in adults, young children and infants in Kilifi, Kenya. Am J Trop Med Hyg.

[16] Natama HM, Rovira-Vallbona E, Somé MA (2018). Malaria incidence and prevalence during the first year of life in Nanoro, Burkina Faso: a birth-cohort study. Malar J.

[17] Venkatraman N, Tiono AB, Bowyer G (2019). Phase I assessments of first-in-human administration of a novel malaria anti-sporozoite vaccine candidate, R21 in matrix-M adjuvant, in UK and Burkinabe volunteers. medRxiv.

[18] Rampling T, Ewer KJ, Bowyer G (2018). Safety and efficacy of novel malaria vaccine regimens of RTS,S/AS01B alone, or with concomitant ChAd63-MVA-vectored vaccines expressing ME-TRAP. NPJ Vaccines.

[19] RTS,S Clinical Trials Partnership (2014). Efficacy and safety of the RTS,S/AS01 malaria vaccine during 18 months after vaccination: a phase 3 randomized, controlled trial in children and young infants at 11 African sites. PLoS Med.

[20] Moorthy VS, Newman RD, Okwo-Bele JM (2013). Malaria vaccine technology roadmap. Lancet.

[21] Chandramohan D, Dicko A, Zongo I (2020). Seasonal malaria vaccination: protocol of a phase 3 trial of seasonal vaccination with the RTS,S/AS01_E_ vaccine, seasonal malaria chemoprevention and the combination of vaccination and chemoprevention. BMJ Open.

[22] WHO (2012). WHO policy recommendation: Seasonal malaria chemoprevention (SMC) for *Plasmodium falciparum* malaria control in highly seasonal transmission areas of the Sahel sub-region in Africa.

[23] Cairns ME, Sagara I, Zongo I (2020). Evaluation of seasonal malaria chemoprevention in two areas of intense seasonal malaria transmission: Secondary analysis of a household-randomised, placebo-controlled trial in Houndé district, Burkina Faso and Bougouni district, Mali. PLoS Med.

[24] Bengtsson KL, Karlsson KH, Magnusson SE, Reimer JM, Stertman L (2013). Matrix-M adjuvant: enhancing immune responses by ‘setting the stage’ for the antigen. Expert Rev Vaccines.

[25] Keech C, Albert G, Cho I (2020). Phase 1–2 trial of a SARS-CoV-2 recombinant spike protein nanoparticle vaccine. N Engl J Med.

[26] Portnoff AD, Patel N, Massare MJ (2020). Influenza hemagglutinin nanoparticle vaccine elicits broadly neutralizing antibodies against structurally distinct domains of H3N2 HA. Vaccines.

[27] Agnandji ST, Lell B, Soulanoudjingar SS (2011). First results of phase 3 trial of RTS,S/AS01 malaria vaccine in African children. N Engl J Med.

[28] Leroux-Roels G, Leroux-Roels I, Clement F (2014). Evaluation of the immune response to RTS,S/AS01 and RTS,S/AS02 adjuvanted vaccines: randomized, double-blind study in malaria-naïve adults. Hum Vaccin Immunother.

[29] Olotu A, Fegan G, Wambua J (2016). Seven-year efficacy of RTS,S/AS01 malaria vaccine among young African children. N Engl J Med.

[30] Tinto H, Otieno W, Gesase S (2019). Long-term incidence of severe malaria following RTS,S/AS01 vaccination in children and infants in Africa: an open-label 3-year extension study of a phase 3 randomised controlled trial. Lancet Infect Dis.

[31] Vaidyanathan G (2020). India will supply coronavirus vaccines to the world—will its people benefit?. Nature.

