# Supplementary material for: Efficacy of a low-dose candidate malaria vaccine, R21 in adjuvant Matrix-M, with seasonal administration to children in Burkina Faso: a randomised controlled trial
Source: Lancet. 2021 May 15;397(10287):1809–18. doi: 10.1016/S0140-6736(21)00943-0 (PMC8121760; doi:10.1016/S0140-6736(21)00943-0)

THE LANCET

Supplementary appendix

This appendix formed part of the original submission and has been peer reviewed. We post it as supplied by the authors.

Supplement to: Datoo MS, Natama MH, Somé A, et al. Efficacy of a low-dose candidate malaria vaccine, R21 in adjuvant Matrix-M, with seasonal administration to children in Burkina Faso: a randomised controlled trial. *Lancet* 2021; published online May 5. http://dx.doi.org/10.1016/S0140-6736(21)00943-0.

Supplementary Materials

Contents

Supplementary Materials 0

Supplementary Table 1: Demographics and characteristics of participants. 1

Supplementary Table 2a: Number of episodes of clinical malaria per treatment group over 12 months. 2

Supplementary Table 2b: Calculation of case rate per 1000 person-years (against all malaria episodes) 2

Supplementary Table 3: Serious Adverse Events up to 28 days following booster vaccination. 3

Supplementary Table 4: Asymptomatic *Plasmodium falciparum* infection. 4

Supplementary Table 5: Solicited Adverse Events in the 7 days following first three vaccinations........ 5

Supplementary Table 6: Solicited Adverse Events in the 7 days following booster vaccination........ 6

Supplementary Table 7: Unsolicited Adverse Events in Group 1. 7

Supplementary Table 8: Unsolicited Adverse Events in Group 2. 8

Supplementary Table 9: Unsolicited Adverse Events in Group 3. 9

Supplementary Figure 1: Kaplan – Meier estimates of the time to first episode of clinical malaria... 10

References 11

Supplementary Table 1: Demographics and characteristics of participants.

This includes all participants who received the primary series of vaccinations (3 doses). The eight participants who did not receive the third vaccination have not been included. Numbers are number of participants (%). Group 1 received 5µg R21/25µg MM, Group 2 received 5µg R21/50µg MM and Group 3, the control group, received Rabivax-S. **ITN – insecticide- treated net* [%]*A round of seasonal malaria chemoprevention (SMC) is 3 doses of treatment received per month*

	Group 1 n=146	Group 2 n=147	Group 3 n=149	Overall n=442
Age inmonths				
Mean (SD)	11.5 (3.7)	11.1 (3.8)	12.1 (3.8)	11.6 (3.8)
Age category inmonths				
5-9	50 (34.3)	57 (38.8)	40 (26.9)	147 (33.3)
10-12	19 (13.0)	21 (14.3)	19 (12.8)	59 (13.4)
>12	77 (52.7)	69 (46.9)	90 (60.4)	236 (53.4)
Sex				
Male	68 (46.6)	83 (56.5)	69 (46.3)	220 (49.8)
Female	78 (53.4)	64 (43.5)	80 (53.7)	222 (50.2)
Indoor residual spraying				
Yes	19 (13.0)	18 (12.2)	28 (18.8)	65 (14.7)
Missing		1 (0.7)		1 (0.2)
Adequate bed net use				
Yes	125 (85.6)	130 (88.4)	128 (85.9)	383 (86.7)
Missing		1 (0.7)		1 (0.2)
Bed net use Absent				
ITN* no holes	15 (10.3)	10 (6.8)	17 (11.4)	42 (9.5)
ITN* with holes	124 (84.9)	128 (87.1)	126 (84.6)	378 (85.5)
Missing	7 (4.8)	8 (5.4)	6 (4.0)	21 (4.8)
		1 (0.7)		1 (0.2)
At least one round[%] of SMC				
Yes	107 (73.3)	91 (61.9)	102 (68.5)	300 (67.9)
Number of rounds[%] of SMC				
0	39 (26.7)	56 (38.1)	47 (31.5)	142 (32.1)
1	75 (51.4)	57 (38.8)	78 (52.4)	210 (47.5)
2	26 (17.8)	28 (19.1)	22 (14.8)	76 (17.2)
3	4 (2.7)	5 (3.4)	2 (1.3)	11 (2.5)
4	1 (0.7)	1 (0.7)	0	2 (0.5)
5	1 (0.7)	0	0	1 (0.2)
Z score				
≥-3SD and <-2SD	32 (21.9)	29 (19.7)	33 (22.2)	94 (21.3)
≥-2SD and <-1SD	63 (43.2)	53 (36.1)	55 (36.9)	171 (38.7)
≥-1SD and <median	37 (25.3)	47 (32.0)	40 (26.9)	124 (28.1)
≥ median and < 1SD	10 (6.9)	17 (11.6)	17 (11.4)	44 (10.0)
≥ 1SD and < 2SD	4 (2.7)	1 (0.7)	4 (2.7)	9 (2.0)

Supplementary Table 2a: Number of episodes of clinical malaria per treatment group over 12 months.

Numbers are number of participants (%). Those who withdrew before 12 month follow-up have not been included as they did not have the potential for as many episodes as participants followed up for 12 months. Group 1 received 5μg R21/25μg MM, Group 2 received 5μg R21/50μg MM and Group 3, the control group, received Rabivax-S.

Number of episodes	Group 1 (n=134)<br>n (%)	Group 2 (n=139)<br>n (%)	Group 3 (n=142)<br>n (%)
0	85 (63.4)	101 (72.7)	39 (27.5)
1	30 (22.4)	25 (18.0)	33 (22.5)
2	13 (9.7)	8 (5.8)	34 (23.9)
3	6 (4.5)	3 (2.2)	20 (14.1)
4	0	1 (0.7)	6(4.2)
5	0	1 (0.7)	6 (4.2)
6	0	0	2 (1.4)
7	0	0	1 (0.7)
8	0	0	1 (0.7)
Total number of episodes	74	59	242

Supplementary Table 2b: Calculation of case rate per 1000 person-years (against all malaria episodes)

Case incidence rates for all episodes were calculated using negative binomial regression over 12 months for Groups 1 to 3[1]. Calculation of the number of cases averted in Groups 1 and 2, per 1000 person years, shows 1393 [95% CI 1043 – 1744] cases averted in Group 1 and 1523 [1172 – 1875] in Group 2 over 12 months [2]. Note that the number of events is larger here than in Table 2a above because that table excludes children who did not complete follow-up to 12 months (on the basis that they had less time to experience events). The regression method used here for Table 2b takes account of person-time so such exclusion is not required when looking at multiple events.

Group	Person-years at risk	Events	Rate per 1000 person-years (95% CI)	Crude Rate Ratio (95% CI)	Protective Efficacy % (95% CI)	Wald Test P-value
Group 1	120.5	75	622.6 (496.5-780.7)	0.30 (0.22-0.41)	69.6 (58.7-77.6)	<0.001
Group 2	121.8	60	492.7 (382.5-634.5)	0.24 (0.18-0.34)	75.7 (66.3-82.4)	<0.001
Group 3	124.0	250	2015.9 (1780.8-2281.9)			

Supplementary Table 3: Serious Adverse Events up to 28 days following booster vaccination.

All terms are coded according to MedDRA preferred terms (PT). Group 1 received 5µg R21/25µg MM, Group 2 received 5µg R21/50µg MM and Group 3, the control group, received Rabivax-S. All SAEs were reviewed by the DSMB. Causality was assigned according to criteria in Table 12 in the protocol. IMP – Investigational Medicinal Product.

Group	Number of vaccinations	Serious Adverse Event (SAE)	Outcome	Number of days following last vaccination to start of SAE	Causality to IMP
1	1	Malaria	Fatal	17	Not related
1	3	Anaemia secondary to pneumonia and enteritis	Resolved	161	Not related
1	3	Acute lymphocytic leukaemia	Fatal	210	Not related
1	4	Meningitis	Resolved	19	Not related
2	2	Bronchiolitis	Resolved	12	Not related
2	3	Diarrhoea haemorrhagic and dehydration	Resolved	205	Not related
3	3	Malnutrition and Anaemia	Resolved	184	Not related

Supplementary Table 4: Asymptomatic *Plasmodium falciparum* infection.

Cross-sectional asymptomatic *P.falciparum* infection was analysed at both 6 and 12 months from 14 days following the third vaccination. Primary analysis was based on a modified intention-to-treat population. Asymptomatic infection was defined as the presence of axillary temperature <37.5°C, absence of history of fever within the last 24 hours, and *P.falciparum* parasite density >0 parasites/µl. Group 1 received 5µg R21/25µg MM, Group 2 received 5µg R21/50µg MM and Group 3, the control group, received Rabivax-S. These were analysed using a log binomial regression model, including randomised group as a covariate. Relative risks and 95% confidence intervals were reported comparing groups 1 and 3 and groups 2 and 3. *adjusted for confounding factors of sex, age category (5-9 months, 10-12 months and >12 months) and adequate insecticide-treated bed net use .

			Unadjusted			Adjusted*		
Group	Time point	Number with at least one episode of asymptomatic malaria (%)	RR	95% CI	P-value	RR	95% CI	P-value
Group 1	6 months	12/140 (8.6)	0.44	0.23-0.84	0.012	0.45	0.24-0.84	<0.0001
	12 months	3/132 (2.3)	0.54	0.14-2.11	0.373	0.56	0.14-2.21	0.412
Group 2	6 months	13/145 (9.0)	0.46	0.25-0.86	0.013	0.47	0.25-0.88	0.017
	12 months	2/141 (1.4)	0.34	0.07-1.64	0.177	0.37	0.08-1.85	0.229
Group 3	6 months	28/147 (19.1)						
	12 months	6/142 (4.2)						

Supplementary Table 5: Solicited Adverse Events in the 7 days following first three vaccinations.

Numbers are number of participants (%). For each participant, the event has been counted only once at its highest grading recorded over the 7 days. Group 1 received 5µg R21/25µg MM, Group 2 received 5µg R21/50µg MM and Group 3, the control group, received Rabivax-S. 150 participants in each group received the first dose of the vaccination series. 149 participants in Group 1, 147 participants in Group 2 and 149 participants in Group 3 received a second dose. 146 participants in Group 1, 147 participants in Group 2 and 149 participants in Group 3 received a third dose. The grading of adverse events is as specified in Tables 9 – 11 in the protocol.

**Adverse Event**	**Dose number**	**No. of participants in Group 1 (%)**			**No. of participants in Group 2 (%)**			**No. of participants in Group 3 (%)**		
		Mild	Moderate	Severe	Mild	Moderate	Severe	Mild	Moderate	Severe
Redness	1	2 (1.3)	0 (0)	0 (0)	1 (0.7)	0 (0)	0 (0)	0 (0)	0 (0)	0 (0)
	2	8 (5.4)	2 (1.3)	0 (0)	12 (8.2)	2 (1.4)	0 (0)	2 (1.3)	0 (0)	0 (0)
	3	1 (0.7)	1 (0.7)	0 (0)	2 (1.4)	0 (0)	0 (0)	1 (0.7)	0 (0)	0 (0)
Swelling	1	3 (2)	0 (0)	0 (0)	4 (2.7)	1 (0.7)	0 (0)	2 (1.3)	0 (0)	0 (0)
	2	13 (8.7)	1 (0.7)	0 (0)	19 (12.9)	4 (2.7)	0 (0)	10 (6.7)	0 (0)	0 (0)
	3	7 (4.8)	1 (0.7)	0 (0)	10 (6.8)	1 (0.7)	0 (0)	4 (2.7)	0 (0)	0 (0)
Pain	1	5 (3.3)	1 (0.7)	0 (0)	9 (6)	0 (0)	0 (0)	3 (2)	0 (0)	0 (0)
	2	3 (2)	0 (0)	0 (0)	3 (2)	0 (0)	0 (0)	0 (0)	0 (0)	0 (0)
	3	0 (0)	0 (0)	0 (0)	0 (0)	0 (0)	0 (0)	0 (0)	0 (0)	0 (0)
Fever	1	12 (8)	2 (1.3)	0 (0)	21 (14)	7 (4.7)	0 (0)	10 (6.7)	3 (2)	0 (0)
	2	11 (7.4)	7 (4.7)	0 (0)	28 (19)	16 (10.9)	0 (0)	7 (4.7)	0 (0)	0 (0)
	3	11 (7.5)	6 (4.1)	1 (0.7)	21 (14.3)	7 (4.8)	1 (0.7)	9 (6)	7 (4.7)	0 (0)
Loss of appetite	1	1 (0.7)	0 (0)	0 (0)	2 (1.3)	0 (0)	0 (0)	1 (0.7)	0 (0)	0 (0)
	2	0 (0)	0 (0)	0 (0)	3 (2)	0 (0)	0 (0)	1 (0.7)	0 (0)	0 (0)
	3	0 (0)	0 (0)	0 (0)	0 (0)	0 (0)	0 (0)	0 (0)	0 (0)	0 (0)
Irritability	1	1 (0.7)	1 (0.7)	0 (0)	4 (2.7)	1 (0.7)	0 (0)	0 (0)	0 (0)	0 (0)
	2	0 (0)	0 (0)	0 (0)	2 (1.4)	2 (1.4)	0 (0)	0 (0)	0 (0)	0 (0)
	3	0 (0)	0 (0)	0 (0)	0 (0)	0 (0)	0 (0)	0 (0)	1 (0.7)	0 (0)
Drowsiness	1	0 (0)	0 (0)	0 (0)	3 (2)	0 (0)	0 (0)	1 (0.7)	0 (0)	0 (0)
	2	1 (0.7)	1 (0.7)	0 (0)	4 (2.7)	2 (1.4)	0 (0)	1 (0.7)	1 (0.7)	0 (0)
	3	0 (0)	0 (0)	0 (0)	0 (0)	0 (0)	0 (0)	0 (0)	0 (0)	0 (0)

Supplementary Table 6: Solicited Adverse Events in the 7 days following booster vaccination.

Numbers are number of participants (%). For each participant, an event has been counted only once at its highest grading recorded over the 7 days. Group 1 received 5µg R21/25µg MM, Group 2 received 5µg R21/50µg MM and Group 3, the control group, received Rabivax-S. 132 participants in Group 1, 138 participants in Group 2 and 140 participants in Group 3 received a fourth, booster dose. The grading of adverse events is as specified in Tables 9 – 11 in the protocol.

**Adverse Event**	**Grade**	**No. of participants in Group 1 (%)**	**No. of participants in Group 2 (%)**	**No. of participants in Group 3 (%)**
Redness	Mild	3 (2.3)	0	0
	Moderate	0	0	0
	Severe	0	0	0
Swelling	Mild	3 (2.3)	0	0
	Moderate	0	0	0
	Severe	0	0	0
Pain	Mild	4 (3.0)	0	0
	Moderate	0	0	0
	Severe	0	0	0
Fever	Mild	13 (9.9)	24 (17.4)	7 (5.0)
	Moderate	6 (4.6)	10 (7.3)	1 (0.7)
	Severe	0	0	0
Loss of appetite	Mild	0	1 (0.7)	1 (0.7)
	Moderate	0	0	0
	Severe	0	0	0
Irritability	Mild	1 (0.8)	1 (0.7)	1 (0.7)
	Moderate	0	0	0
	Severe	0	0	0
Drowsiness	Mild	0	1 (0.7)	0
	Moderate	0	1 (0.7)	0
	Severe	0	0	0

Supplementary Table 7: Unsolicited Adverse Events in Group 1.

These participants received up to 3 doses of 5µg R21/25µg Matrix-M, 4 weeks apart. Numbers are number of participants. Unsolicited adverse events were collected for 28 days following each dose of vaccination. All terms are coded according to MedDRA preferred term (PT). Severity grading of Adverse Events was based on activity/medical intervention/therapy required as per Table 8 in the protocol.

	Mild				Moderate			
AE category	Dose 1	Dose 2	Dose 3	Total	Dose 1	Dose 2	Dose 3	Total
Amoebiasis	3	4	6	13	3	3	7	13
Bronchiolitis	4	1	1	6	4	2	3	9
Bronchitis	0	0	0	0	0	3	0	3
Conjunctivitis	3	2	2	7	1	1	0	2
Cough	1	1	0	2	0	0	0	0
Dermatitis	2	1	2	5	2	3	4	9
Diarrhoea	2	2	0	4	3	0	0	3
Ear Infection	1	2	0	3	3	4	4	11
Eczema	0	0	1	1	0	0	1	1
Fungal Skin Infection	0	0	1	1	0	1	0	1
Furuncle	0	1	1	2	0	0	0	0
Gastroenteritis	2	4	3	9	1	1	7	9
Influenza	0	0	0	0	0	2	0	2
Laryngitis	0	0	0	0	0	1	0	1
Malaria	7	0	0	7	6	1	11	18
Oral Candidiasis	1	0	0	1	3	4	1	8
Pharyngitis	11	5	5	21	15	13	10	38
Pneumonia	0	0	1	1	3	1	0	4
Prurigo	0	0	3	3	0	1	1	2
Rash Pustular	1	1	2	4	1	4	2	7
Rhinitis	5	6	5	16	6	6	4	16
Staphylococcal Skin Infection	0	0	0	0	2	2	1	5
Subcutaneous Abscess	0	0	0	0	1	0	0	1
Tinea Infection	0	0	1	1	0	0	0	0
Urinary Tract Infection	0	0	0	0	0	1	0	1
Wound	0	1	1	2	0	0	0	0
Total	43	31	35	109	54	54	56	164

Supplementary Table 8: Unsolicited Adverse Events in Group 2.

These participants received up to 3 doses of 5µg R21/50µg Matrix-M, 4 weeks apart. Numbers are number of participants. Unsolicited adverse events were collected for 28 days following each dose of vaccination. All terms are coded according to MedDRA preferred term (PT). Severity grading of AEs was based on activity/medical intervention/therapy required as per Table 8 in the protocol. *There was one participant with Bronchiolitis classified as severe.

	Mild				Moderate			
AE category	Dose 1	Dose 2	Dose 3	Total	Dose 1	Dose 2	Dose 3	Total
Amoebiasis	4	1	4	9	6	1	7	14
Bronchiolitis	3	2	1	6	1	3	2	6
Bronchitis	0	0	0	0	0	1	0	1
Conjunctivitis	2	2	2	6	4	0	2	6
Cough	4	0	0	4	1	0	0	1
Dermatitis	2	2	4	8	7	2	1	10
Dermatitis Diaper	0	0	0	0	0	1	0	1
Diarrhoea	2	0	0	2	0	0	1	1
Ear Infection	0	0	0	0	1	5	8	14
Eczema	0	0	1	1	0	0	0	0
Fungal Skin Infection	1	1	1	3	0	0	1	1
Furuncle	0	1	1	2	0	1	0	1
Gastroenteritis	4	3	3	10	5	1	4	10
Hordeolum	1	0	0	1	0	0	0	0
Influenza	0	0	0	0	2	2	1	5
Malaria	6	1	0	7	3	2	5	10
Oral Candidiasis	0	1	0	1	1	0	1	2
Parotitis	0	0	0	0	0	1	0	1
Pharyngitis	13	3	2	18	12	24	14	50
Pneumonia	1	0	0	1	3	2	2	7
Prurigo	0	3	1	4	1	1	3	5
Rash	0	0	0	0	1	0	0	1
Rash Pustular	1	2	2	5	0	1	2	3
Rhinitis	2	4	4	10	4	0	3	7
Staphylococcal Skin Infection	0	0	0	0	1	2	2	5
Subcutaneous Abscess	0	0	0	0	1	0	0	1
Thermal Burn	0	1	0	1	0	0	0	0
Tinea Infection	0	0	0	0	0	1	0	1
Urinary Tract Infection	0	0	2	2	0	0	0	0
Wound	0	1	1	2	0	0	0	0
Total	46	28	29	103	54	51	59	164

Supplementary Table 9: Unsolicited Adverse Events in Group 3.

These participants received up to 3 doses of the control vaccine (Rabivax -S), 4 weeks apart. Numbers are number of participants. Unsolicited adverse events were collected for 28 days following each dose of vaccination. All terms are coded according to MedDRA preferred term (PT). Severity grading of AEs was based on activity/medical intervention/therapy required as per Table 8 in the protocol.

	Mild				Moderate			
AE category	Dose 1	Dose 2	Dose 3	Total	Dose 1	Dose 2	Dose 3	Total
Amoebiasis	2	5	4	11	1	8	5	14
Bronchiolitis	2	1	4	7	4	0	0	4
Bronchitis	0	1	0	1	0	0	0	0
Conjunctivitis	3	4	1	8	3	0	1	4
Cough	2	0	0	2	1	0	0	1
Dermatitis	1	1	6	8	0	3	1	4
Dermatitis Diaper	0	1	0	1	1	0	0	1
Diarrhoea	1	0	0	1	0	0	2	2
Ear Infection	0	4	2	6	1	2	8	11
Eczema	0	1	0	1	0	0	0	0
Furuncle	1	0	0	1	0	0	0	0
Gastroenteritis	0	2	2	4	4	5	7	16
Influenza	0	0	0	0	1	2	0	3
Keratitis	0	0	0	0	1	0	0	1
Malaria	6	0	2	8	1	5	21	27
Oral Candidiasis	3	1	0	4	1	2	2	5
Parotitis	0	0	0	0	0	0	1	1
Pharyngitis	8	6	5	19	14	14	12	40
Pneumonia	0	1	0	1	5	1	1	7
Prurigo	0	0	2	2	0	2	3	5
Rash Pustular	0	0	3	3	1	0	2	3
Rhinitis	5	6	5	16	9	1	3	13
Staphylococcal Skin Infection	0	1	0	1	0	1	0	1
Subcutaneous Abscess	0	0	0	0	0	0	1	1
Wound	0	0	1	1	1	0	0	1
Total	34	35	37	106	49	46	70	165

A

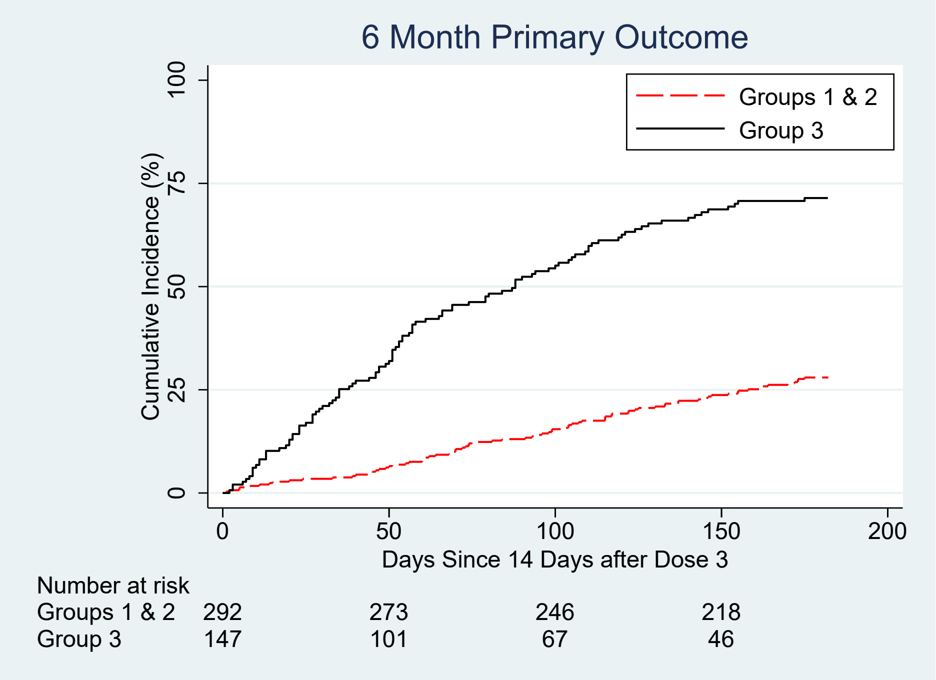

B

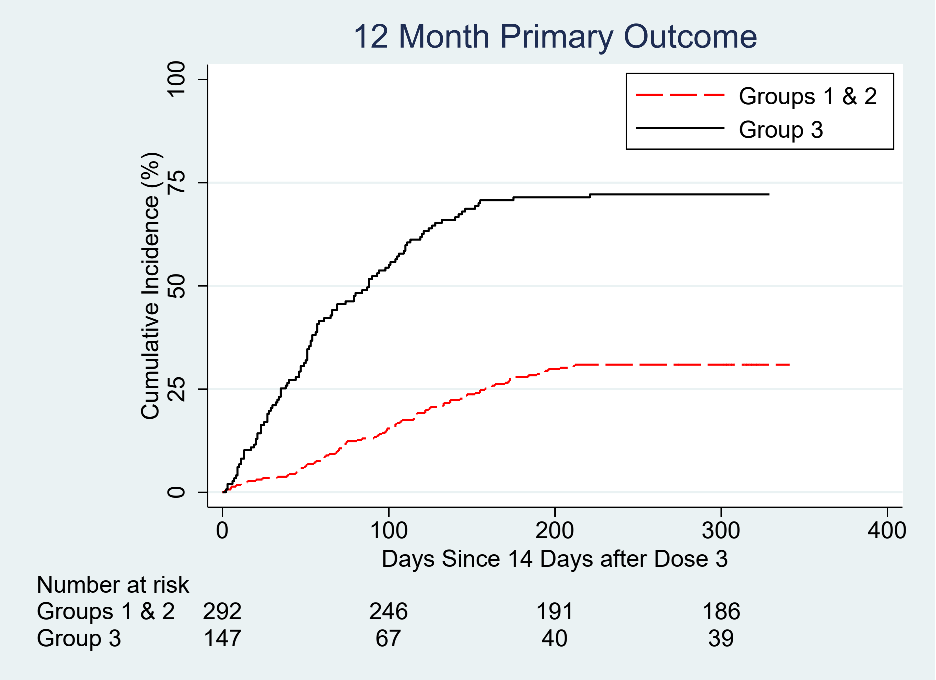

Supplementary Figure 1: Kaplan – Meier estimates of the time to first episode of clinical malaria according to the primary case definition

Primary analysis was based on a modified intention-to-treat population. R21/Matrix-M groups have here been combined (Group 1 and 2). Panel A shows the data beginning from 14 days to 6 months after third vaccination. Panel B shows the data beginning from 14 days to 12 months after third vaccination. Group 1 received 5µg R21/25µg MM, Group 2 received 5µg R21/50µg MM and Group 3, the control group, received Rabivax-S vaccinations.

References

1. RTS,S Clinical Trials Partnership. Efficacy and safety of the RTS,S/AS01 malaria vaccine during 18 months after vaccination: a phase 3 randomized, controlled trial in children and young infants at 11 African sites. PLoS Medicine. 2014;11(7):e1001685. .

2. Xu Y, Cheung YB, Lam KF, Milligan P. 2010 A simple approach to the estimation of incidence rate difference. Am J Epidemiol **172**: 334-343.

A Phase Ib/IIb randomised controlled trial of the safety, immunogenicity and efficacy of a candidate malaria vaccine, R21 adjuvanted with Matrix-M (R21/MM), in 5-17 month old children in Nanoro, Burkina Faso

Study reference:	VAC 076
Protocol Version:	4.0
Date:	9th September 2019
OXTREC Number:	19-19
Sponsor:	University of Oxford
Funding body:	EDCTP
Author(s):	**IRSS-URCN:** Athanase M. Some, Hermann Sorgho, Halidou Tinto<br>**UOXF:** Mehreen Datoo, Katie Ewer, Adrian VS Hill

 © University of Oxford, 2019

Statement of Compliance

The trial will be conducted in compliance with the protocol, International Conference on Harmonisation Good Clinical Practice Guideline E6 (R1) (ICH-GCP) and the applicable regulatory requirements.

Signatures

"I have read this protocol and agree to abide by all provisions set forth therein. I agree to comply with the principles of the Declaration of Helsinki and the International Conference on Harmonisation Tripartite Guideline on Good Clinical Practice."

PROTOCOL SIGNATURE SHEET

Principal Investigator:

_______________________________ Date:

Confidentiality Statement
This document contains confidential information that must not be disclosed to anyone other than the Sponsor, the Investigator Team, members of the Independent Ethics Committee and the Burkina regulatory authority. This information cannot be used for any purpose other than the evaluation or conduct of the clinical investigation without the prior written consent of Professor Adrian Hill.

 © University of Oxford, 2019

CONTENTS

Contents 3
Key Roles and General Information 4
List of abbreviations 6
1. Study synopsis 8
2. Background Information 15
3. Rationale 32
4. Objectives 34
5. Description and justification of study design 36
6. Inclusion and Exclusion Criteria 43
7. Investigational Medicinal Products 46
8. Study schedule and procedures 48
9. Assessment of scientific objectives 61
10. Safety Reporting 64
11. Data Handling and Record Keeping 70
12. Data Access and Quality Assurance 72
13. Ethical Considerations 74
14. Indemnity/Compensation/Insurance 76
15. References 77

 © University of Oxford, 2019

KEY ROLES AND GENERAL INFORMATION

**Trial Centre:**	Institut de Recherche en Sciences de la Sante - Clinical Research Unit of Nanoro (IRSS-URCN)<br>Nanoro<br>Burkina Faso
**Principal Investigator:**	Dr Halidou Tinto<br>Institut de Recherche en Sciences de la Sante - Clinical Research Unit of Nanoro, (IRSS-URCN)<br>Nanoro<br>Burkina Faso
**Study Physician**	Dr Athanase M.Some
**Co-Investigator(s):**	***Clinical Research Unit of Nanoro, Institut de Recherche en Sciences de la Sante (IRSS-URCN)***<br>— Dr Hermann Sorgho<br>— Dr Magloire Natama<br><br>***United Kingdom (Jenner Institute, Nuffield Dept. of Clinical Medicine, University of Oxford)***<br>— Prof Adrian Hill<br>— Dr Mehreen Datoo<br>— Prof Katie Ewer<br>— Dr Alison Lawrie<br>— Dr Rachel Roberts<br><br>Centre for Clinical Vaccinology and Tropical Medicine<br>University of Oxford Churchill Hospital, Oxford OX3 7LE
**Local Safety Monitor:**	
**Data Safety and Monitoring Board:**	Chair: Roma Chilengi<br><br>DSMB members: Brian Angus, Kwaku Poku Asante, Greg Fegan, William Macharia
**Project Managers (Oxford):**	Ms Rachel Roberts<br><br>Centre for Clinical Vaccinology and Tropical Medicine

 © University of Oxford, 2019

University of Oxford Churchill Hospital, Oxford OX3 7LJ

Sponsor: University of Oxford
University Offices
Wellington Square
Oxford
OX1 2JD

External Monitor: Pharmalys
94-95 Sacre Coeur Pyrotechnie
Cite Kaur Gorgui
Dakar
Senegal

© University of Oxford, 2019

LIST OF ABBREVIATIONS

**AE**	Adverse Event
**Ab**	Antibody
**ALT**	Alanine transaminase
AST	Aspartate Aminotransferase
**BP**	Blood Pressure
**CBF**	Clinical Bio-Manufacturing Facility
**CI**	Chief Investigator
**CHMI**	Controlled Human Malaria Infection
**CS**	Circumsporozoite
**CSP**	Circumsporozoite Protein
**DSMB**	Data and Safety Monitoring Board
**EPI**	Expanded Programme of Immunisation
**FBC**	Full Blood Count
**GCP**	Good Clinical Practice
**HBsAg**	Hepatitis B surface antigen
**HCV**	Hepatitis C Virus
**HIV**	Human Immunodeficiency Virus
**HR**	Heart Rate
**IC**	Informed Consent
**ICH**	International Conference on Harmonisation
**LSM**	Local safety monitor
**OPD**	Out-patient department
**PI**	Principal Investigator
**R21**	Jenner Institute malaria vaccine construct evaluated in pre-clinical trials
**R21c**	A clinical grade R21 particle was manufactured by clinical bio-manufacturing facility (CBF), University of Oxford. At the C-terminus of R21 a four amino acid sequence was added, EPEA (C-tag), which

 © University of Oxford, 2019

	was required for efficient immunochromatographic purification of R21; has been evaluated in early phase trials
**R21/MM**	This vaccine is manufactured by Serum Institute India, adjuvanted to Matrix M, and in the manufacturing of the vaccine no C-tag was required for the purification process and so this is not present in R21; yet to be evaluated in human subjects
**SAE**	Serious Adverse Event
**SOP**	Standard Operating Procedure
**SUSAR**	Suspected Unexpected Serious Adverse Reaction

 © University of Oxford, 2019

1. Study synopsis

**Trial Title**	**A Phase Ib/IIb randomised controlled trial of the safety, immunogenicity and efficacy of a candidate malaria vaccine, R21 adjuvanted with Matrix-M (R21/MM), in 5-17 month old children in Nanoro, Burkina Faso**
**Trial Identifier**	VAC 076
**Clinical phase**	IIb
**Investigational medicinal products**	1. R21: Protein particle malaria vaccine candidate<br>2. Matrix-M: Saponin based vaccine adjuvant
Form	R21 (liquid); Matrix-M (liquid)
Dose per administration	• R21: 5µg<br>• Matrix-M: 25µg or 50µg
Route of administration	Intramuscular needle injection into the anterolateral thigh
**Principal Investigator**	Dr Halidou Tinto
**Trial Centre**	Institut de Recherche en Sciences de la Santé - Unité de Recherche Clinique de Nanoro (IRSS-URCN), Burkina Faso
**Planned Trial Period**	Q2 2019-Q3 2021
**Study Duration**	28 months
**Subject Duration**	24 months from Day 0
**Objectives**	
Primary Objective	To assess the protective efficacy against clinical malaria of R21 adjuvanted with Matrix-M in 5-17 months old children living in a malaria-endemic area, for 6 months after the third vaccination.

 © University of Oxford, 2019

Secondary Objectives	*<u>Duration of Protective efficacy against clinical malaria</u>*<br>To assess the protective efficacy against clinical malaria of R21 adjuvanted with Matrix-M in 5-17 months old children living in a malaria-endemic area, for 12 months after administration of the third dose of vaccine.<br><br>To assess the protective efficacy against clinical malaria of R21 adjuvanted with Matrix-M in 5-17 months old children living in a malaria-endemic area, for 6 months after a booster vaccination.<br><br>To assess the protective efficacy against clinical malaria of R21 adjuvanted with Matrix-M in 5-17 months old children living in a malaria-endemic area, for 12 months after a booster vaccination.<br><br>*<u>Efficacy against asymptomatic P. falciparum infection</u>*<br>To assess the protective efficacy against asymptomatic *P. falciparum* infection of R21 adjuvanted with Matrix-M in 5-17 months old children living in a malaria-endemic area, at 12 months after administration of the third dose of vaccine.<br><br>To assess the protective efficacy against asymptomatic *P. falciparum* infection of R21 adjuvanted with Matrix-M in 5-17 months old children living in a malaria-endemic area, at 12 months after administration of the booster dose of vaccine.<br><br>*<u>Safety Objectives</u>*<br>To assess the safety and reactogenicity of R21 adjuvanted with Matrix-M in 5-17 months old children living in a malaria-endemic area, in the month following each vaccination and at 12 months after administration of the third dose of vaccine.<br><br>To assess the safety and reactogenicity of R21 adjuvanted with Matrix-M in 5-17 months old children living in a malaria-endemic area, in the month following each vaccination and at 12 months after administration of the booster dose of vaccine.<br><br>*<u>Immunogenicity Objectives</u>*<br>To assess the humoral immunogenicity of R21 adjuvanted with Matrix-M in 5-17 months old children living in a malaria-endemic area.

 © University of Oxford, 2019

Exploratory Objective	*<u>Efficacy against incident cases of severe malaria</u>*<br>1. To assess the protective efficacy against severe malaria of R21 adjuvanted with Matrix-M in 5-17 months old infants living in a malaria-endemic area, at 6 months after administration of the third dose of vaccine.<br>2. To assess the protective efficacy against severe malaria of R21 adjuvanted with Matrix-M in 5-17 months old infants living in a malaria-endemic area, at 6 months after administration of the booster dose of vaccine.<br>3. To evaluate cellular immunogenicity and other exploratory immunological end points.
**Population**	Healthy Burkinabe infants and children aged 5 to 17 months at enrolment
**Planned Sample**	450 participants
**Vaccination Schedule**	R21 adjuvated with Matrix-M on Day 0, Day 28 and Day 56; or Rabies vaccine on Day 0, Day 28 and Day 56. All groups will receive a fourth booster vaccination before the malaria season commences the following year.
**Follow-up duration**	26 months from Day 0
**Blood Sampling Schedule**	Screening , Day 7, Day 84, Day 236, Day 421, Screening prior to Boost vaccination, Boost vaccination + 28 days, Boost vaccination + 236 days, Boost vaccination + 421 days
**Endpoints**	***Efficacy endpoints***<br><br>- *Primary case definition of clinical malaria episode*<br>- *Secondary case definitions of clinical malaria episode*<br>- *Primary case definition of asymptomatic P. falciparum infection*<br>- *Primary case definition of severe malaria*<br>- *Secondary case definitions of severe malaria*<br><br>***Safety endpoints***<br><br>SAEs occurring from first vaccination until the end of the study<br><br>Local and systemic solicited and unsolicited adverse events, considered possibly, probably, or definitely related to vaccination, occurring from first vaccination until 28 days post third vaccination (study day 84).<br><br>Local and systemic solicited and unsolicited adverse events, considered possibly, probably, or definitely related to vaccination, occurring from boost vaccination until 28 days post boost vaccination.<br><br>***Immunogenicity endpoints***<br><br>• Comparison of immunogenicity (antibody responses to CSP) in the

R21/MM vaccination group with those in the rabies vaccine group and the durability of responses.

- ELISA to quantify antibodies to the vaccine components (regions of the CS antigen including the NANP repeat region and other elements of the protein as well as antiHBs).
- Flow cytometry assays with intracellular cytokine staining to enumerate and functionally characterise immune cell populations such as effector and memory T cells (e.g. $CD4^+$ and $CD8^+$), T follicular helper cells, regulatory T cells, B cells, plasma cells and dendritic cells
- ELISPOT for enumeration of antibody-secreting cells (e.g. B and plasma cells)

**Study Design**	Double-blinded, randomized controlled study

Schematic of Study Design

Week	0	4	8	Boost
**Group 1 n=150**	5µg R21/25µg Matrix-M	5µg R21/25µg Matrix-M	5µg R21/25µg Matrix-M	5µg R21/25µg Matrix-M
**Group 2 n=150**	5µg R21/50µg Matrix-M	5µg R21/50µg Matrix-M	5µg R21/50µg Matrix-M	5µg R21/50µg Matrix-M
**Group 3 n=150**	(Control vaccine)	(Control vaccine)	(Control vaccine)	(Control vaccine)

 © University of Oxford, 2019

Study visit number	S	1	2-7	8	9	10-15	16	17	18-23	24	25	26	27	28	29	30	31
Clinic visit	X	X		X	X		X	X		X	X					X	X
Home visit			X			X			X			X	X	X	X		
Day of visit	D-30-D-1	D0	D1-6	D7	D28	D29-34	D35	D56	D57-62	D63	D84	D114	D144	D174	D204	D236	D421
Window period				+/- 1	+/- 3		+/- 1	+/- 3		+/- 1	+/- 3	+/- 7	+/- 7	+/- 7	+/- 7	+/- 28	+/- 56
Vaccination		X			X			X									
Inclusion/Exclusion criteria	X	X			X			X									
Informed consent	X																
Medical history	X	(X)		(X)	(X)		(X)	(X)		(X)	(X)	(X)	(X)	(X)	(X)	(X)	(X)
Physical examination	X	(X)		(X)	(X)		(X)	(X)		(X)	(X)	(X)	(X)	(X)	(X)	(X)	(X)
Review contraindications to vaccination		X			X			X									
Recording of concomitant medication	X	X		X	X		X	X		X	X	X	X	X	X	X	X
Recording of solicited AEs			X	X		X	X		X	X							
Recording of unsolicited AEs			X	X	X	X	X	X	X	X	X						
Recording of SAEs			X	X	X	X	X	X	X	X	X	X	X	X	X	X	X
Rectal swab/faecal sample		X															
Blood film for Plasmodium species	X															X	X
Blood film for Plasmodium species if axillary temp ≥37.5				X	X		X	X		X	X	X	X	X	X		

 © University of Oxford, 2019

and/or history of fever within last 24 hours																
**Blood sampling**	X				X					X					X	X

Table 1: Timeline of study visits and procedures for participants in Groups 1, 2 & 3
S: Screening Visit; X: procedure takes place, (X): procedure takes place as required at the discretion of the investigators; D : Day.

Study visit number	S (B)	B+1	B+2-7	B+8	B+9	B+10	B+11
Clinic visit	X	X		X	X	X	X
Home visit			X				
Day of visit	B-30-B-1	B0	B1-6	B7	B28	B168	B336
Window period				+/- 1	+/- 3	+/- 28	+/- 28
Vaccination		X					
Inclusion/Exclusion criteria	X	X					
Informed consent	X						
Medical history	X	(X)	(X)	(X)	(X)	(X)	(X)
Physical examination	X	(X)	(X)	(X)	(X)	(X)	(X)
Review contraindications to vaccination		X					
Recording of concomitant medication	X	X	X	X	X	X	X
Recording of solicited AEs			X	X			
Recording of unsolicited AEs			X	X	X		
Recording of SAEs			X	X	X	X	X
Rectal swab/faecal		X					

 © University of Oxford, 2019

**sample**							
**Blood film for Plasmodium species**	X					X	X
**Blood film for Plasmodium species if axillary temp** ≥37.5 and/or history of fever within last 24 hours		X		X	X		
**Blood sampling**	X				X	X	X

Table 2: Timeline of study visits and procedures for participants in Groups 1, 2 & 3 for booster vaccination
S (B): Screening Visit prior to booster vaccination; X: procedure takes place, (X): procedure takes place as required at the discretion of the investigators;
B : Booster vaccination day.

Note:
Booster vaccinations are due to take place prior to the malaria season approximately one year following the third vaccination. These visits are expected in the months of April-June. If visit 31 at D421 from Table 1 coincides in the window with S (B) visit at B-30-B-1 or B+1 visit at B, these visits will be merged and procedures such as blood sampling will take place only once

 © University of Oxford, 2019

2. Background Information

2.1 Introduction

Impact of malaria and the need for a vaccine

Falciparum malaria remains one of the leading infectious causes of morbidity and mortality worldwide, predominantly affecting children and pregnant women in sub-Saharan Africa. (1) In 2017, there were an estimated 219 million cases of malaria worldwide and 435, 000 deaths. Fifteen countries in sub-Saharan Africa and India carried almost 80% of the global malaria burden. Fewer than half of the 91 malaria-affected countries and territories are on track to achieve the 40% reduction in case incidence and mortality by the 2020 milestone. It is estimated that in 2017, financing for malaria control and elimination efforts cost US$3.1 billion.(1)

The advent of artemisinin-combination therapy and increased uptake of insecticide-treated nets has resulted in significant recent reductions in mortality in many places.(2) However, emerging resistance to artemisinins, other anti-malarial drugs and insecticides (3-5)may hinder progress made towards the ultimate goal of eradication.(6)

Prevention is key, and the development of a vaccine would be invaluable in the fight against malaria. The leading vaccine candidate, RTS,S/AS01, remains unlicensed but is due to enter pilot deployment trials in Africa early in 2018, targeting licensure in African countries for general use about 2024. However, at present, no vaccine has demonstrated durable high-level efficacy. One of the primary strategic goals outlined by WHO in the Malaria Vaccine Roadmap, is the development of malaria vaccines with protective efficacy of at least 75% against clinical malaria, suitable for administration in malaria endemic areas and appropriate at-risk groups by 2030. (7)

Lifecycle of Plasmodium falciparum

The lifecycle of *P. falciparum* is complex with stages in both human and mosquito hosts (Figure 1). The bite of an infected female Anopheles mosquito transmits malaria sporozoites to the human host. These travel via the bloodstream to the liver and invade hepatocytes. Here, during the liver stage, they mature into schizonts which rupture and release merozoites over a period of 6 to 7 days. Malaria parasites are not present or detectable in the blood stream during the liver stage. The hepatocytes then rupture, releasing a large number of merozoites into the bloodstream (- the blood stage of infection). Merozoites invade erythrocytes, where they multiply and after 2 days cause the erythrocyte to rupture, releasing progeny merozoites that in turn invade new erythrocytes. A small percentage of merozoites differentiate into gametocytes: either male (microgametocytes)

 © University of Oxford, 2019

or female (macrogametocytes). These are ingested by an Anopheles mosquito, and while in the mosquito's stomach, the microgametes penetrate the macrogametes to create a zygote. The zygote matures, invades the midgut wall of the mosquito and develops into an oocyst. This grows, ruptures and releases sporozoites which migrate to the mosquito's salivary glands. These are then injected into the human host when the mosquito feeds, perpetuating the malaria life cycle.

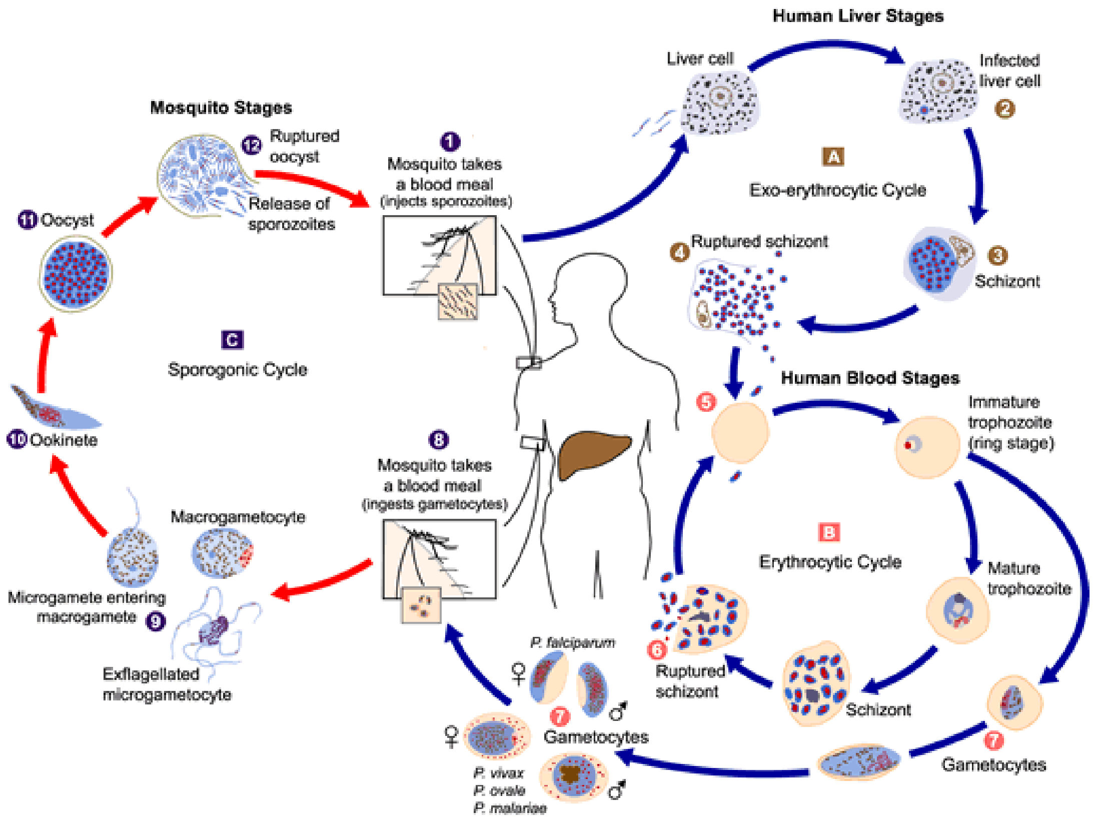

Figure 1 *Lifecycle of Plasmodium falciparum*

Progress towards a P. falciparum vaccine

Recently, there have been significant advances; the leading vaccine candidate RTS,S/AS01 has been tested in a Phase III study in African infants that completed recently. RTS,S is based on the major malaria sporozoite surface protein: the circumsporozoite (CS) protein.

The final results of a Phase III efficacy trial of the RTS,S/AS01 vaccine, in 7 sub-Saharan African countries, were published in 2015. Overall vaccine efficacy for children aged 5-17 months was 36.3% for those who were given RTS,S/AS01 at 0,1, 2 and 20 months, and 28% for those given the vaccine at 0, 1 and 2 months. For younger infants aged 6-12 weeks, it was 26% and 18% respectively.(8) Highest efficacy was noted with 4 doses but unfortunately, these are not time points in the current Expanded Programme on Immunisation (EPI) for infants , which has been responsible for the much improved vaccination coverage in Africa. This will complicate implementation of the vaccine.(9)

 © University of Oxford, 2019

Potential explanations for the reduced immune response in 6-12 week old infants include: 1) the infant's immature immune system;(10) 2) the co-administration of RTS,S with other childhood vaccines (DTPw-HepB/Hib and oral poliovirus vaccines); 3) the absence of priming with hepatitis B vaccine or with *P. falciparum* infection; and 4) maternal antibodies.(11) It is also possible that the excess of Hepatitis B surface antigen present in the formulation interferes with the induction of a potent immune response to circumsporozoite protein.

A further issue emerged on analysis of the entire RTS,S/AS01 phase III trial dataset with an unexplained increase in overall mortality of about 91% observed in female vaccinees. (12) This contributed to the decision to undertake large scale "implementation" trials along with safety assessments in many hundreds of thousands of vaccinees in three African countries. These are scheduled to start by the end of 2018.

RTS,S targets the pre-erythrocytic circumsporozoite (CS) protein, which is the major functional protein that plays a key role in sporozoite development and hepatocyte invasion.(13) 80% of the molecules in each RTS,S particle are hepatitis B surface antigen, and only 20% are fusion proteins of the malaria circumsporozoite protein moiety fused to hepatitis B suface antigen. R21, to be tested in this trial, is a protein particle that lacks the excess of HBsAg in RTS,S and has been developed at the University of Oxford. Indeed R21 comprises only fusion protein moieties, i.e. as 100% of its molecules, in contrast to RTS,S which comprises 20% of these with the remaining 80% being HBsAg molecules (Figure 2).

Pre-erythrocytic stage as a vaccine target

The pre-erythrocytic stage of *P. falciparum* infection presents an attractive target for an efficacious human vaccine, as sufficient reduction in the number of viable merozoites reaching the blood from the liver will prevent parasitisation of red blood cells and initiation of the blood stage of infection. Anti-CS antibodies can target sporozoites, facilitating destruction of sporozoites prior to hepatocyte invasion. As sporozoites travel from the skin to the liver within minutes, it may be difficult for a vaccine to achieve complete protection against *P. falciparum* based solely on antibodies to sporozoites. The liver stage of infection provides a longer window of opportunity for cell mediated immunity to recognize and destroy infected hepatocytes. Research suggests that, in isolation the RTS,S vaccine targeting the pre-erythrocytic stage antigen, CS, and vaccines targeting ME-TRAP do not delay the initial emergence of parasites into the blood, nor the rate of parasite multiplication in the blood, but rather reduce the size of this initial inoculum.(14) A delay to patent blood stage infection in persons receiving these vaccines reflects a reduced liver-to-blood inoculum. The efficacy of these pre-erythrocytic vaccine strategies can be assessed experimentally by subjecting volunteers to inoculation with *P. falciparum* sporozoites by the bite of infected mosquitoes. Complete protection against blood-stage infection, or a delay in the time to patent blood stage infection in vaccinees compared to controls, reflect vaccine efficacy.

There are a number of factors that support the selection of circumsporozoite protein as a potential target for a malaria vaccine candidate. This protein is expressed on the sporozoite surface (15) and to a lesser degree on hepatic schizonts and plays a pivotal role in alignment towards and sporozoite invasion of hepatocytes.(13, 16) In vitro, antibodies directed against B cell epitopes derived from this protein can inhibit the infectivity of sporozoites to liver cells.(17) In murine models, passive transfer of antibodies to the immunodominant B cell repeat epitope of the CS protein as well as active immunisation with constructs containing this epitope, confer protection against sporozoite challenge.(18, 19) Furthermore, it has been shown that adoptive transfer of $CD8^+$ CTL or $CD4^+$ T cell

 © University of Oxford, 2019

clones specific for epitopes on the CS protein can provide protection against a sporozoite challenge.(20, 21) Finally the leading malaria vaccine candidate, RTS,S, induces partial efficacy by inducing antibodies against the central repeat (NANP) of the circumsporozoite protein.

The influence of the gut microbiome on the infant immune response to vaccination

Factors that can influence the infant gut microbiome include gestational age, route of birth, diet, and maternal diet and weight. (22-24)This leads to considerable variation in the microbiome between individuals. Antibiotic exposure can significantly change the composition of the microbiome, leading to dysbiosis. (25, 26) A single course of intrapartum antibiotics has been shown to affect the infant microbiome until at least 3 months of age. (27)

In mice, the microbiome has been shown to play a significant role in driving early postnatal innate immune development, (28) and in shaping adaptive immune responses, such as the regulation of T helper 17 and regulatory T cell responses.(29)

Recently, there have been some clinical studies that have suggested the possible role of the microbiome in determining optimal vaccine responses in humans. In a study in Bangladeshi infants, bacterial species were identified that have been associated with vaccine-specific IgG and T cell proliferation responses to oral polio, BCG, tetanus toxoid and HBV vaccines. (30) The abundance of certain bacterial species positively or negatively correlated with vaccine response. This was similarly noted in Ghana when looking at rotavirus vaccine responses. (31)

Further investigation into humoral and cell-mediated responses to infant vaccinations is warranted to see if they are affected by the infant microbiome. There are currently few studies documenting this and whether there is potential to modify vaccine responses according to particular species of the microbiome. There have been no studies investigating associations of species and vaccine responses with intramuscular vaccinations and this may be key to understanding the response to this malaria vaccine candidate, R21 adjuvanted with Matrix-M, or how it can be improved. The gut microbiome can be analysed with a rectal swab or faecal sample.

3.2 Investigational products

3.2.1 R21 vaccine development

R21 has been developed at the Jenner Institute, University of Oxford. It is produced by using recombinant HBsAg particles expressing the central repeat and the C-terminus of the circumsporozoite protein (CSP). R21 was originally manufactured to GMP at The Clinic Biomanufacturing Facility (CBF) in Oxford in *Pichia pastoris* and is now being manufactured at the Serum Institute of India (SII) in *Hansenula polymorpha.*

In Oxford the four amino acid EPEA sequence (the C-tag) was used as a very short extension of R21 (called R21c) solely to facilitate purification at Oxford. Since then there has been a slight modification to the structure and also an up-scaled manufacturing process at the Serum Institute of India. There is now no C-tag, so that R21 does not have the four amino acid sequence, EPEA, at the C-terminus. This 4 amino acid tag was included in R21 vaccine produced in Oxford, sometimes called R21c to indicate the presence of the tag, simply to facilitate an immunoaffinity column step in the purification of the vaccine during biomanufacturing. With improvements to the manufacturing process this small tag in no longer required and it has been removed from the product undergoing further development. This new product is therefore minimally different from R21c but has not been evaluated in human clinical trials.

 © University of Oxford, 2019

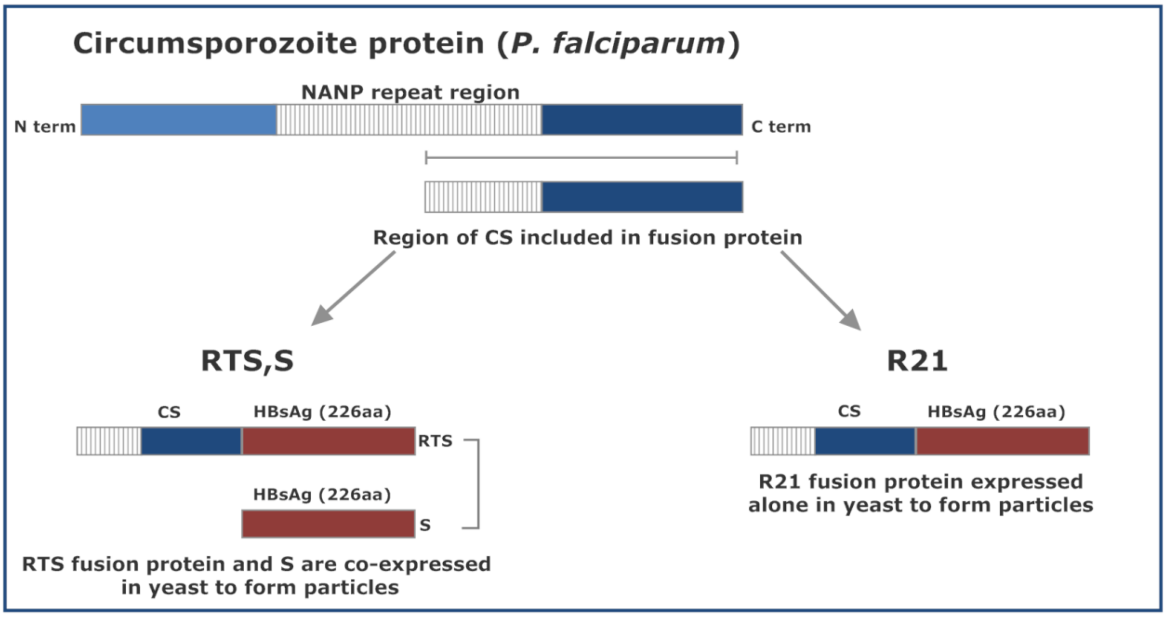

Figure 2: *Schematic diagram showing RTS,S and R21 fusion proteins. Both RTS,S and R21 include the fusion protein of hepatitis B surface antigen to the C-terminus and central repeats of the circumsporozoite (CS) protein. These repeats comprise many copies of the four amino acid sequence NANP. R21 is a virus like-particle that results for spontaneous assembly of the R21 molecules. RTS,S, expressed in a different yeast type required expression of a fourfold excess of the unfused hepatitis B surface antigen to allow it to form hybrid particles. Generation of virus-like particles by both RTS,S and R21 has been shown to be important for allowing induction of high level antibody responses.*

RTS,S/AS01 vaccine, induces very strong antibody responses to the conserved central repeat of CSP, of the order of 100 - 600 micrograms per ml, some weak mainly IL-2-secreting $CD4^+$ T cell responses and no CSP-specific $CD8^+$ T cells.(32) The most reproducible correlate of protection in clinical studies is IgG antibody titre.(32, 33) The R21 particle contains no *P. falciparum* sequences that are not present in RTS,S, which has been safely used in thousands of individuals. It is a hybrid protein of the majority of the CS protein of *P. falciparum* fused to the hepatitis B surface antigen (Figure 2). It spontaneously forms a particle in the same way as RTS,S and initial Phase I/II studies have shown that R21c is a very immunogenic particle in humans, with at least as high antibody levels generated as those induced by RTS,S, but with just 20% of the RTS,S dose (10 µg rather than 50 µg). This may be due to the fact that R21c induces predominantly CSP rather than HBsAg antibodies, (See Figure 3.2) probably because it has a higher proportion of malaria to HBsAg in its composition than RTS,S. This is made possible by expressing R21c in the better expressing yeast *Pichia pastoris*, rather than in *Saccharomyces cerevisiae*. The new R21 produced by SII, will be expressed in the yeast *Hansenula polymorpha* and not contain the C-tag.

3.2.2 R21 - pre-clinical studies

Immunogenicity

Initial pre-clinical assessment of immunogenicity was undertaken in BALB/c mice that were immunised intramuscularly with 0.5µg of R21 alone or in combination with an adjuvant (Alhydrogel or Abisco). Immune responses including antibody levels to the central NANP repeat region and antigen–specific T cell responses were measured three weeks after a 3-dose immunisation schedule (Figures 3.1 & 3.3). R21 + Abisco-100 (which is essentially the same as the Matrix-M to be used in

 © University of Oxford, 2019

this trial and made by the same company), a potent saponin-based adjuvant resulted in the greatest humoral immune response at each time point in the vaccination schedule. (Figure 3.1)

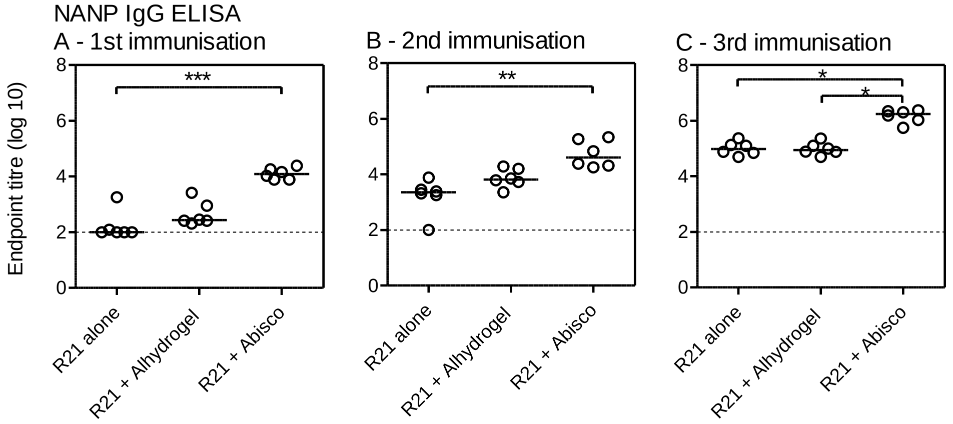

Figure 3.1*: Pre-clinical assessment of immunogenicity with 0.5µg of R21 alone or in combination with an adjuvant (Alhydrogel, Abisco).*

The responses in all groups were boosted by a third immunisation and R21 + Abisco-100 induced the highest titres of NANP specific IgG and the response for this group was significantly higher than both the R21 + Alhydrogel and R21 alone groups after the final immunisation (Figure 3.1).

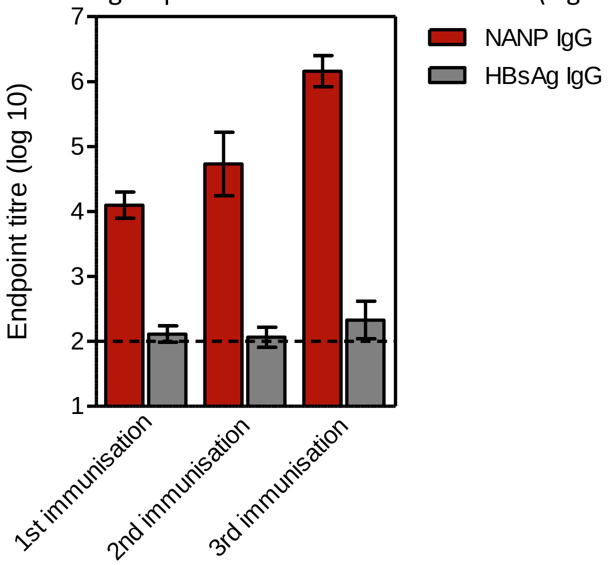

Figure 3.2: *Relative proportions of IgG to NANP and HBsAg after immunisation with R21 + Abisco-100 in BALB/c mice.*

Antibodies to hepatitis B surface antigen were measured in the same study. As expected from the composition of R21 and RTS,S antibodies to hepatitis B surface antigen were much lower with R21 than RTS,S (Figure 3.2) probably reflecting a structure in R21 where the CS sequences are likely found on the exposed surface of the virus-like particle whereas the surface of RTS,S comprises mainly hepatitis B and a minority of malaria epitopes.

CS-specific IFN-γ producing T cells measured after the third immunisation were only detected at high levels in mice immunised with R21 + Abisco-100 (Figure 3.3). R21 alone was ineffective at inducing CS-specific T cell responses on its own.

 © University of Oxford, 2019

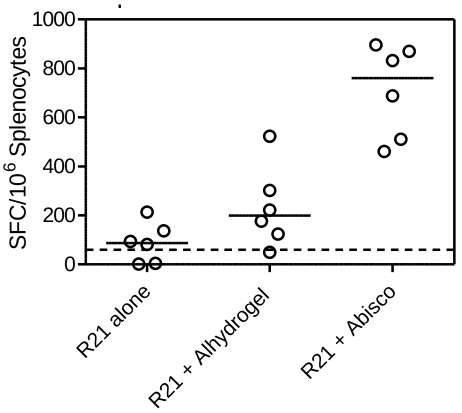

Figure 3.3: *CS-specific IFN-γ producing T cells measured after the third immunisation*

Efficacy

Sporozoite challenge (1000 sporozoites per mouse injected intravenously) using transgenic *P. berghei* parasites was performed in BALB/c mice (Figure 3.4). R21 + adjuvant was given twice, eight weeks apart and mice were challenged three weeks after the second dose. Thin blood films to detect parasitaemia were performed daily from day 5 post-challenge. Sterile protection was defined as remaining slide negative at day 14 and significant delay in development of 1% parasitaemia compared to non-immunised control mice was regarded as partial efficacy.

R21 + Abisco-100 sterileу protected 100% of the challenged mice (p=< 0.0001) and R21 + Matrix-M sterileу protected 87.5% (p=0.0002) and this was confirmed in a second independent challenge (p = < 0.0001). There was no significant difference between the groups with these two very similar adjuvants.

The durability of efficacy was assessed by undertaking sporozoite challenge in mice seven and fourteen weeks after immunisation. Efficacy was well maintained at seven weeks post immunisation with 75% of mice sterilely protected (6/8) and this was not significantly different when compared to efficacy at three weeks post immunisation (p=0.4468, by Log-rank (Mantel-Cox) Test).

This reduction in protective efficacy can however be boosted to 100% if mice are challenged once (three weeks post immunisation) within the 14 weeks. Therefore, efficacy after vaccination and one sporozoite infection is very durable and 100% sterile efficacy is maintained for at least 14 weeks.

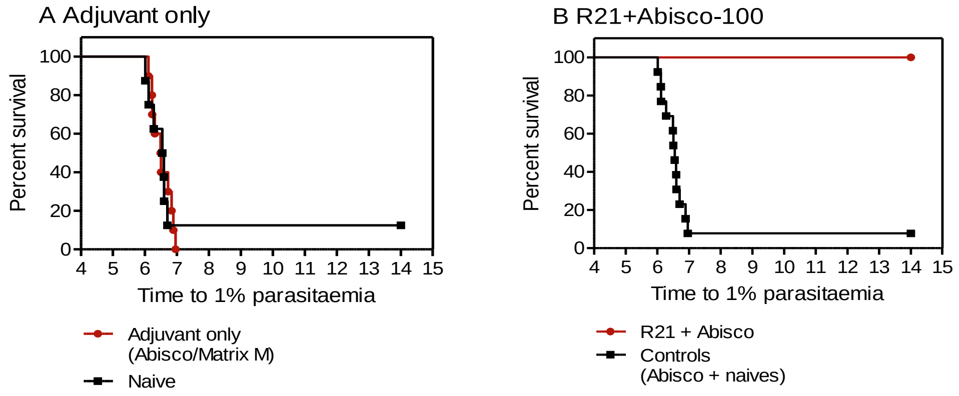

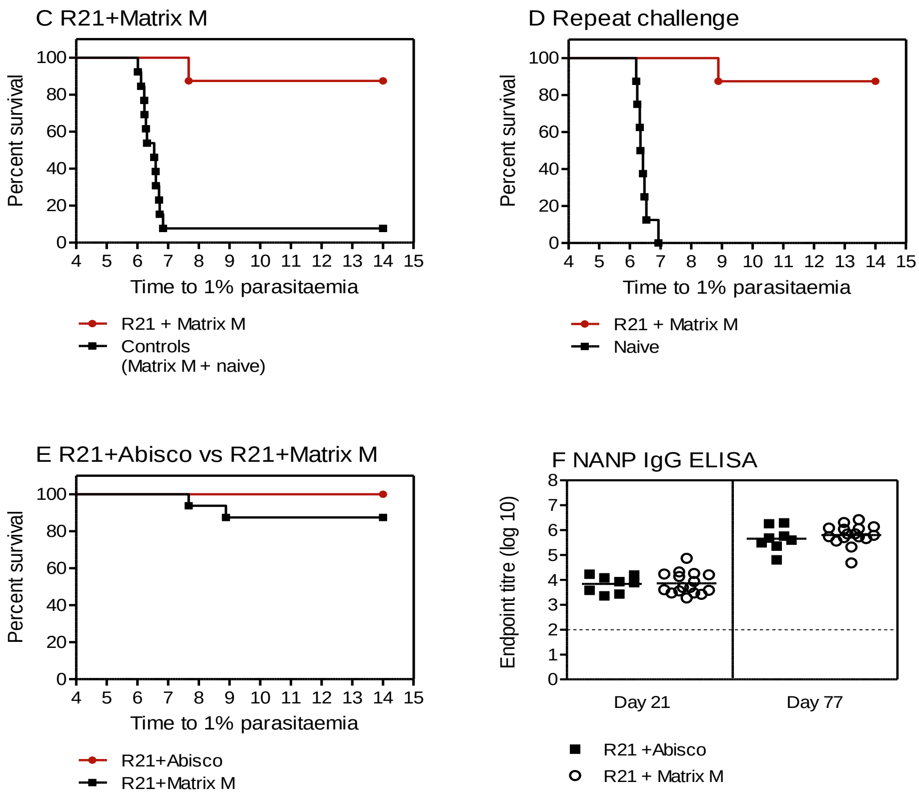

Figure 3.4 (A-F): *Protective efficacy elicited by saponin based ISCOM adjuvants with R21 in a transgenic sporozoite model. BALB/c mice were immunised i.m. with 0.5µg R21 + adjuvant (Abisco-100 or Matrix M), twice eight weeks apart (n=8/group). Mice were challenged three weeks after the final vaccination by i.v. injection of 1000 sporozoites (P. berghei transgenic for P. falciparum CSP) along with eight naïve mice. Two groups of adjuvant control mice (n=5/group) were also challenged three weeks after receiving two shots of adjuvant (Abisco-100 or Matrix M) i.m., eight weeks apart. Blood stage parasitemia was monitored from day 5 after challenge by thin-film blood smear, and time to 1% parasitemia was calculated using linear regression. The results are presented in the Kaplan-Meier survival graphs and survival curves were compared by Log-rank (Mantel-Cox) Test.* ***(A)*** *Adjuvant control = no significant difference,* ***(B)*** *R21 + Abisco-100 p<0.0001,* ***(C)*** *R21 + Matrix M p=0.0002,* ***(D)*** *R21 + Matrix M repeat p=<0.0001,* ***(E)*** *R21 + Abisco vs R21 + Matrix M = no significant difference. Blood was taken three weeks after each vaccination (Day 21 and Day 77) for immunology and NANP specific IgG was assayed by ELISA* ***(F)****, group mean responses shown and dotted line indicates the limit of detection.*

3.2.3 R21c- Phase I clinical trials

VAC 053

This was a Phase I study to assess the safety and immunogenicity of R21c, administered with and without Matrix-M in healthy UK volunteers. The study design is shown below.

	**Day 0**	**Day 28**	**Day 56**
**Group 1 (n=10)**	10µgR21c/50µMM	10µgR21c/50µMM	10µgR21c/50µMM
**Group 2 (n=4)**	50µR21c	50µR21c	50µR21c
**Group 3 (n=10)**	50µgR21c/50µMM	50µgR21c/50µMM	50µgR21c/50µMM

 © University of Oxford, 2019

**Group 4 (n=10)**	2µgR21c/50µMM	2µgR21c/50µMM	2µgR21c/50µMM

Table 3: ***VAC 053 study groups.***

31 volunteers were enrolled in the study (one Group 1 volunteer withdrew and was replaced). Most adverse events were mild in nature and all the interim clinical safety reviews were satisfactory. There have been no SAEs or SUSARS. No group holding or individual stopping rules have been activated. Pain at the vaccine site was the most frequently reported AE. There have only been 4 severe AEs reported - all in Group 3; these were short-lived and resolved within 24-48 hours.

Durable antibody responses were observed at 6 months after the final vaccination and the 10µg dose elicited significantly higher titres compared to the 50µg dose at 6 months. Overall, R21c adjuvanted with Matrix-M was safe and well tolerated.

This trial was the first administration of R21c in humans and immunogenicity profiles observed were encouraging (Figure 4A). Antibody levels observed are comparable to our previous experience with RTS,S/AS01 in the VAC055 trial with 50µg RTS,S. (Figure 4B) (N Venkatraman, in preparation).

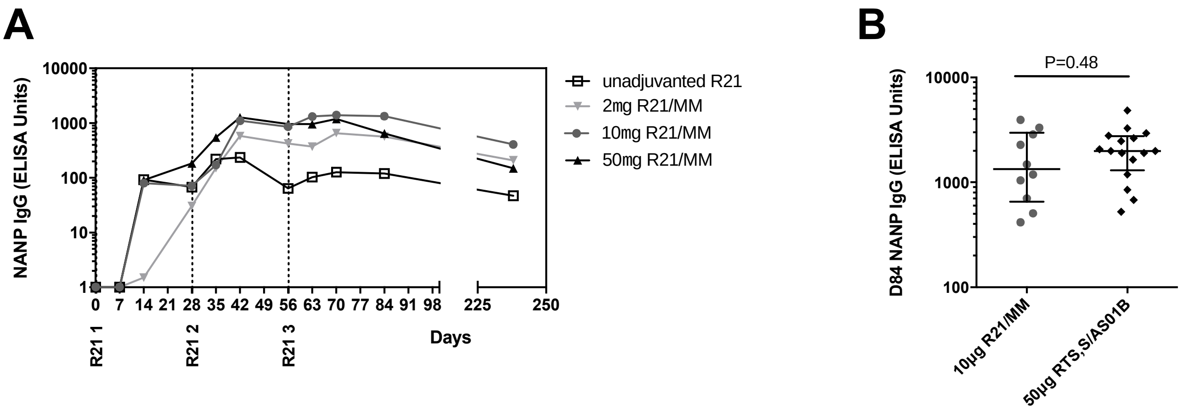

Figure 4: *Mean IgG antibody responses to the NANP repeat region of the pre-erythrocytic circumsporozoite protein.*

VAC 056

This was a Phase I study to assess the safety and immunogenicity of R21c at two different doses administered with the saponin-based adjuvant $AS01_B$. The study groups are shown below in Table 4.

	Day 0	Day 28	Day 56
**Group 1 (n=10)**	10µgR21c/$AS01_B$	10µgR21c/$AS01_B$	10µgR21c/$AS01_B$
**Group 2 (n=10)**	50µgR21c/$AS01_B$	50µgR21c/$AS01_B$	50µgR21c/$AS01_B$

Table 4: ***VAC 056 study groups.***

 © University of Oxford, 2019

20 volunteers were enrolled and vaccinated. The majority of adverse events were self-limiting and mild in nature. There have been no SAEs or SUSARS. No group holding or individual stopping rules were activated. Vaccine site pain was the most common local adverse event and was predominantly mild in severity. There was no significant difference in the adverse event profile of the 10µg and 50µg R21c groups. There were only 3 severe AEs reported – all in Group 2; these were short-lived and resolved within 24-48 hours.

R21c administered at both doses of 10µg and 50µg with the adjuvant $AS01_B$ elicited similar humoral immune responses which were maintained 6 months post final vaccination with no differences in durability.

In conclusion, this Phase I clinical trial showed that R21c administered with $AS01_B$ was safe and well tolerated, with comparable humoral immune responses to the leading malaria vaccine candidate, RTS,S/AS01, at both doses of 10 and 50µg. (See Figure 5) (N Venkatraman, in preparation)

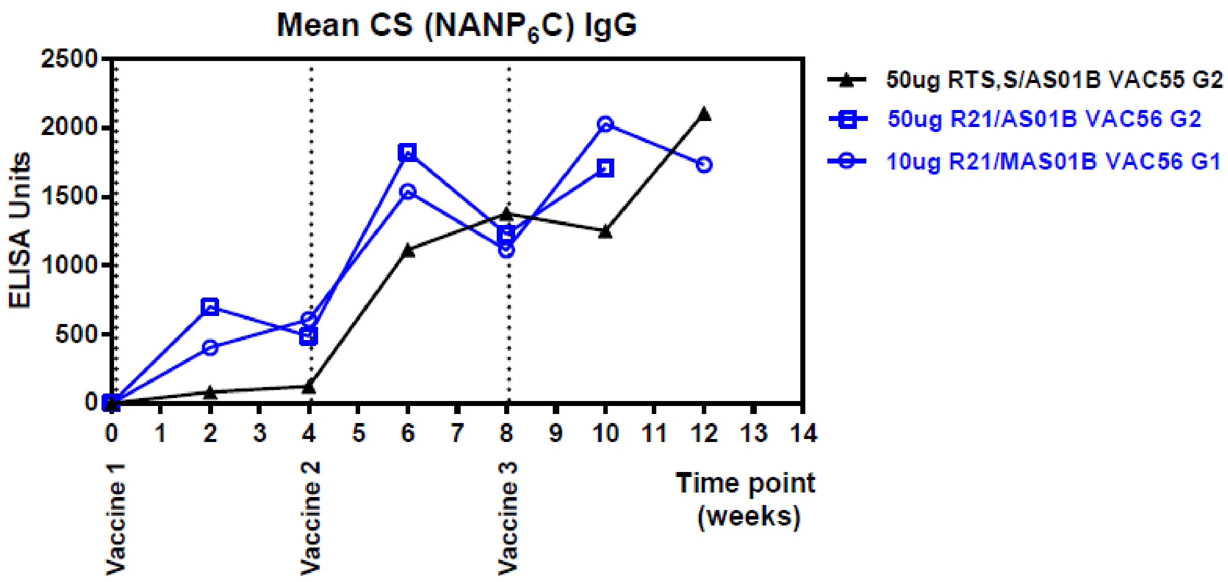

Figure 5: *Mean IgG antibody responses to the pre-erythrocytic circumsporozoite protein*

VAC 065

This was a phase I/IIa sporozoite challenge study to assess the safety and protective efficacy of adjuvanted R21 at different doses and the combination malaria vaccine candidate regimen of adjuvanted R21 + ChAd63 and MVA encoding ME-TRAP.

66 volunteers were enrolled in the trial. Challenge A was enrolled, vaccinated and followed-up from November 2016-February 2017. CHMI took place 30-31st January 2017. Challenge B was enrolled, vaccinated and followed-up from July 2017-October 2017. CHMI took place 17th-18th September 2017, eight and a half months after the last vaccination The volunteers in Group 6 were recruited from a previous R21c trial, VAC053. (See Table 5 and Figure 6).

**Week**	**0**	**1**	**4**	**8**	**9**	**11-12**	**32-64**	**52-104**

 © University of Oxford, 2019

**Group 1 n=12**	10µg R21c / 50µg Matrix-M		10µg R21c / 50µg Matrix-M	10µg R21c / 50µg Matrix-M		CHMI	repeat CHMI of sterilely protected volunteers	
**Group 2 n=12**	50µg R21c / 50µg Matrix-M		50µg R21c / 50µg Matrix-M	10µg R21c / 50µg Matrix-M		CHMI	repeat CHMI of sterilely protected volunteers	
**Group 3 n=12**	10µg R21c / 50µg Matrix-M	ChAd63 ME-TRAP	10µg R21c / 50µg Matrix-M	10µg R21c / 50µg Matrix-M	MVA ME-TRAP	CHMI	repeat CHMI of sterilely protected volunteers	
**Group 4a^ n=6**						CHMI (controls)		
**Group 5 n=12**	10µg R21c / 50µg Matrix-M		10µg R21c / 50µg Matrix-M	2µg R21c / 50µg Matrix-M		CHMI	repeat CHMI of sterilely protected volunteers	
**Group 6* n=1-8**	10µg R21c / 50µg Matrix-M		10µg R21c/ 50µg Matrix-M	10µg R21c / 50µg Matrix-M				CHMI
**Group 7 n = 8**	10µg R21c / 50µg Matrix-M		10µg R21c / 50µg Matrix-M			Week 7-8 CHMI		
**Group 4b[%] n=4-6**						CHMI (controls)		

Table 5: *Vaccination groups.* ^ Group 4a were infectivity controls when groups 1-3 had initial CHMI (challenge A)

% Group 4b were infectivity controls when group 5 and 6 had initial CHMI and sterilely protected volunteers in groups 1-2 had repeat CHMI (challenge B)

* Group 6 received their vaccination during their enrolment into VAC053

 © University of Oxford, 2019

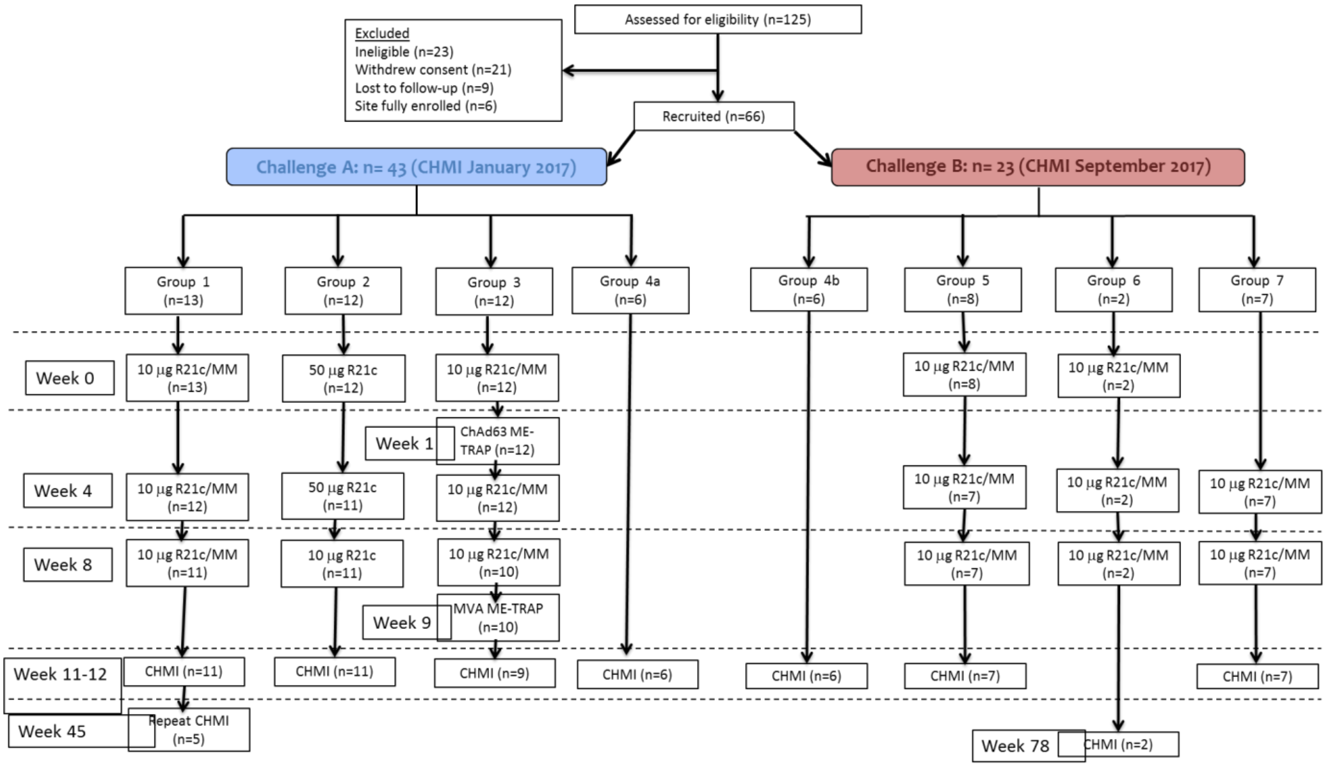

Figure 6: VAC065 flow chart of study design and volunteer recruitment.

Abbreviations: MM, Matrix-M. Abbreviations: ChAd63, chimpanzee adenovirus serotype 63; ME-TRAP, multiple epitope string fused to the thrombospondin-related adhesion protein; MVA, modified vaccinia Ankara; ChAd63 ME-TRAP was administered at 5 x 10^{10} viral particles and MVA ME-TRAP was administered at 2 x 10^{8} plaque forming units. A total of 125 volunteers were screened and 66 were enrolled in total.

Safety

R21c in combination with Matrix M is safe and well tolerated with adverse events being predominantly mild in nature and self-limiting. Vaccine injection site pain was the most common local adverse event and was predominantly mild in severity.

10/10/10µg R21c with MM had a favourable reactogenicity profile compared to RTS,S/$AS01_{B.}$ Comparison was made with data from a previous clinical trial conducted in our centre where volunteers received three doses of RTS,S/$AS01_{B.}$ The adverse event profile was statistically significantly improved with 10mg R21/MM after each dose in comparison to 50mg RTS,S/$AS01_{B}$ (Vaccination 1- p<0.0001, Vaccination 2- p<0.0001 and Vaccination 3- p=0.005; chi-squared test comparing adverse event rates stratified by grade). There was also a considerably higher percentage of moderate and severe adverse events reported by volunteers receiving RTS,S/$AS01_{B.}$

 © University of Oxford, 2019

Immunogenicity

There were no statistically significant differences between the IgG responses to NANP between the R21c/MM groups and there was a broad range in magnitude of antibody responses in those protected against CHMI. This suggests that the quality of the initially induced humoral response was relevant to efficacy in addition to the magnitude of the response. Additionally, antibody responses were well maintained at 8.5 months post-vaccination.

Efficacy

High level efficacy (82%) was observed with 10/10/10µg R21c with Matrix- M; amongst the highest efficacy reported for any malaria vaccine (See Figure 7.) The addition of viral-vectored vaccines to this or a fractional third dose of R21c did not result in improved efficacy. Durable vaccine efficacy of 60% was observed at 8.5 months. Efficacy of 57% was observed with 10/10 g R21c/MM, which is apparently the highest level of CHMI efficacy reported for a two-dose malaria vaccine regime. (See Figure 8)

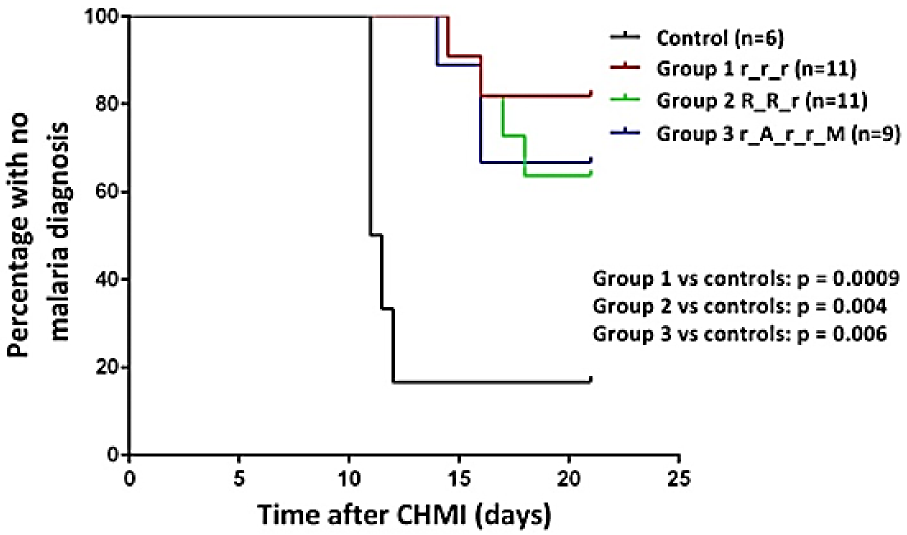

Figure 7: Challenge A efficacy. *Efficacy of Group 1 low dose 10µg, 10 µg, 10 µg regime = 82% sterile protection. (Corrected VE = 78% for Group 1 volunteers allowing for 1/6 controls not infected). Group 2- R21c 50 µg, 50 µg, 10 µg with Matrix-M 50 µg. Group 3- R21c 10 µg, 10 µg, 10 µg with Matrix-M 50 µg and ChAd 63 MVA ME-TRAP*

 © University of Oxford, 2019

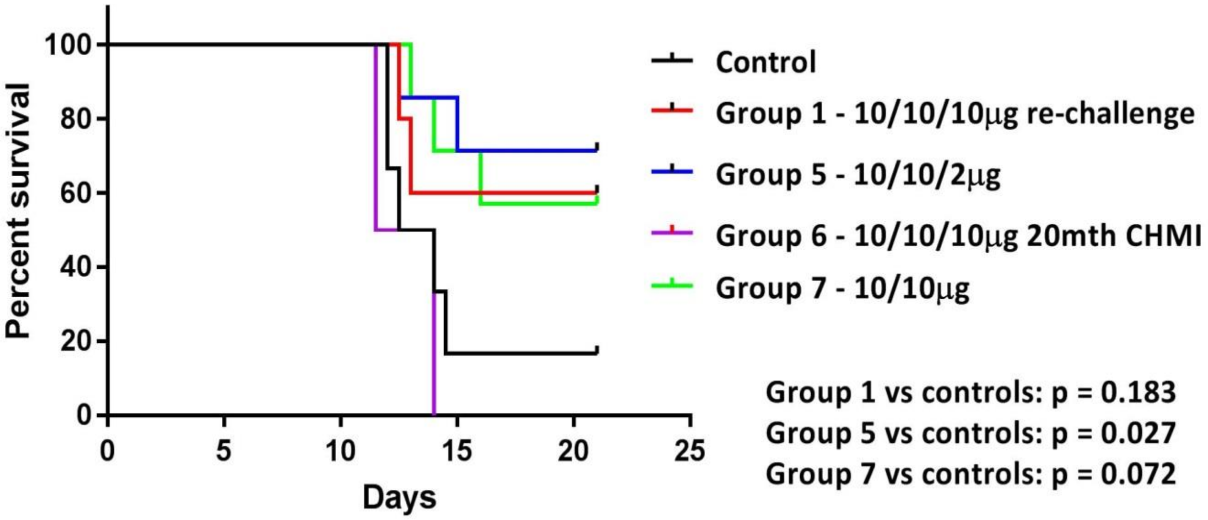

Figure 8: Challenge B efficacy. *Efficacy of two dose 10 µg, 10 µg regime at 3-4 weeks = 57%. (Group 7). Durable efficacy of three dose 10 µg regime at 8.5 months = 60% (Group 1)*

In conclusion, this Phase I/II clinical trial showed that R21c adjuvanted with Matrix-M was safe and well tolerated in UK subjects. The vaccine regime of 10/10/10µg R21 with MM showed good efficacy with durable efficacy and antibody response observed at 8.5 months. The two-dose regime also displayed high level efficacy. (N Venkatraman, in preparation)

VAC060

This was a Phase Ib study of R21c with Matrix-M conducted in a total of 12 healthy Burkinabe adults aged between 18 and 45 years at the Centre National de Recherche et Formation sur le Paludisme (CNRFP) research unit, Banfora in Burkina Faso. The study protocol for the Phase Ib study was approved by the Burkina Faso regulatory authorities, The Burkina Faso Ministry of Health, and Institutional Bioethics Committee, National Malaria Research and training Centre (CIB/CNRFP), and Oxford Tropical Research Ethics Committee (OXTREC Reference: 36-15). The trial was registered with ClinicalTrials.gov (Ref: NCT02925403). An independent Data Safety and Monitoring Board (DSMB) provided oversight. The trial was monitored by an external organization (Margan Clinical Research Organization).

The study was a randomised, controlled trial assessing the 10µg dose adjuvanted with Matrix-M in Burkinabe adults. Vaccinations only commenced after a satisfactory DSMB review of the interim safety report for the 10µg and 50µg dose of R21c given to volunteers in the UK Phase Ia study. Volunteers were randomised to receive R21c/Matrix-M or a normal saline placebo. There were 8 R21 Matrix-M vaccinees and 4 controls. Simple randomisation into the study groups was done by an independent statistician based at the University of Oxford. A randomisation code list was generated and its use guided by a clear Standard Operating Procedure (SOP). Opaque sealed envelopes were employed to maintain allocation concealment and the laboratory scientists were blinded to vaccine allocation until the end of the study.

Safety

 © University of Oxford, 2019

The vaccine was well tolerated in Burkinabe adults. Local and systemic adverse events to 10µg R21 adjuvanted with MM in Burkinabe adults are shown in Figure 9. Local adverse events were markedly reduced compared with the UK adults (n=40 events in UK cohort vs. n=7 in Burkinabe participants at the same dose). The majority of local adverse events were mild in nature and overall reactogenicity was significantly reduced compared to UK volunteers receiving the same dose (p=<0.0001, Pearson's chi-squared test for trend). None of the volunteers reported any severe adverse events. Vaccine site pain was again the most commonly reported local adverse event. Both systemic and local reactogenicity was minimal and there were no episodes of fever associated with vaccination in the Burkinabe cohort.

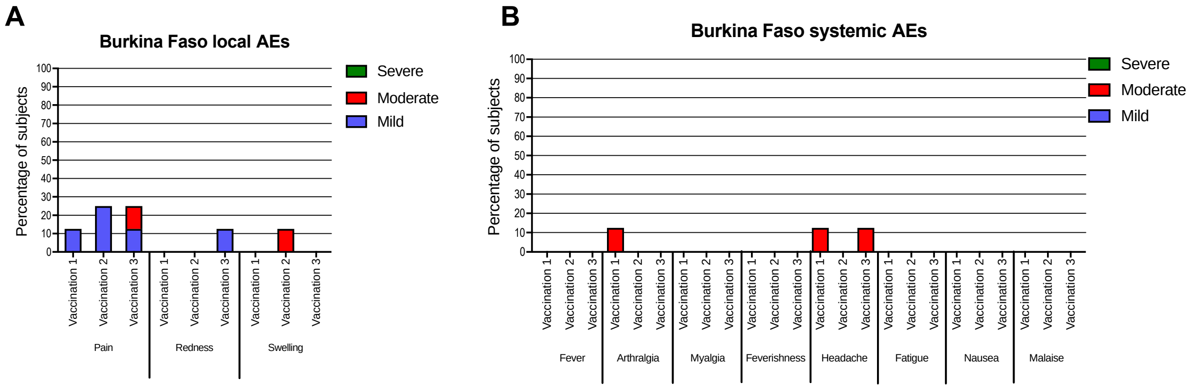

Figure 9. Comparison of local (A) and systemic (B) solicited adverse events (AE) reported by Burkinabe volunteers during the first seven days related to their first, second and third vaccination with a dose of 10µg R21/MM. *Only the highest intensity of each AE per subject is listed. Data are combined for all AEs for all volunteers receiving the same vaccine at the stated time point.*

Immunogenicity

In the Burkinabe cohort, prevaccination titres to CSP were higher than in UK participants due to malaria exposure. Responses to vaccination did not differ significantly between the two cohorts after the first two vaccinations, however the third dose of R21c failed to significantly boost responses in these adult Burkinabe subjects (Figure 10A). Initial assessments of functional activity of the vaccine-induced antibodies in these subjects using an invasion of sporozoites inhibition (ISI) assay show at least a high activity in the Burkinabe subjects as in Oxford vaccines after the third dose (Figure 10B).

 © University of Oxford, 2019

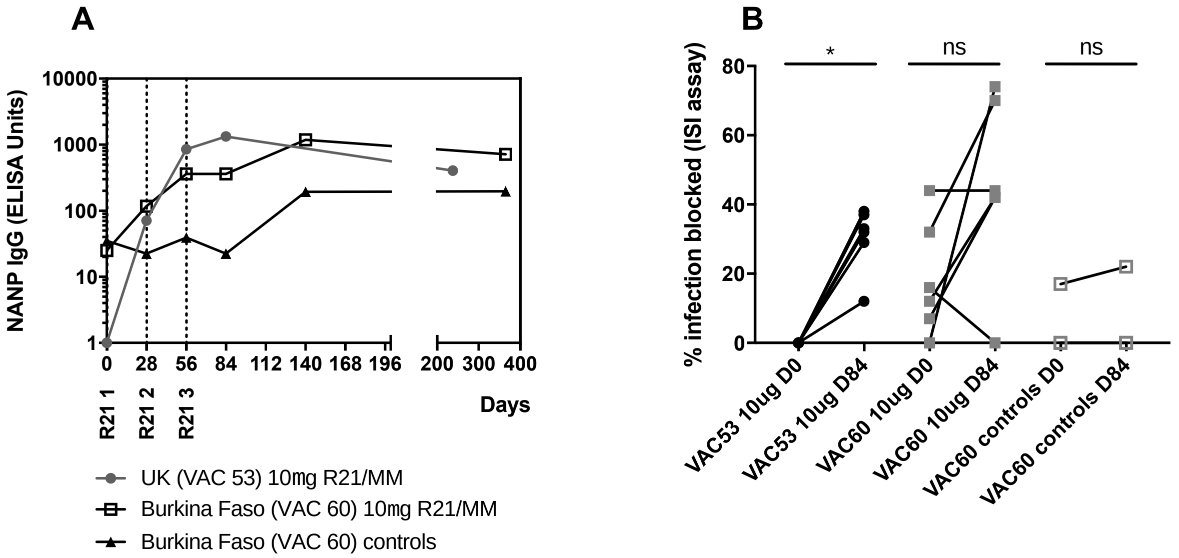

Figure 10. Comparison of antibody responses to CSP in UK and Burkina Faso. A ELISA responses to the NANP repeat region of CSP before and after vaccination with R21 or in controls. B. Inhibition of sporozoite invasion blocked by serum from UK and Burkinabe populations *, p<0.05; ns, not statistically significant.

In summary, Phase I clinical trial data with R21c adjuvanted with Matrix-M was safe and well tolerated in both UK and African subjects with a better somewhat better safety profile in Burkina Faso, with good antibody immunogenicity observed in both populations.

VAC 073

This is a phase Ib, age de-escalation, dose-escalation study to evaluate the safety and immunogenicity of R21 adjuvanted with Matrix-M in a total of 93 adults, young children and infants in Kilifi, Kenya. At the time of writing, this Phase I trial is due to start immunisations in April 2019, so that it is likely that prior to children being vaccinated for this Phase I/IIb study in Burkina Faso, age de-escalation to young children and infants will have occurred and safety data will be available to the investigators for this Nanoro trial. There will be overlap in membership of the DSMB for this Kenyan trial and the proposed Nanoro trial and safety information from the Kenyan trial will be made available to the DSMB for the proposed Nanoro trial.

3.2.4 Matrix-M

Matrix-M (MM) is a 40nm-sized complex containing the adjuvant-active saponin *Quillaja saponaria*, phospholipid and cholesterol. Quillaja saponins are triterpene glycoside substances derived from the bark of the tree *Quillaja saponaria*. The molecular weights of the different saponins range from 1800 - 2000 Da. In water, saponin in concentrations of 200-500 ppm exist as monomers; at higher concentrations they aggregate as micelles, with a molecular weight of approximately 100,000 Da. Saponins are surface-active compounds with a variety of applications including in agriculture, feed, food and beverage, mining, and veterinary vaccines, and over the last 20 years have been increasingly investigated in human vaccine clinical trials. For example the recently licensed shingles vaccine from GSK contains the Saponin QS21 as part of the AS01 adjuvant. In aqueous solution, saponins are excellent adjuvants and are widely used in commercial veterinary vaccines, e.g., vaccines against foot-and-mouth disease, bovine mastitis, feline leukemia and equine influenza.

 © University of Oxford, 2019

Matrix-M is being developed by Novavax as an adjuvant for their new influenza vaccine and a licensure application to the FDA for this influenza vaccine adjuvanted with Matrix-M is planned.

3.2.5 Matrix-M pre-clinical studies

In animal studies, Matrix-M has been shown to perform better than most other adjuvants, inducing a multifaceted response including antibody production, T cell responses and recruitment of innate immune cells into draining lymph nodes.(34, 35) Mixed with a virosomal H9N2 avian influenza vaccine, Matrix-M induced enhanced antigen-specific humoral and CD8+ T cell responses.(36) Matrix-M administered with an intramuscular H5N1 virosomal influenza vaccine induced a strong immediate and long-term humoral and cellular immune response and showed a dose-sparing potential.(37)

In pre-clinical studies, R21 adjuvanted with both Matrix-M (MM) and MF59 has demonstrated good antibody responses.(38) In addition, there was no interference with induction of antibodies or T cells when R21/MF59 was combined with viral vectors.(38) BALB/c mice immunised with 2 doses of R21/MM showed excellent efficacy (91.3% sterile protection) against transgenic malaria parasite challenge.(38) Combining protein and viral-vectored vaccines in murine malaria models has also previously been shown to have a synergistic effect resulting in much higher sterile efficacy (90%) than either vaccine individually.(39)

3.2.6 Matrix-M-effect in humans

More than 1800 human subjects have received Matrix-M, as an adjuvant for vaccines against several diseases including malaria, influenza and ebolavirus disease. Collectively, the clinical data with Matrix-M at doses up to 75µg shows that vaccines containing the adjuvant have reversible acute reactogenicity (i.e. self-limiting fever and pain in some subjects) but are generally well-tolerated and demonstrate an acceptably safety profile. Matrix-M adjuvanted vaccine formulations have also demonstrated a clear immunogenicity benefit, with documented evidence of antigen dose-sparing.(40)

 © University of Oxford, 2019

3. Rationale

Vaccine Development Strategy

R21 has been developed at the Jenner Institute, University of Oxford. It is produced by using recombinant HBsAg particles expressing the central repeat and the C-terminus of the circumsporozoite protein (CSP). R21 was originally GMP manufactured at The ClinicBiomanufacturing Facility (CBF) in Oxford in *Pichia pastoris* and is now being manufactured at the Serum Institute of India (SII) in Pune, India, in *Hansenula polymorpha.* This is a biosimilar protein particle to RTS,S which also targets the pre-erythrocytic circumsporozoite protein, the major functional protein in sporozoite development and hepatocyte invasion. R21 lacks the excess of HBsAg in RTS,S (See Figure 2) and has been shown to be highly immunogenic (> 10^5 ELISA units after two immunisations) and have at least comparable immunogenicity and similar high level efficacy to RTS,S in animal studies.(41)

To date, safety and immunogenicity observed in VAC 065 is very promising and antibody levels observed are comparable to previous studies done in Oxford with the leading malaria vaccine candidate, RTS,S. Furthermore, good efficacy at one month with durable efficacy and well maintained antibody responses were observed at 8.5 months after the third vaccine dose. (N Venkatraman et al, in preparation)

Matrix-M is an attractive adjuvant, as it, and other matrix formulations of Quillaja saponins, show good safety profiles, and the ability to enhance both cellular and humoral immune responses to a range of vaccines. Preclinical data presented in Section 3.4 demonstrate the potential for Matrix-M to enhance the immunogenicity of R21.

In view of these encouraging results, it has been deemed important to progress clinical evaluation of this new malaria vaccine in populations that might benefit most from it, particularly African infants and children. In addition, it is preferable to use a product that does not require additional sequences present simply to facilitate biomanufacturing. Therefore, the additional 4 amino acids E-P-E-A, comprising the C-tag sequence, have been removed from the new R21 vaccine and the product is now manufactured at a much larger scale at the Serum Institute of India, a major large scale vaccine supplier. No functional effect is expected from the removal of these four amino acids.

Here, we propose to test safety, immunogenicity and efficacy of the new R21 without the C-tag, and with two different doses of Matrix-M: 25 micrograms and 50 micrograms (50µg). In adults, the 50 µg dose is used but in 5 – 17 month olds the 25 µg dose may be sufficient. In parallel, trials of R21 adjuvanted with Matrix-M will be taking place in Kilifi, Kenya to assess safety and immunogenicity in Africa in a range of age groups and in adults in Oxford but in different dosing schedules. It is possible that R21 adjuvanted with Matrix-M will provide a more efficacious and less expensive vaccine compared to the current leading RTS,S candidate. R21 adjuvanted with Matrix-M should be less expensive than RTS,S/AS01 for at least three reasons: a lower antigen dose by about 5-fold, a simpler less expensive adjuvant, and future market supply by a very large-scale manufacturer that is a major UNICEF supplier of suitably-priced vaccines for Africa. We will test the R21/MM vaccine with a half-dose (25 µg) of adjuvant and a full-dose of adjuvant (50 µg) as if immunogenicity and efficacy is similar, this would lead to dose sparing of the adjuvant, which would reduce overall costs.

 © University of Oxford, 2019

During the efficacy trials assessing RTS,S/$AS01_B$, increased efficacy was noted in this age group (5-17 months) when a fourth booster vaccine dose was given at month 20. Efficacy of 28.3% was noted in 5-17 month olds who had received 3 vaccine doses whereas in those who received the fourth dose, efficacy was 36.3% after 48 months of follow-up. We propose to not only test the efficacy following a fourth dose, but to give this booster vaccine specifically before the malaria season commences (April-June) in Burkina Faso, in order to boost the immunogenicity prior to the higher prevalence of malaria. If for any logistic reason the booster doses cannot be completed in some volunteers by the end of June, they can be boosted in early July.

The dose and nature (i.e. rabies vs R21/MM) of this booster vaccination will be the same, for each individual, as the first 3 vaccinations administered to that individual. There have been no safety concerns to date with either dose of the trial vaccine.

 © University of Oxford, 2019

4. Objectives

Primary Objective

To assess the protective efficacy against clinical malaria of R21 adjuvanted with Matrix-M in 5-17 month old children living in a malaria-endemic area, for 6 months after the third vaccination.

Secondary Objectives

Duration of Protective efficacy against clinical malaria
To assess the protective efficacy against clinical malaria of R21 adjuvanted with Matrix-M in 5-17 months old children living in a malaria-endemic area, for 12 months after administration of the third dose of vaccine.

To assess the protective efficacy against clinical malaria of R21 adjuvanted with Matrix-M in 5-17 months old children living in a malaria-endemic area, for 6 months after their booster vaccination.

To assess the protective efficacy against clinical malaria of R21 adjuvanted with Matrix-M in 5-17 months old children living in a malaria-endemic area, for 12 months after their booster vaccination.

Efficacy against asymptomatic P. falciparum infection
To assess the protective efficacy against asymptomatic *P. falciparum* infection of R21 adjuvanted with Matrix-M in 5-17 months old children living in a malaria-endemic area, at 12 months after administration of the third dose of vaccine.

To assess the protective efficacy against asymptomatic *P. falciparum* infection of R21 adjuvanted with Matrix-M in 5-17 months old children living in a malaria-endemic area, at 12 months after administration of the booster dose of vaccine.

Safety Objective
To assess the safety and reactogenicity of R21 adjuvanted with Matrix-M in 5-17 months old children living in a malaria-endemic area, in the month following each vaccination and at 12 months after administration of the third dose of vaccine.

To assess the safety and reactogenicity of R21 adjuvanted with Matrix-M in 5-17 months old children living in a malaria-endemic area, in the month following each vaccination and at 12 months after administration of the booster dose of vaccine.

Immunogenicity Objectives
To assess the humoral immunogenicity of R21 adjuvanted with Matrix-M in 5-17 months old children living in a malaria-endemic area.

Exploratory Objectives

 © University of Oxford, 2019

<u>Efficacy against incident cases of severe malaria</u>

1. To assess the protective efficacy against severe malaria of R21 adjuvanted with Matrix-M in 5-17 months old infants living in a malaria-endemic area, at 6 months after administration of the third dose of vaccine.
2. To assess the protective efficacy against severe malaria of R21 adjuvanted with Matrix-M in 5-17 months old infants living in a malaria-endemic area, at 6 months after administration of the booster dose of vaccine.
3. To evaluate cellular immunogenicity and other exploratory immunological end points.

In all safety, immunogenicity and efficacy assessments we will compare groups 1 and 2 to the control group, group 3, both as separate groups and as a combined group 1 and 2. (assuming no difference between groups)

© University of Oxford, 2019

5. DESCRIPTION AND JUSTIFICATION OF STUDY DESIGN

Overview

A double blind randomised controlled trial is proposed to evaluate the efficacy of R21 adjuvanted with Matrix-M in healthy 5-17 month old children in a malaria endemic area.

Blinding and randomisation

Participants will be randomised 1:1:1 to receive vaccination with the IMP (R21 adjuvanted with Matrix-M; Groups 1&2) or control vaccination with Rabies Vaccine (Group 3). Participants and investigators will be blinded to group allocation for each participant. Efficacy of vaccination will be assessed by comparing the development of malaria between Groups 1 & 2 versus Group 3 participants.

Vaccinations

There are two study vaccines: the IMP, R21 adjuvanted with Matrix-M; and Rabies Vaccine. Group 1 (active vaccine group) participants will receive 3 vaccinations of R21 5µg with 25µg Matrix-M and Group 2 will receive 3 vaccinations of R21 5µg with 50µg Matrix-M , 4 weeks apart via the intramuscular route. The same thigh will be used for vaccinations.

Group 3 (control group) participants will receive three vaccinations with rabies vaccine, four weeks apart, all given intramuscularly. The same thigh will be used for these vaccinations. Rabies vaccinations should provide some protection against rabies and are anticipated to be well tolerated. (42, 43) They are expected to cause some local and systemic reactogenicity, but this will facilitate the blinding of investigators to whether the participant received Rabies vaccination or R21 adjuvanted with Matrix-M.

There will be a minimum one-week interval between administration of any study vaccine and any EPI vaccine. This is as a precaution to avoid interference between the immunogenicity of the vaccines, and also to facilitate assessment of study vaccine-related AEs, independent of EPI vaccine- related AEs.

All participants will be offered the rabies vaccination by the end of the trial so that any benefit of reduced susceptibility to rabies through vaccination is not provided selectively to the control group.

Assessment of endpoints

Safety endpoints

The specific endpoints for safety and reactogenicity will be actively and passively collected data on adverse events.
The following parameters will be assessed for all study groups

- Occurrence of solicited local reactogenicity signs and symptoms for 7 days following the vaccination

 © University of Oxford, 2019

- Occurrence of solicited systemic reactogenicity signs and symptoms for 7 days following the vaccination
- Occurrence of unsolicited adverse events for 28 days following the vaccination
- Change from baseline for safety laboratory measures
- Occurrence of serious adverse events during the whole study duration

Immunogenicity endpoints

- Comparison of immunogenicity (antibody responses to CSP) in the R21/MM vaccination group with those in the rabies vaccine group and the durability of responses
- ELISA to quantify antibodies to the vaccine components (regions of the CS antigen including the NANP repeat region and other elements of the protein as well as anti HBs).
- Flow cytometry assays with intracellular cytokine staining to enumerate and functionally characterise immune cell populations such as effector and memory T cells (e.g. CD4+ and CD8+), T follicular helper cells, regulatory T cells, B cells, plasma cells and dendritic cells
- ELISPOT for enumeration of antibody-secreting cells (e.g. B and plasma cells)

Exploratory Investigations may include:

- Assays to measure antiobody function such as inhibition of sporozoite invasion of hepatocytes.
- Assays to assess immunological aging, dysregulation and senescence, such as telomere length or expression of relevant markers and transcription factors.
- Assays to assess presence or absence of other factors affecting vaccine immunogenicity, such as antibodies against viral pathogens including cytomegalovirus.
- Other ELISA assays for immunity to malaria that may be relevant to prior malaria exposure and be used to predict vaccine immunogenicity.
- DNA extraction and sequencing to determine differences in gut microbiome between those who respond to vaccination and those who do not respond.
- Genetic tests-determination of human HLA-type and genotyping of any other genes to assess if they have an impact on response to vaccination, including genome-wide SNP and sequence analysis. N.B. Specific consent for genetic testing will be sought through an additional question on the ICF to make clear to participants and parents that consent for genetic testing doesn't not affect participation in the clinical trial.
- Genetic testing of the DNA of malaria parasites identified during the study to determine if the vaccine preferentially protects against specific genetic types of P. falciparum.

Efficacy Endpoints

Primary case definition of clinical malaria episode:
Presence of axillary temperature ≥37.5°C AND *P. falciparum* parasites density > 5000 asexual forms/µL

Secondary case definitions of clinical malaria episode:
Presence of axillary temperature ≥37.5°C and/ or history of fever within the last 24 hours AND *P. falciparum* parasites density > 0

Primary case definition of asymptomatic P. falciparum infection:

 © University of Oxford, 2019

Presence of axillary temperature < 37.5°C and absence of history of fever within the last 24 hours; AND *P. falciparum* parasites density > 0 asexual forms/µL

Primary case definition of severe malaria:
Presence of *P. falciparum* parasites density > 5000 asexuals forms/µL; AND one of more of the following criteria of disease severity:

- Prostration
- Respiratory distress
- Blantyre coma score ≤ 3
- Seizures: 2 or more
- Hypoglycemia < 2.2 mmol/L
- Acidosis BE ≤-8.0 mmol/L
- Lactate ≥ 5.0 mmol/L
- Anemia < 5.0 g/dL
- Acute kidney injury
- Pulmonary oedema
- Significant bleeding
- Shock (systolic BP <70mm Hg); AND

-Without any of the following criteria of co- morbidity

- Pneumonia (confirmed by X-ray)
- Meningitis (confirmed by CSF examination)
- Sepsis (with Positive blood culture)
- Gastroenteritis with dehydration

Secondary case definitions of severe malaria:

a) Presence of *P. falciparum* parasites density > 5000 AND one or more of the following criteria of disease severity:

  - Prostration
  - Respiratory distress
  - Blantyre coma score ≤ 3
  - Seizures 2 or more
  - Hypoglycemia < 2.2 mmol/L
  - Acidosis BE ≤-8.0 mmol/L
  - Lactate ≥ 5.0 mmol/L
  - Anemia < 5.0 g/dL
- Acute kidney injury
- Pulmonary oedema
- Significant bleeding
- Shock (systolic BP <70mm Hg)

b) Presence of:

-*P. falciparum* parasites density > 0 AND one or more of the following criteria of disease severity:

  - Prostration

 © University of Oxford, 2019

    - Respiratory distress
    - Blantyre score ≤ 3
    - Seizures 2 or more
    - Hypoglycemia < 2.2 mmol/L
    - Acidosis BE ≤-8.0 mmol/L
    - Lactate ≥ 5.0 mmol/L
- Acute kidney injury
- Pulmonary oedema
- Significant bleeding
- Shock (systolic BP <70mm Hg)
    - Anemia < 5.0 g/dL; AND

-Without any of the following criteria of co morbidity

- Pneumonia (confirmed by X-ray)
- Meningitis (confirmed by CSF examination)
- Sepsis (Positive blood culture)
- Gastroenteritis with dehydration

Either definition a) or definition b) is sufficient for a secondary definition diagnosis of severe malaria

Study site

The study will take place at the Nanoro trial site, which is located about 90 km from Ouagadougou, the capital city of Burkina Faso. Nanoro is a rural area and the Nanoro Health and Demographic Surveillance System (HDSS) covers 24 villages. In this HDSS catchment area, health care is provided by 7 peripheral health posts and one referral hospital. The population under surveillance is just over 63,000 inhabitants. The IRSS-URCN, is the research unit based in Nanoro. From recent surveys, the bed net coverage was 80%. Seasonal Malaria Chemoprophylaxis in children from 3 to 59 months was implemented since 2017. However there is no implementation of Indoor Residual Spray or IPT in infants in the area. To date there is no evidence of the decline in malaria incidence that has been recently reported from other parts of sub-Saharan Africa. In Burkina Faso, malaria is endemic. Transmission occurs throughout the year, with a peak during the rainy season (June to November). *P. falciparum* is responsible for more than 90% of all clinical malaria cases. The major vectors are *Anopheles gambiae, An. arabiensis and An. funestus*. Children under five years and pregnant women are the populations at highest risk. In Burkina Faso, during the six months when transmission reaches a peak, individuals in these age-brackets may suffer multiple malaria episodes, with an annual malaria death toll reaching over 4, 000 people in 2017. The total number of cases for 2017 was 11 915 816 with cases in children under 5 years reaching 6 082 215. There were approximately 480,000 hospital admissions with 215, 000 of these being children under 5 years. (44)

Sample size

The primary endpoint is the time to first episode of malaria meeting the primary case definition of clinical malaria episode, over a period of 6 months. The study is powered to provide an initial point estimate of the efficacy of the malaria vaccine, assuming that the vaccine efficacy over 6 months will

 © University of Oxford, 2019

be greater than 50%. We will aim to time vaccinations so that the final vaccination happens preferably near the beginning of the malaria season, which runs roughly from June to November.

Rationale for the use of passive surveillance in the trial site

For the assessment of the efficacy objectives, occurrence of malaria will be ascertained through passive surveillance (detailed in Section 8). A previous pilot study was conducted in Banfora to assess the incidence of malaria episodes using passive surveillance and active surveillance. In the active surveillance cohort, children were visited twice a week at home by the research team to detect clinical malaria episodes. In the passive surveillance cohort, the caregivers were encouraged to take their child to the local health facility where the research team was based at any time the child felt sick.

The incidence of clinical malaria was 0.09 episodes per child per month at risk (95% CI [0.08, 0.11]) in the active cohort compared to 0.09 episodes per child per month at risk (95% CI [0.07, 0.11] in the passive cohort. The passive cohort was therefore found to be the most cost-effective approach for use in future trials having clinical malaria as an efficacy endpoint.

For safety reasons, children will be visited by a field worker at 30 days intervals post vaccination up to 6 months, to follow-up adverse events related to vaccination or possible malaria cases.

Blinding and randomisation

Double-blinding will be used to reduce bias in evaluating the study endpoints. Double-blinding in this context means that the vaccine recipient, their parent(s)/guardian(s), all investigators and the study team responsible for the evaluation of efficacy, safety and immunogenicity endpoints will all be unaware of the exact treatment, (IMP or rabies vaccine) given to the participant. The only study staff aware of the vaccine assignment for IMP or rabies vaccine will be those responsible for the storage and preparation of vaccines; these staff will play no other role in the study. The vaccines will be different in terms of volume and colour. Therefore, the contents of the syringe will be masked with an opaque label to ensure that parent(s)/guardian(s), as well as nurse administering the vaccine are blinded.

Consenting participants who have satisfied all the eligibility criteria and completed the baseline assessment will be individually randomised to one of three study groups using a pre-printed envelope system. Participants will not be randomised until after consent has been taken and baseline assessments have been completed. Randomisation will use a 1:1:1 allocation when all participants have been recruited.

Allocation will be carried out using using sequentially numbered opaque sealed envelopes. An independent statistician will generate a random allocation list using block randomisation with variable block sizes. A person independent of the trial will prepare and seal the envelopes using this list, and then provide to the investigator. The independent statistician will not be part of the study team.

The study pharmacists will only be allowed to access to open an envelope after ensuring that the child before them has met all eligibility criteria and has been given a study ID number. For each

 © University of Oxford, 2019

child, eligibility will have to be counter checked and signed by a second person before allocation of study ID number. All envelopes will be retained to be checked by the clinical monitor.

The local safety monitor, who is independent from the study team, will also be provided with the allocations of Groups 1,2 &3. If deemed necessary for reasons such as safety, the Local Safety monitor will unblind the specific enrolled subject without revealing the study group to the investigators.

Study duration and timeline

Proposed timeline for the study:

Date	Activity
April 2019	Commencement of Recruitment
May 2019	First vaccination of Group 1, 2 & 3 participants
June 2019	Second vaccination of Group 1, 2 & 3 participants
July 2019	Third vaccination of Group 1, 2 and 3 participants
January 2020	Collection of endpoints for the primary analysis of efficacy
April-June 2020	Booster vaccination of Group 1, 2 and 3 participants
July-August 2020	Efficacy, safety, and immunogenicity follow-up
November-December 2020	Efficacy, safety, and immunogenicity follow-up
June 2021	Efficacy*, safety, and immunogenicity follow-up

Depending on whether significant efficacy is seen, and the potential of the vaccines for licensure, we may consider extending the follow up further for pharmacovigilance purposes. In such a case, informed consent will be obtained from study participants.

* Depending on the outcome of the efficacy analysis of the 12 months data, this may be extended to 24 months following completion of vaccinations, subject to obtaining all required approvals from the relevant ethical and regulatory authorities.

Risks and Benefits

The risks of study participation are those relating to vaccination and blood sampling.

Participating infants will receive three vaccinations with licensed rabies vaccine four weeks apart, or vaccination with R21 adjuvanted with Matrix-M, 4 weeks apart. Rabies vaccination is expected to be generally well tolerated. It may cause local reactions at the injection site such as pain and swelling or induration, and less commonly there may be fever as a systemic reaction. These reactions should generally be mild and resolve completely.

R21 adjuvanted with Matrix-M to date has been safe and well tolerated. The majority of AEs were self-limiting and mild in severity. Vaccine injection site pain was the most common local adverse event and was predominantly mild in severity. Systemic adverse events have included fever, myalgia, fatigue and malaise but these have not been common.

As with any vaccine, serious allergic reactions including anaphylaxis may occur. Such problems are very rare events with any vaccine and have never occurred with R21 adjuvanted with Matrix-M.

 © University of Oxford, 2019

Volunteers will be vaccinated in a clinical area where Advanced Life Support drugs and equipment are immediately available for the management of serious adverse reactions.

Blood collection may be associated with some discomfort and local bruising. The volume of blood collected for the research will not exceed 1ml/kg at any one time, and will not exceed 3-4 such blood samplings over eight weeks. These blood volumes are anticipated to be acceptable to parents/guardians and safe for the infants deemed eligible to participate in this study.

Participants will not benefit directly from vaccination with R21 adjuvanted with Matrix-M, but may be afforded some protection against rabies by the rabies vaccine. Parents/guardians of participating infants will be counselled that they should not expect that study vaccination will provide any protection against malaria, and that participating in the study does not reduce the need for preventive measures against malaria.

 © University of Oxford, 2019

6. INCLUSION AND EXCLUSION CRITERIA

The inclusion criteria will be used at Screening (see study procedures, Section 8) to identify participants eligible for the study, and will be checked prior to vaccination to confirm ongoing eligibility. Eligible infants will fulfil all of the inclusion criteria and none of the exclusion criteria.

Inclusion Criteria

1. Healthy child aged 5-17 months at the time of first study vaccination
2. Provide written Informed consent of parent/guardian
3. Child and parent/guardian resident in the study area villages and anticipated to be available for vaccination and follow-up for 2 years following last dose of vaccination

Exclusion Criteria

Any of the following constitutes an exclusion criterion:

- Clinically significant skin disorder (psoriasis, contact dermatitis etc.), immunodeficiency, cardiovascular disease, respiratory disease, endocrine disorder, liver disease, renal disease, gastrointestinal disease, neurological illness.
- Weight-for-age *Z* score of less than –3 or other clinical signs of malnutrition.
- History of allergic reaction, significant IgE-mediated event, or anaphylaxis to immunisation.
- History of allergic disease or reactions likely to be exacerbated by any component of the vaccines, e.g. egg products, neomycin.

- Clinically significant laboratory abnormality as judged by the study clinician.
- Blood transfusion within one month of enrolment.
- Administration of immunoglobulins and/or any blood products within the three months preceding the planned administration of the vaccine candidate.
- Previous vaccination with experimental malaria vaccines.
- Participation in another research study involving receipt of an investigational product in the 30 days preceding enrolment, or planned use during the study period.
- Current participation in another clinical trial, or within 12 weeks of this study.
- Known maternal HIV infection (No testing will be done by the study team).
- Any confirmed or suspected immunosuppressive or immunodeficient state, including HIV infection; asplenia; recurrent, severe infections and chronic (more than 14 days) immunosuppressant medication within the past 6 months (For corticosteroids, this will mean prednisone, or equivalent, ≥ 0.5 mg/kg/day. Inhaled and topical steroids are allowed).
- Any significant disease, disorder or situation which, in the opinion of the Investigator, may either put the participants at risk because of participation in the trial, or may influence the result of the trial, or the participant’s ability to participate in the trial.

Indications for delayed vaccination.

The following adverse events constitute contraindications to administration of vaccine at that point in time. If any one of these adverse events occurs at the time scheduled for vaccination, the subject

 © University of Oxford, 2019

may be vaccinated later, or withdrawn at the discretion of the investigator. The subject must be followed until resolution or stabilization of the adverse event or until causality is determined to be unrelated to trial interventions, as with any adverse event.

- Acute disease at the time of vaccination. (Acute disease is defined as the presence of moderate or severe illness with or without fever). Vaccines can be administered to persons with a minor illness such as diarrhoea, mild upper respiratory infection with or without low-grade febrile illness, i.e., temperature of <37.5°C/99.5°F).
- Temperature of ≥37.5°C (99.5°F) at the time of vaccination

NB: Anaphylactic reaction following administration of study vaccine constitutes an absolute contraindication to further administration of vaccine, and the subject must be withdrawn and followed until resolution of the event

Managing withdrawals

In accordance with the principles of the current revision of the Declaration of Helsinki (updated 2013) and any other applicable regulations, a participant has the right to withdraw from the study at any time, and for any reason, and is not obliged to give his or her reasons for doing so. The Investigator may withdraw the participant at any time in the interests of the participant's health and well-being. In addition, the participant may withdraw/be withdrawn for any of the following reasons:

Participants may be withdrawn from the study:

- By withdrawing consent
- On the decision of the investigator
- On the advice of data safety and monitoring board (DSMB)
- Any adverse event which results in the inability to comply with study procedures.
- Ineligibility either arising during the study or retrospectively (having been overlooked during screening).
- Significant protocol deviation.
- Loss to follow up (applies to a subject who consistently does not return for protocol study visits, is not reachable by telephone or any other means of communication and/ is not able to be located).

If a subject is withdrawn for any reason, the reason will be recorded. If withdrawal is the result of a serious AE, the investigator will offer to arrange for appropriate specialist management of the problem and the ethical committee will be informed in a timely manner. The extent of follow up will be determined by a medically qualified investigator but will be at least for the whole study period. Subjects withdrawn prematurely for any reason will not receive further vaccinations, although they may be requested to come back to the clinic for safety evaluation.

If a participant withdraws from the study, blood samples collected before his/her withdrawal from the trial will be used/stored unless the participant specifically requests otherwise. In all cases of subject withdrawal, apart from those of complete consent withdrawal, long-term safety data collection for vaccinated participants, including some procedures such as safety blood investigations, will continue so far as the participants are willing to consent. Where participants withdraw consent for follow up, this will be respected and follow up will be discontinued. If a participant withdraws/is

 © University of Oxford, 2019

withdrawn before completing a full vaccination course (3-doses) they will be replaced if this permits within the vaccination schedule.

 © University of Oxford, 2019

7. Investigational Medicinal Products

There are two study vaccines: the investigational medicinal product, R21 adjuvanted with Matrix-M, and the rabies vaccine.

Formulation and Dose of Investigational Medicinal Product

Description of R21
R21 has been developed at the Jenner Institute, University of Oxford. It is produced by using recombinant HBsAg particles expressing the central repeat and the C-terminus of the circumsporozoite protein (CSP). R21 was originally GMP manufactured at The Clinical Biomanufacturing Facility (CBF) in Oxford in *Pichia pastoris* and is now being manufactured at the Serum Institute of India (SII) in *Hansenula polymorphaat. R21* is a biosimilar protein particle to RTS,S which also targets the pre-erythrocytic circumsporozoite protein, the major functional protein in sporozoite development and hepatocyte invasion. It is 14 amino acids smaller than the RTS fusion protein at the C-terminus of the CSP sequence and lacks the excess of HBsAg in RTS,S (See Figure 2).

R21 will be used at a dose of 5µg.

Formulation and packaging
R21 vaccine is in formulation buffer and the drug product is filled into 2mL glass vials with a 13 mm grey bromobutyl rubber freeze-dry stopper (CE Marked, supplied by Adelphi Tubes) and a 13 mm complete tear, clear lacquered aluminium seal. The nitrogen filled vials are supplied sterile. The containers and closures are tested for compliance with defined specifications.

Matrix-M is formulated at a concentration of 1 mg/mL in PBS. The drug product is filled into sterile brown glass vials.

Storage and handling of Investigational Medicinal Products

Long term, R21 vaccine is stored frozen at a nominal temperature of -80°C and Matrix-M is stored between 2-8°C or with Matrix-M requiring protection from light.

All movements of the vaccines and adjuvants will be documented. Accountability, storage, shipment and handling of R21 and Matrix-M will be in accordance with relevant local SOPs and forms.

Dispensing and administration of study vaccines

On vaccination day, R21 will be allowed to thaw to room temperature. It will then be mixed with Matrix-M and administered within 1 hour of removal from the freezer and Matrix-M from the fridge. All participants will be observed in the unit for 60 minutes (+/- 15 minutes) after vaccination.

During administration of the investigational products, Advanced Life Support drugs and resuscitation equipment will be immediately available for the management of anaphylaxis. Vaccination will be performed, and the vaccine handled according to the relevant SOPs.

 © University of Oxford, 2019

Dose Administration

The vaccine will be administered into the left thigh in the subjects. Vaccine preparation will be undertaken under aseptic conditions by the pharmacist together with the vaccinating nurse.

Accountability

There will be accountability logs kept in the pharmacy or storage area as well as during the vaccination sessions. These will be reconciled at the end of each day.

Concomitant Medication

Concomitant therapies will be recorded at all visits.

 © University of Oxford, 2019

8. Study schedule and procedures

Identification of Study Participants

Community sensitization will be undertaken to engage the community with the study and recruit volunteers for participation in the study. Volunteers will be assessed at screening visits to determine if they are eligible to participate in the study.

Community sensitisation

The IRSS-URCN social science team will hold local community meetings and explain the study to the parents/guardians of potentially eligible children. During these meetings the investigators will explain the following: the need for a vaccine (including a simple picture of the burden of malaria on the community); the current status of vaccine development (including the fact that this is likely to be a prolonged process); the study screening and informed consent procedure; risks of vaccination and the unproven benefits of vaccination. It will be stressed that this is an experimental vaccine and cannot be guaranteed to provide protection, and that it will therefore still be necessary to seek treatment for possible malaria even after vaccination and continue to use other protective measures such as bed nets. It will be made clear that neither parents/guardians, nor investigators will know which vaccination regimen the child has received until the end of the study. It will be explained that a photograph of the child and parent/guardian will be taken if they are eligible to be enrolled in the trial, to aid identification.

After this meeting based on the list of children of suitable age for participation in the trial drawn from the HDSS database, parents/guardians will be asked to participate in the study and will be invited for a screening visit.

Screening Visit

We will provide detailed information about the study for distribution to the parents/guardians. The investigators will endeavour to ensure that all carers fully understand the risks. Any carer who appears to have less than complete understanding will be considered unable to give consent. As with any experimental vaccine the parents/guardians must understand that the vaccines have not yet been shown to prevent infection and this will be stressed during the recruitment stage. They must also understand the very small chance of anaphylactic reactions and thereby the importance of complying with the one-hour observation period after each vaccination. The information sheet covers these points in detail, and each parent/guardians will have the contents of the sheet explained in individual meetings.

If it is determined by the investigator conducting the screening visit that free and informed consent is given by the parent/guardian for their child to participate in the trial, the parent/guardian will be asked to complete the consent form. The parent/guardian will thumbprint the consent form if illiterate.

A literate, impartial witness will be present for screening procedures and countersign the consent form if the parents/carers are illiterate.

 © University of Oxford, 2019

Children of parents/guardians who have consented will undergo the full screening procedures. This consists of medical history, physical examination, and blood sampling for screening tests as detailed below (Laboratory Evaluations). Medical history will also include information about infant's nutrition/diet/method of feeding/age of weaning/mode of delivery/number of children in household.

The investigator will determine whether the child is eligible to participate in the study, using the findings at screening, including the results of the screening blood tests. Children eligible to participate in the study will fulfil all of the inclusion criteria, and meet none of the exclusion criteria.

Screening visit (Boost)

A similar visit will occur prior to the boost vaccination (fourth vaccination) and children of parents/guardians who have consented will undergo the full screening procedures. This consists of medical history, physical examination, and blood sampling for screening tests as detailed below (Laboratory Evaluations). This will take place up to 30 days prior to booster vaccination. Medical history will also include information about infant's nutrition/diet/method of feeding/age of weaning/mode of delivery/number of children in household.

The interim safety data to Day 14 of trial participation after the first 30 vaccinations of participants across Groups 1,2 & 3 will be presented to the DSMB for review of safety. There will be no planned pause of the trial to allow review but should there be any concerns, the DSMB, as always, are empowered to pause or stop the trial should they have relevant concerns. The DSMB will be empowered to decode the vaccination group of any subject or subjects as required if there are relevant safety concerns.

Study Visits

Table 1 and 2 show the window periods for the visits and outlines the study procedures at each visit for all study groups.

Day 0 (Vaccination)

This visit will occur not more than 30 days following the screening visit. If more than 30 days have lapsed since screening, then a repeat Screening Visit will be conducted. Medical history, physical observations +/- physical examination will be performed.

Ongoing eligibility for participation will be confirmed according to the inclusion and exclusion criteria vaccination.

Children are considered enrolled into the study when they receive the first study vaccination. The vaccine will be administered as detailed in Section 7 and according to local SOPs.

Following vaccination, the vaccination site will be covered with a dressing which will be removed after 60 minutes (+/- 15 minutes) . The volunteer will be monitored for one hour in total (or longer if necessary) after vaccination.

 © University of Oxford, 2019

A rectal swab/faecal sample will be taken as part of the exploratory immunology analysis.

The CRF will be updated.

Days 1-6

Each subject will be visited at home daily by a field worker for assessment and recording of any solicited and unsolicited AEs. If necessary the volunteer will continue to be seen regularly until the AEs have resolved or stabilised.

Day 7

Medical history, physical observations +/- physical examination will be performed.

The CRF will be updated, including the records of AEs and concomitant medications.

If axillary temperature ≥ 37.5°C and/or history of fever within the last 24 hours, with no other source of infection, a blood smear will be obtained for malaria diagnosis.

Day 28 (Vaccination)

Medical history, physical observations +/- physical examination will be performed. Ongoing eligibility will be confirmed by the Investigator according to the inclusion and exclusion criteria, prior to blood sampling and vaccination. Blood sampling will be performed as detailed below (Laboratory Evaluations).

Study vaccine will be administered as detailed in Section 7 and according to local SOPs. Following vaccination, the vaccination site will be covered with a dressing which will be removed after 60 minutes (+/- 15 minutes). The volunteer will be monitored for one hour in total (or longer if necessary) after vaccination.

The CRF will be updated, including the records of AEs and concomitant medications.

If axillary temperature ≥ 37.5°C and/or history of fever within the last 24 hours, with no other source of infection, a blood smear will be obtained for malaria diagnosis.

The fieldworker will document the bed net use and residual spraying.

Days 29-34

Each subject will be visited at home daily by a field worker for assessment and recording of any solicited and unsolicited AEs. If necessary the volunteer will continue to be seen regularly until the AEs have resolved or stabilised.

Day 35

Medical history, physical observations +/- physical examination will be performed.

The CRF will be updated, including the records of AEs and concomitant medications.

 © University of Oxford, 2019

If axillary temperature ≥ 37.5°C and/or history of fever within the last 24 hours, with no other source of infection, a blood smear will be obtained for malaria diagnosis.

Day 56 (Vaccination)

Medical history, physical observations +/- physical examination will be performed. Ongoing eligibility will be confirmed by the Investigator according to the inclusion and exclusion criteria, prior to blood sampling and vaccination. Blood sampling will be performed as detailed below (Laboratory Evaluations).

Study vaccine will be administered as detailed in Section 7 and according to local SOPs. Following vaccination, the vaccination site will be covered with a dressing which will be removed after 60 minutes (+/- 15 minutes). The volunteer will be monitored for one hour in total (or longer if necessary) after vaccination.

The CRF will be updated, including the records of AEs and concomitant medications.

If axillary temperature ≥ 37.5°C and/or History of fever within the last 24 hours, with no other source of infection, a blood smear will be obtained for malaria diagnosis.

Days 57-62

Each subject will be visited at home daily for three days by a field worker for assessment and recording of any solicited and unsolicited AEs. If necessary the volunteer will continue to be seen regularly until the AEs have resolved or stabilised.

At the Day 57 visit, the fieldworker will document the bed net use and residual spraying.

Day 63

Medical history, physical observations +/- physical examination will be performed.

The CRF will be updated, including the records of AEs and concomitant medications.

If axillary temperature ≥ 37.5°C and/or history of fever within the last 24 hours, with no other source of infection, a blood smear will be obtained for malaria diagnosis.

Day 84

Medical history, physical observations +/- physical examination will be performed.

Blood sampling will be performed as detailed below (Laboratory Evaluations). The CRF will be updated, including the records of AEs and concomitant medications.

If axillary temperature ≥ 37.5°C and/or history of fever within the last 24 hours, with no other source of infection, a blood smear will be obtained for malaria diagnosis.

The fieldworker will document the bed net use and residual spraying.

 © University of Oxford, 2019

Days 114, 144, 174, 204

Each subject will be visited at home every 30 days by a field worker for assessment and recording of the subject health status. All the children found febrile/history of fever within the last 24 hours will be referred to the research centre where a blood smear will be obtained for malaria diagnosis, if no other source of infection or reason for the fever is found. Any serious adverse event not detected will be documented. If necessary, the volunteer will continue to be seen regularly until the AEs have resolved or stabilised.

The fieldworker will document the bed net use and residual spraying at these visits.

Day 236

Medical history, physical observations +/- physical examination will be performed.

Blood sampling will be performed as detailed below (Laboratory Evaluations). The CRF will be updated, including the records of SAEs and concomitant medications.

A blood smear will be obtained for malaria diagnosis.

If the participant has received SMC, this will be documented in the CRF.

Day 421

Medical history, physical observations +/- physical examination will be performed.

Blood sampling will be performed as detailed below (Laboratory Evaluations). The CRF will be updated, including the records of SAEs and concomitant medications.

A blood smear will be obtained for malaria diagnosis.

If the participant has received SMC, this will be documented in the CRF.

Boost (Vaccination)

Medical history, physical observations +/- physical examination will be performed. Ongoing eligibility will be confirmed by the Investigator according to the inclusion and exclusion criteria, prior to vaccination.

Study vaccine will be administered as detailed in Section 7 and according to local SOPs. Following vaccination, the vaccination site will be covered with a dressing which will be removed after 60 minutes (+/- 15 minutes). The volunteer will be monitored for one hour in total (or longer if necessary) after vaccination.

The CRF will be updated, including the records of AEs and concomitant medications.
A rectal swab/faecal sample will be taken as part of the exploratory immunology analysis.

If axillary temperature ≥ 37.5°C and/or history of fever within the last 24 hours, with no other source of infection, a blood smear will be obtained for malaria diagnosis.

 © University of Oxford, 2019

Bed net use and residual spraying will be documented.

Note:
Booster vaccinations are due to take place prior to the malaria season one year following the third vaccination. These visits are expected in the months of April-June. If visit 31 at D421 from Table 1 coincides in the window with S (B) visit at B-30-B-1 or B+1 visit at B, these visits will be merged and procedures such as blood sampling will take place only once

Boost + day 1-6

Each subject will be visited at home daily by a field worker for assessment and recording of any solicited and unsolicited AEs. If necessary the volunteer will continue to be seen regularly until the AEs have resolved or stabilised.

Boost + day 7

Medical history, physical observations +/- physical examination will be performed.

The CRF will be updated, including the records of AEs and concomitant medications.

If axillary temperature ≥ 37.5°C and/or history of fever within the last 24 hours, with no other source of infection, a blood smear will be obtained for malaria diagnosis.

Boost + day 28

Medical history, physical observations +/- physical examination will be performed.

The CRF will be updated, including the records of AEs and concomitant medications.

If axillary temperature ≥ 37.5°C and/or history of fever within the last 24 hours, with no other source of infection, a blood smear will be obtained for malaria diagnosis.

Bed net use and residual spraying will be documented.

Boost + day 168

Medical history, physical observations +/- physical examination will be performed.

Blood sampling will be performed as detailed below (Laboratory Evaluations). The CRF will be updated, including the records of SAEs and concomitant medications.

A blood smear will be obtained for malaria diagnosis.

If the participant has received SMC, this will be documented in the CRF.

Boost + day 336

Medical history, physical observations +/- physical examination will be performed.

 © University of Oxford, 2019

Blood sampling will be performed as detailed below (Laboratory Evaluations). The CRF will be updated, including the records of SAEs and concomitant medications.

A blood smear will be obtained for malaria diagnosis.

If the participant has received SMC, this will be documented in the CRF.

Laboratory Evaluations

Table 6-7, below, shows the Study Visits at which volunteers will have blood films for malaria diagnosis, and blood sampling for haematology, biochemistry, and exploratory immunology.

 © University of Oxford, 2019

**Study visit number**	**S**	**1**	**2-7**	**8**	**9**	**10-15**	**16**	**17**	**18-23**	**24**	**25**	**26**	**27**	**28**	**29**	**30**	**31**
**Clinic visit**	X	X		X	X		X	X		X	X					X	X
**Home visit**			X			X			X			X	X	X	X		
**Day of visit**	D-30-D-1	D0	D1-6	D7	D28	D29-34	D35	D56	D57-62	D63	D84	D114	D144	D174	D204	D236	D421
**Window period**				+/- 1	+/- 3		+/- 1	+/- 1		+/- 1	+/- 3	+/- 7	+/- 7	+/- 7	+/- 7	+/- 28	+/- 56
**Vaccination**		X			X			X									
**Blood film for Plasmodium species**	X															X	X
**Blood film for Plasmodium species if axillary temp** ≥37.5 and/or history of fever within last 24 hours				X	X		X	X		X	X	X	X	X	X		
**Haematology & Biochemistry**	X				X						X					X	X
**Immunology**	X				X						X					X	X

Table 6: Timeline of Study Visits showing blood sampling and laboratory investigations for participants in Groups 1,2 & 3

**Study visit number**	**S (B)**	**B1**	**B2-7**	**B8**	**B9**	**B10**	**B11**
**Clinic visit**	X	X		X	X	X	X
**Home visit**			X				

 © University of Oxford, 2019

**Day of visit**	B-30-B-1	B0	B1-6	B7	B28	B168	B336
**Window period**				+/- 1	+/- 3	+/- 28	+/- 28
**Vaccination**		X					
**Blood film for Plasmodium species**	X					X	X
**Blood film for Plasmodium species if axillary temp** ≥37.5 and/or history of fever within last 24 hours		X		X	X		
**Haematology & Biochemistry**	X				X	X	X
**Immunology**	X				X	X	X

Table 7: Timeline of Study Visits showing blood sampling and laboratory investigations for participants in Groups 1, 2 & 3 receiving a booster (fourth) vaccination prior to the malaria season, the year after the first 3 vaccinations

Note:

Booster vaccinations are due to take place prior to the malaria season approximately one year following third vaccination. These are expected in the months of April-June. If visit 31 at D421 from Table 1 coincides in the window with S (B) visit at B-30-B-1 or B+1 visit at B, these visits will be merged and procedures such as blood sampling will take place only once

© University of Oxford, 2019

Each study visit will occur the indicated number of days from Day 0, within the window period for that visit.
S: Screening Visit, X: procedure takes place, D: Day.

Descriptions of Blood sampling and Laboratory Evaluations

Blood films for *Plasmodium species (falciparum/ovale/malariae)*: The blood film will be prepared with venous blood where possible, to minimise volunteer discomfort. Thick blood smears will be stained with Giemsa and read by experienced microscopists based on local SOPs.

Blood will be sampled at the visits indicated in Table 5, for haematology, biochemistry, and exploratory immunology. The volume of blood per blood sampling will be a minimum of 5ml. If deemed safe by the investigators, if the participant is more than 5kg, and taking into account any other blood tests (any abnormalities) done for the routine care of the infant, the investigator's may collect 1ml/kg, up to a maximum of 8ml.

Haematology: Full Blood Count. This will be done at the study visits as indicated in Tables 5 & 6.

Biochemistry: including Creatinine, ALT, AST, Glucose and Bilirubin. This will be done at the study visits as indicated in Tables 5 & 6.

Immunology: This will be done at the study visits as indicated in Tables 5 & 6. The following investigations will be done on blood collected for immunogenicity endpoints and exploratory immunology, at the discretion of the investigators:

- Comparison of immunogenicity (antibody responses) of the R21/MM vaccination doses and the longevity of responses
- ELISA to quantify antibodies to the vaccine components CS, NANP and HBsAb.
- Functional assays to measure antibody function, such as inhibition of sporozoites invasion
- Flow cytometry assays with intracellular cytokine staining to enumerate and functionally characterise immune cell populations such as effector and memory T cells (e.g. CD4+ and CD8+), T follicular helper cells, regulatory T cells, B cells, plasma cells and dendritic cells
- ELISPOT for enumeration of antibody-secreting cells (e.g. B and plasma cells)

- Assays to assess immunological aging, dysregulation and senescence, such as telomere length or expression of relevant markers and transcription factors.
- Assays to assess presence or absence of other factors affecting vaccine immunogenicity, such as antibodies against viral pathogens including cytomegalovirus.
- Other ELISA assays for immunity to malaria that may be relevant to prior malaria exposure and be used to predict vaccine immunogenicity.
- DNA extraction and sequencing to determine differences in gut microbiome between those who respond to vaccination and those who don't.
- Genetic tests-determination of HLA-type and associated genes that can have an impact on vaccination. N.B. Specific consent genetic testing will be sought through an additional question on the ICF to make clear to participants and parents that consent for genetic testing doesn't not affect participation in the clinical trial.

Plasma, serum and cells for exploratory immunology will be stored at -20°C and -192°C respectively.

 © University of Oxford, 2019

Rectal swab/faecal sample: This will be done on the day of the first vaccination and the day of the booster vaccination.

Provision of care to the study participants

Study contact personnel will be available 24 hours a day at trial site clinic and at the different health facilities of the study population catchment areas, seven days a week, to attend if children require a consult. Children requiring inpatient care will be admitted to the hospital where study personnel will be posted. Laboratory and radiological investigation will be carried out when appropriate. If necessary, children requiring more specialized care (treatment or diagnostic procedures) will be transported to a referral hospital.

Treatment for medical conditions will be given according to the standard treatment regimens locally. Any expenses including transport incurred by the parent(s)/guardian(s) of study participants for clinical care related to acute conditions will be borne by the trial according to the appropriate local arrangements. Long-term care for chronic conditions unrelated to study procedures will be delivered following local guidelines with no financial support from the trial.

Malaria case management

Uncomplicated Malaria Cases

Trial subjects with uncomplicated malaria will be treated according to SOPs and national guidelines.

Severe Malaria Cases

Trial subjects with severe malaria will be treated according to SOPs and national guidelines.

Ascertainment of malaria endpoints

Collection of malaria endpoints for analysis of efficacy will begin at Day 236, which is 6 months following of completion of the vaccination regimen and Day 421, which is 12 months following the last vaccination.

Clinically qualified investigators will adjudicate the presence of the endpoints of clinical *P. falciparum* malaria, severe *P. falciparum* malaria, and asymptomatic *P. falciparum* carriage, before they are unblinded to group allocation.

Case Detection for Clinical *P. falciparum* malaria

For the primary efficacy endpoint, passive case detection will be used and will consist of continuous availability of medical care at the trial site and at the community clinics to which trials participant villages belongs to.

All participants presenting to health facilities in the study area will be evaluated as potential cases of clinical malaria disease. A blood sample for the evaluation of malaria parasites will be taken for all children who are reported to have had a fever within 24 hours of presentation or have a measured axillary temperature of ≥37.5°C, where no other source of fever has been found. A *P. falciparum* rapid diagnosis test will be performed to guide immediate patient management. However, efficacy results will be based on blood slide reading. The research team will be available 24 hours/ day, 7

 © University of Oxford, 2019

days a week. The participants' parents/guardians will be informed to bring the child to the health facility should the child be "unwell".

Case Detection for Severe *P. falciparum* malaria

A passive surveillance system will be implemented. All participants presenting for admission through the outpatient and emergency departments of hospitals in the study areas will be evaluated as potential cases of severe malaria disease. During the hospitalization, the participant's course will be monitored to capture the symptoms, signs and biochemical parameters indicative of severe malaria disease.

Safety follow-up

Trained field workers, under the supervision of the investigators, will visit daily each enrolled child for days 1 to 6 post vaccination. If necessary, the child will continue to be seen by the field worker on subsequent days for follow-up of adverse events. The field workers will visit the child at 30 day intervals as indicated on the Timeline of Study Visits (Table 1). In the event that the field worker finds any Grade 3 solicited general or unsolicited symptoms, the volunteer will be brought to the vaccination centre for examination by a study clinician. During the field worker visits, the children's parent(s)/guardian(s) will be asked retrospectively if any medical event that might be a SAE occurred since the last visit and this information will be recorded. Unreported SAEs detected in this way will be investigated and reported by the PI or delegate on the corresponding SAE.

If a study participant is reported to be unwell at the time of a visit, the field worker will advise the parents to report to the trial site clinic or the nearest health facility, where a study nurse will be posted and will notify this referral to the clinical team for follow up. In the event that a study participant is seriously ill, the field worker will inform the PI or designate, and transport will be arranged, to the referral hospital (where a study physician is posted), if judged appropriate by the responsible clinician.

In case a study participant is unwell and referred to the trial site clinic or health facility, a duplicate blood film will be obtained should the volunteer present symptoms or signs compatible with malaria (axillary temperature ≥ 37.5°C, history of fever within the last 24 hours, loss of appetite, malaise, vomiting and diarrhoea).

A study clinician will review the infant at the Clinic Visits, on Days 7, 28, 35, 56, 63, 84, 236, and 421, for full safety and reactogenicity assessment and possible diagnosis of malaria. They will also review the infant on Days 7, 28, 168 and 336 post the boost (fourth) vaccination.

Immunogenicity measurements

Antibody responses measured by anti-NANP IgG ELISA were performed on samples from day of screening, 7, 84, 236 and 421 following the first 3 vaccinations and Day 28, 168 and 336 following the boost vaccination. IgG antibody avidity will be assessed by sodium thiocyanate (NaSCN)-displacement ELISA.. *Ex-vivo* IFN- γ ELISpot responses to CSP will be assessed on samples from day 0, 84, 236 and 421.

Data collection

© University of Oxford, 2019

Adverse events will be documented in individual case report forms (CRFs) for each volunteer. They will be recorded under two headings: local and systemic. There will be documentation of concomitant medication, vaccinations, non-serious adverse events, serious adverse events, and study conclusion. Case report forms will be kept securely.

The following data will be collected for concomitant medications: medication name (generic name), dose, frequency and route; start and stop dates; and indication.

Concomitant medication will be recorded according to the time period below:

- Antimalarial drugs, immune- modifying drugs and blood transfusions will be captured for the duration of the trial.
- Antipyretics, analgesics, systemic antibiotics will be collected from dose 1 of vaccination until 1 month post dose 3 and from dose 1 of boost vaccination until 1 month post boost vaccination.
- All vaccines administered, not specified in the study protocol, will be recorded for the duration of the trial.

Study termination

The study will be discontinued in the event of any of the following:

- New scientific information is published to indicate that volunteers in the study are being exposed to undue risks as a result of administration of the IMPs by any route of administration, or as a result of the follow-up schedule.
- Serious concerns about the safety of the IMPs arise as a result of one or more vaccine related SAE occurring in the subjects enrolled in this or any other ongoing study of the IMPs.
- For any other reason at the discretion of the Principal Investigator.

Definition of the Start and End of the Trial

The start of the trial is defined as the date of the first vaccination of the first volunteer. The end of the trial is the date of the last visit of the last volunteer.

 © University of Oxford, 2019

9. Assessment of scientific objectives

A full detailed statistical analysis plan will be developed prior to any unblinding of the data. The first data analysis will be performed after the last enrolled participant has reached 6 months after last vaccination. Analyses for the subsequent follow-ups will be carried out when all participants have reached at least 12 months after the last vaccination. Results of these analyses will be disseminated accordingly.

Investigators performing the statistical analyses will be unblinded to Group 1 vs 2 vs 3 allocation at the end of the study once all data following enrolment of all volunteers is collected and data locked. Investigators not performing the analyses, for example those undertaking field work and interpreting adverse events and malaria endpoints, will also remain blinded until the end of the study.

The population for the statistical analyses are those participants who are eligible to participate and according to outcome as follows:

Efficacy

The primary analysis will be based on a modified intention-to-treat population. That is, all participants will be analysed in the groups to which they were randomised, regardless of which vaccine they received. They will remain in the analysis regardless of how many trial visits they ave attended, but only if they have received all 3 vaccinations.

An unadjusted analysis of the primary outcome will also be carried out on the per-protocol population. This will include all participants who are eligible to participate, and received all allocated vaccinations within the specified time window periods of 4 week intervals plus or minus 3 days, without any contraindications to vaccine administration.

Immunology

Participants will be included in the analysis of immunology outcomes if they have received a minimum of 3 vaccines to which they have been allocated. They do not need to have attended all follow up visits. The immunology outcomes will also be analysed separately for those receiving 2 and 3 vaccines.

Safety

The safety analysis will be based on the per protocol population (receiving all 3 doses of allocated vaccine). A secondary safety analysis will include participants who have received at least one of the 3 vaccinations, regardless of how many trial visits they have attended.

Primary analysis

The primary groups for comparison will be group 1 vs 3 and group 2 vs 3. If no statistically significant difference is found between groups 1 and 2 then a further analysis will be carried out comparing groups 1 and 2 vs. group 3.
Analysis of the 6 month outcomes will be carried out once the final participant has completed their 6 month post 14 days after dose three assessment. The 12 month outcomes will be analysed once the final participant has completed their 12 month post 14 days post dose three assessment.

 © University of Oxford, 2019

..

Kaplan Meier curves will be presented. A Cox regression model will be used to test whether time to malaria differs between the randomised groups. The median (interquartile range) for each randomised group will be presented. The model will include randomised group. Hazard ratios and 95% confidence intervals will be reported to present the difference in time to event between groups 1 and 3 and groups 2 and 3. Vaccine efficacy will be calculated as 1-HR.

A secondary analysis of the primary outcome will adjust for age, gender and days bed net use between randomisation and last vaccination.

Secondary analyses: Efficacy

The following analyses of efficacy will be performed:

-Protective efficacy against clinical malaria

- Time to first episode of malaria meeting the primary case definition of clinical malaria episode over a period of 6 and 12 months of follow-up from 14 days after the last vaccination.

- Proportion of participants with an episode of malaria meeting the primary case definition of clinical malaria episode, within the periods, 6, and 12 months of follow-up from 14 days after the last vaccination

-Efficacy against asymptomatic P. falciparum infection

- Proportion of participants meeting the primary case definition of asymptomatic *P. falciparum* infection, at study days 236 and 421.

-Efficacy against secondary case definitions of clinical malaria episode

- Time to first episode of malaria meeting the secondary case definitions of clinical malaria episode over a period of 6, and 12 months of follow-up from 14 days after the last vaccination.

- Proportion of participants with an episode of malaria meeting the secondary case definitions of clinical malaria, within a period of 6 and 12 months of follow up from 14 days after the last vaccination

Time to first or only episode of clinical malaria meeting secondary endpoint definitions will be analysed as above.

Safety analyses

All solicited and unsolicited local and systemic adverse events (including results of clinical laboratory investigations where deemed adverse events) will be listed. They will be presented according to whether they are possibly, probably or definitely related to vaccination and by vaccination group.

The proportion of patients in each group reporting any local reaction will be compared using the chi-squared test and the difference in proportions with 95% confidence intervals will be presented (comparing groups 1 and 3 and groups 2 and 3). This will be repeated for systemic reactions.

© University of Oxford, 2019

All SAEs will be described in detail for each participant. The proportion of patients in each group reporting at least one SAE will be compared using the chi-squared test and the difference in proportions with 95% confidence intervals will be presented (comparing groups 1 and 3 and groups 2 and 3).

Where a patient reports more than one of the same type of event, separate tables will be presented showing a) counts of events and b) counts of participants experiencing at least one type of this event.

Immunogenicity Analyses

Immunogenicity data will be analysed according to a detailed analytical plan.

Exploratory Analysis

The following analyses will be performed:

- Time to first episode of severe malaria meeting the primary and secondary case definitions of severe malaria over a period of 6 months of follow-up after the last vaccination.

- Proportion of participants with an episode of malaria meeting the primary and secondary case definitions of severe malaria, within a period of 6 months of follow up after the last vaccination

Analyses for efficacy against severe malaria will be similar to those described previously. These are exploratory analyses that are likely to have low statistical power.

The primary comparisons will be of group 1 vs. 3 and group 2 vs. 3. If no difference is found between groups 1 and 2 then a further, secondary analysis will be carried out comparing groups 1 and 2 combined vs. group 3.

<u>Subgroup analyses</u>

2 subgroup analyses will be conducted on the primary efficacy and safety outcomes. One will be by gender and the other by age group (5-9 months, 10-12 months and >12 months).

 © University of Oxford, 2019

10. Safety Reporting

Definitions

Definitions for the terms adverse event (or experience), adverse reaction, and unexpected adverse reaction have previously been agreed to by consensus of the more than 30 Collaborating Centres of the WHO International Drug Monitoring Centre (Uppsala, Sweden). Although those definitions can pertain to situations involving clinical investigations, some minor modifications are necessary, especially to accommodate the pre-approval, development environment.

Adverse Event

An AE is any untoward medical occurrence in a volunteer, including a dosing error, which may occur during or after administration of an IMP and does not necessarily have a causal relationship with the intervention. An AE can therefore be any unfavourable and unintended sign (including an abnormal laboratory finding), symptom or disease temporally associated with the study intervention, whether or not considered related to the study intervention.

Each adverse event will be graded by the participant according to the table for grading severity of adverse events (see Tables 6-9). Severity gradings may be reviewed and discussed with the participants at the clinic visits.

Adverse Reaction (AR)

An ADR is any untoward or unintended response to an investigational medicinal product (IMP). This means that a causal relationship between the IMP and an AE is at least a reasonable possibility, i.e., the relationship cannot be ruled out. All cases judged by either the reporting medical investigator or the sponsors as having a reasonable suspected causal relationship to an IMP (i.e. possibly, probably or definitely related to an IMP) will qualify as adverse reactions.

Serious Adverse Event (SAE)

A serious adverse event is an AE that results in any of the following outcomes, whether or not considered related to study intervention:

- Death (i.e., results in death from any cause at any time)
- Life-threatening event (i.e., the volunteer was, in the view of the investigator, at immediate risk of death from the event that occurred). This does not include an AE that, if it occurred in a more serious form, might have caused death.
- Persistent or significant disability or incapacity (i.e. substantial disruption of one’s ability to carry out normal life functions).

 © University of Oxford, 2019

- Hospitalisation or prolongation of hospitalisation, regardless of length of stay, even if it is a precautionary measure for continued observation. Hospitalisation (including inpatient or outpatient hospitalization for an elective procedure) for a pre-existing condition that has not worsened unexpectedly does not constitute a serious AE.
- An important medical event (that may not cause death, be life threatening, or require hospitalization) that may, based upon appropriate medical judgment, jeopardize the volunteer and/or require medical or surgical intervention to prevent one of the outcomes listed above.
- Congenital anomaly or birth defect

Medical and scientific judgment should be exercised in deciding whether expedited reporting is appropriate in other situations, such as important medical events that may not be immediately life-threatening or result in death or hospitalisation, but may jeopardise the patient or may require intervention to prevent one of the other outcomes listed in the definition above. These should also usually be considered serious.

Serious Adverse Reaction (SAR)

An adverse event (expected or unexpected) that is both serious and, in the opinion of the reporting investigator or sponsors, believed to be possibly, probably or definitely due to an IMP or any other study treatments, based on the information provided.

Suspected Unexpected Serious Adverse Reactions (SUSARs)

A SUSAR is an SAE that is unexpected and thought to be possibly, probably or definitely related to the investigational medicinal product. Administration of further vaccines within the trial will be suspended until a safety review is convened.

Severity assessment

The severity of clinical adverse events will be assessed according to the scales in Tables 9-11.
All local reactions will be considered causally related to the vaccination in the absence of another more likely explanation (such as recent trauma).

At each visit, parents/guardians will be requested to report local and general side effects their child might have experienced since they last were seen. The investigator will assess the severity of the solicited signs and symptoms using the key provided in Table 8. Further details for any AE (such as start/stop date and any treatment), will be gathered, regardless of the relationship to the vaccine. Episodes of malaria detected as endpoints in the efficacy evaluation will not be reported as AEs.

 © University of Oxford, 2019

We will also document any unsolicited adverse event reported by the parent/guardian. Serious adverse events (SAE) as defined above will be collected throughout the study period, documented and reported using a serious adverse event reporting form.

Grade	Description
**0**	None
**1**	Mild-Transient or mild discomfort (< 48 hours); no medical intervention/therapy required
**2**	Moderate- Mild to moderate limitation in activity - some assistance may be needed; no or minimal medical intervention/therapy required
**3**	Severe- Marked limitation in activity, some assistance usually required; medical intervention/therapy required, hospitalization possible.

Table 8: Intensity of the general adverse events solicited from home visits and clinic will be assessed as described

Grade	Diameter (mm)
**0**	0
**1**	< 5
**2**	5-20
**3**	>20

Table 9: Grading of local injection site swelling

Adverse Event	Grade	Temeprature (non-axillary)
**Pain at injection site**	0	Absent
	1	Minor reaction to touch
	2	Cries/protests on touch
	3	Cries when limb is moved/spontaneously painful
**Swelling at injection site**		Record greatest surface diameter in mm
**Redness/discoloration at injection site**		Record greatest surface diameter in mm
**Fever**		Record temperature in °C
**Irritability/Fussiness**	0	Behaviour as usual
	1	Crying more than usual, no effect on normal activity
	2	Crying more than usual, interferes with normal activity
	3	Crying that cannot be comforted, prevents normal activity
**Drowsiness**	0	Behaviour as usual
	1	Drowsiness easily tolerated
	2	Drowsiness that interferes with normal activity
	3	Drowsiness that prevents normal activity

 © University of Oxford, 2019

**Loss of appetite**	0	Appetite as usual
	1	Eating less than usual/ no effect on normal activity
	2	Eating less than usual/ interferes with normal activity
	3	Not eating at all

Table 10: Severity grading criteria for local and systemic AEs

Grade	Fever
**0**	<37.5 °C
**1**	37.5-38.0 °C
**2**	>38-39.0 °C
**3**	>39.0 °C

Table 11: Grading of fever

Severity grading of paediatric cardiovascular signs and laboratory tests will be assessed using the most up to date Division of AIDS (DAIDS) Table for Grading the Severity of Adult and Paediatric Adverse Events, currently Version 2.1 March 2017. We will use the latest version as soon as we become aware of a change in version.

Follow-up of Adverse Events

Adverse events likely to be related to the vaccine, serious or not, which persist at the end of the trial will be followed up by the investigator until their resolution or stabilisation, or until causality is determined to be unrelated to trial interventions. All AEs will be managed as per national clinical guidelines.

Moreover, any serious adverse event likely to be related to the vaccine and occurring after trial termination should be reported by the investigator according to the procedure described below.

Outcome of any non-serious adverse event occurring within 28 days post-vaccination (*i.e.* unsolicited adverse event) or any SAE reported during the entire study will be assessed as:

- Recovered/resolved
- Not recovered/not resolved
- Recovering/resolving
- Recovered with sequelae/resolved with sequelae
- Fatal (SAEs only)

Subjects who have moderate or severe on-going adverse events that are not vaccine linked will be referred to an appropriate hospital/health facility on completion of the study and will be advised to

 © University of Oxford, 2019

consult a primary care physician if the event is not considered to be related to the study vaccine. A follow-up visit will be arranged to manage the problem and to determine the severity and duration of the event, if it is related to the study vaccine.

Reporting of AEs and SAEs

Every SAE occurring throughout the trial must be reported by telephone, e-mail or fax to the sponsor and DSMB within twenty-four hours, even if the investigator considers the SAE not related to vaccination. The investigator will then complete a SAE report as soon as possible and within 5 working days or 7 calendar days.

Any relevant information concerning the adverse event that becomes available after the SAE report form has been sent (outcome, precise description of medical history, results of the investigation, copy of hospitalisation report, etc.) will be forwarded to the sponsor in a timely manner, the anonymity of the subjects shall be respected when forwarding this information.

The DSMB may ask for the study to be stopped, or for an extended study hold to be applied while further data and information are sought. The DSMB will make its recommendation to the Sponsor, who will have ultimate responsibility for acting on the recommendation.

SAEs that are suspected to be related to the vaccine will be reported to the Ethics Committee within 15 calendar days of the site becoming aware of the event. If the event is fatal or life-threatening, the event will be reported within 7 calendar days.

Suspected unexpected serious adverse reactions (SUSARs) will be reported according to national regulatory guidelines. The sponsor pledges to inform the Authorities of any trial discontinuation and specify the reason for discontinuation.

The causal relationship between the AE and the product will be evaluated by the investigator. This interpretation will be based on the type of event, the relationship of the event to the time of vaccine administration, and the known biology of vaccine therapy. This will be done according to the following scale:

**0**	**No Relationship**	No temporal relationship to study product ***and***<br>Alternate aetiology (clinical state, environmental or other interventions); ***and***<br>Does not follow known pattern of response to study product
**1**	**Unlikely**	Unlikely temporal relationship to study product ***and***<br>Alternate aetiology likely (clinical state, environmental or other interventions) ***and***<br>Does not follow known typical or plausible pattern of response to study product.
**2**	**Possible**	Reasonable temporal relationship to study product; ***or***<br>Event not readily produced by clinical state, environmental or other interventions; ***or***<br>Similar pattern of response to that seen with other vaccines
**3**	**Probable**	Reasonable temporal relationship to study product; ***and***<br>Event not readily produced by clinical state, environment, or other interventions ***or***<br>Known pattern of response seen with other vaccines
**4**	**Definite**	Reasonable temporal relationship to study product; ***and***<br>Event not readily produced by clinical state, environment, or other interventions;

 © University of Oxford, 2019

		and<br>Known pattern of response seen with other vaccines

Table 12: Guidelines for assessing the relationship of vaccine administration

© University of Oxford, 2019

11. Data Handling and Record Keeping

Data Management

The Principal Investigator will be responsible for receiving, entering, cleaning, querying, analysing and storing all data that accrues from the study. Responsibility for this may be delegated to the data manager at IRSS-URCN. The data will be entered into the subjects' CRFs. Data will be subsequently transferred to an electronic database for analysis.

If any changes to the protocol are necessary during the study a formal amendment will be presented to the sponsor prior to submission to the relevant ethical and regulatory agencies for approval unless to eliminate an immediate hazard(s) to study participant without prior ethics approval. Any unforeseen and unavoidable deviations from the protocol will be documented and filed in as a protocol deviation in the Trial Master File, with explanation.

Data Capture Methods

Data capture will be on paper CRFs. The CRFs will be considered source documents as healthy volunteers will not have hospital case-notes. Alternatively, data capture will be via an offline e-CRF and transferred to the electronic database when at the research centre.

Adverse events will be tabulated in an electronic database (OpenClinica®) for descriptive analysis.

Immunological data will be transferred to an electronic database for analysis without any volunteer identifier apart from the unique volunteer number.

Types of Data

Data collected will include solicited and non-solicited adverse event data, concomitant medications, clinical laboratory and exploratory immunology data. Source documents will include laboratory results and the case record file containing the case report forms for each volunteer as the healthy volunteers participating in this study may not have medical notes.

Timing/Reports

Annual Safety Report: Due on anniversary of Regulatory Approval – sent to Regulatory and Ethical Bodies
Annual Progress Report: Due on anniversary of Ethical Approval – sent to Ethics Committee

Archiving

 © University of Oxford, 2019

The investigator must keep all trial documents until the youngest trial participant reaches age 21. after the completion or discontinuation of the trial.

Protocol Deviations

Any unforeseen and unavoidable deviations from the protocol will be documented and filed in the study file with explanation.

 © University of Oxford, 2019

12. Data Access and Quality Assurance

Direct Access to Source Data/Documents

The principal investigator will provide direct access to the source data documents to the Ethics Committee, to the regulatory agency, and to authorised representatives of the sponsor, permitting trial-related monitoring and audits.

Quality Assurance

Modifications to the Protocol

Any amendments to the trial that appear necessary during the course of the trial must be discussed by the investigator and sponsor concurrently unless to eliminate an immediate hazard(s) to study participants. If agreement is reached concerning the need for a substantial amendment, it will be produced in writing by the sponsor and/or the investigator and will be made a formal part of the protocol. Any substantial amendment requires Ethics Committee approval, but non-substantial amendments do not.

All substantial amendments must also be communicated to Regulatory Authorities, if appropriate.
An administrative or non-substantial change to the protocol is one that modifies administrative and logistical aspects of a protocol but does not affect the subjects' safety, the objectives of the trial and its progress. An administrative change does not require Ethics Committee approval. However, the Ethics committee must be notified whenever an administrative or non-substantial change is made.
The investigator is responsible for ensuring that substantial amendments to an approved trial, during the period for which Ethics Committee approval has already been given, are not initiated without Ethics Committee review and approval except to eliminate apparent immediate hazards to the subject.

Monitoring

Initiation Visit

An initiation visit will be performed before the inclusion of the first subject in the study. The Monitor will verify and document that the material to be used during the trial has been received and that the investigational team has been properly informed about the trial and regulatory requirements.

 © University of Oxford, 2019

Follow-Up Visits

The Monitor will carry out regular follow-up visits. The investigator commits to being available for these visits and to allow the monitoring staff direct access to subject medical files, if existing, and CRFs. The Monitor is committed to professional secrecy.
During the visits, the Monitor may:

- Carry out a quality control of trial progress: in respect of protocol and operating guidelines, data collection, signature of consent forms, completion of documents, SAE, sample and product management, cold chain monitoring
- Inspect the CRFs, TMF and correspondent correction sheets

The Monitor will discuss any problem with the investigator and define with him the actions to be taken.

Close-out Visit

A close-out visit will be performed at the end of the trial. Its goals are to make sure that:

- The centre has all the documents necessary for archiving
- All unused material has been recovered
- All vaccines have been accounted for

 © University of Oxford, 2019

13. ETHICAL CONSIDERATIONS

Ethical Review

Before the inclusion of the first participant in the study, the protocol must be approved by Ethical Review Committees in Burkina Faso and Oxford (OXTREC).

Informed Consent

Although consent from one parent is sufficient, mothers of potential participants will be encouraged to discuss the study with their husbands and to have his agreement before consent is obtained.

The written information is provided in French only and the field workers interpret the written information in a language the carers understand. The field workers involved in the informed consent discussion are trained on the study and the information sheet and consent form, and are trained to discuss the trial in the local languages the carers understand (Moore, Gurunsi, Fulfuldé). The language of the consent process is documented on the consent form. If the carer is not able to read and write in French, an adult witness, impartial of the trial, will be present through the whole consent process and sign and date the consent form.

The child's carer should give written/thumb printed informed consent before the child is included in the trial, after having been informed of the nature of the trial, the potential risks and their obligations. Informed consent forms will be provided in duplicate (original kept by the investigator, one copy kept by the subject's representative).

If a mother is underage, as she is married, she is considered as an emancipated minor and is suitable to give consent for her child. However, in these cases, the father is usually an adult and is asked to give consent unless he is travelling, in which case, the mother would be asked to.

Confidentiality

All blood results and adverse event data will be encoded in an electronic database and stored securely by the principal investigator.

Inducement

© University of Oxford, 2019

There may be a perception amongst carers of children of benefit from physical examination, laboratory screening in the current study, in addition to free health care provided during the study period for non-vaccine related medical problems. We will also offer compensation for transport expenses for all study subjects.

We do not feel these benefits are excessive, and believe it would be unreasonable to request the cooperation of a population in regular employment or with childcare responsibilities without offering compensation for time.

 © University of Oxford, 2019

14. Indemnity/Compensation/Insurance

Indemnity

Compensation for any injury caused by taking part in this study will be in accordance with the guidelines of the Association of the British Pharmaceutical Industry (ABPI). Broadly speaking the ABPI guidelines recommend that 'the sponsor', without legal commitment, should compensate participants without them having to prove that it is at fault. This applies in cases where it is likely that such injury results from giving any new drug or any other procedure carried out in accordance with the protocol for the study. 'The sponsor' will not compensate participants where such injury results from any procedure carried out which is not in accordance with the protocol for the study. Participants' right at law to claim compensation for injury where negligence can be proven is not affected. In this instance the University of Oxford is the Research Sponsor Institution.

Compensation

Carers of children enrolled in the study will be offered compensation for transport expenses.

Insurance

Investigators participating in this trial will receive insurance coverage from the University clinical trials insurance policy.

 © University of Oxford, 2019

15. References

1. WHO. World Malaria Report 2018. Geneva: World Health Organisation. 2018 November 2018.
2. Gething PW, Casey DC, Weiss DJ, Bisanzio D, Bhatt S, Cameron E, et al. Mapping Plasmodium falciparum Mortality in Africa between 1990 and 2015. The New England journal of medicine. 2016;375(25):2435-45.
3. Dondorp AM, Nosten F, Yi P, Das D, Phyo AP, Tarning J, et al. Artemisinin resistance in Plasmodium falciparum malaria. The New England journal of medicine. 2009;361(5):455-67.
4. Ashley EA, Dhorda M, Fairhurst RM, Amaratunga C, Lim P, Suon S, et al. Spread of artemisinin resistance in Plasmodium falciparum malaria. The New England journal of medicine. 2014;371(5):411-23.
5. Sutherland CJ, Lansdell P, Sanders M, Muwanguzi J, van Schalkwyk DA, Kaur H, et al. pfk13-Independent Treatment Failure in Four Imported Cases of Plasmodium falciparum Malaria Treated with Artemether-Lumefantrine in the United Kingdom. Antimicrobial agents and chemotherapy. 2017;61(3).
6. Maxmen A. Malaria surge feared. Nature. 2012;485(7398):293.
7. Group MVF. Malaria VaccineTechnology Roadmap. 2013.
8. Efficacy and safety of RTS,S/AS01 malaria vaccine with or without a booster dose in infants and children in Africa: final results of a phase 3, individually randomised, controlled trial. Lancet (London, England). 2015;386(9988):31-45.
9. Machingaidze S, Wiysonge CS, Hussey GD. Strengthening the expanded programme on immunization in Africa: looking beyond 2015. PLoS medicine. 2013;10(3):e1001405.
10. Mohr E, Siegrist CA. Vaccination in early life: standing up to the challenges. Current opinion in immunology. 2016;41:1-8.
11. Siegrist CA. Neonatal and early life vaccinology. Vaccine. 2001;19(25-26):3331-46.
12. Klein SL, Shann F, Moss WJ, Benn CS, Aaby P. RTS,S Malaria Vaccine and Increased Mortality in Girls. MBio. 2016;7(2):e00514-16.
13. Coppi A, Natarajan R, Pradel G, Bennett BL, James ER, Roggero MA, et al. The malaria circumsporozoite protein has two functional domains, each with distinct roles as sporozoites journey from mosquito to mammalian host. The Journal of experimental medicine. 2011;208(2):341-56.
14. Bejon P, Andrews L, Andersen RF, Dunachie S, Webster D, Walther M, et al. Calculation of liver-to-blood inocula, parasite growth rates, and preerythrocytic vaccine efficacy, from serial quantitative polymerase chain reaction studies of volunteers challenged with malaria sporozoites. The Journal of infectious diseases. 2005;191(4):619-26.
15. Kappe SH, Buscaglia CA, Nussenzweig V. Plasmodium sporozoite molecular cell biology. Annual review of cell and developmental biology. 2004;20:29-59.
16. Cerami C, Frevert U, Sinnis P, Takacs B, Clavijo P, Santos MJ, et al. The basolateral domain of the hepatocyte plasma membrane bears receptors for the circumsporozoite protein of Plasmodium falciparum sporozoites. Cell. 1992;70(6):1021-33.
17. Hollingdale MR, Nardin EH, Tharavanij S, Schwartz AL, Nussenzweig RS. Inhibition of entry of Plasmodium falciparum and P. vivax sporozoites into cultured cells; an in vitro assay of protective antibodies. Journal of immunology (Baltimore, Md : 1950). 1984;132(2):909-13.

 © University of Oxford, 2019

18. Zavala F, Chai S. Protective anti-sporozoite antibodies induced by a chemically defined synthetic vaccine. Immunology letters. 1990;25(1-3):271-4.
19. Wang R, Charoenvit Y, Corradin G, Porrozzi R, Hunter RL, Glenn G, et al. Induction of protective polyclonal antibodies by immunization with a Plasmodium yoelii circumsporozoite protein multiple antigen peptide vaccine. Journal of immunology (Baltimore, Md : 1950). 1995;154(6):2784-93.
20. Romero P, Maryanski JL, Corradin G, Nussenzweig RS, Nussenzweig V, Zavala F. Cloned cytotoxic T cells recognize an epitope in the circumsporozoite protein and protect against malaria. Nature. 1989;341(6240):323-6.
21. Tsuji M, Romero P, Nussenzweig RS, Zavala F. CD4+ cytolytic T cell clone confers protection against murine malaria. The Journal of experimental medicine. 1990;172(5):1353-7.
22. Backhed F, Roswall J, Peng Y, Feng Q, Jia H, Kovatcheva-Datchary P, et al. Dynamics and Stabilization of the Human Gut Microbiome during the First Year of Life. Cell host & microbe. 2015;17(5):690-703.
23. Madan JC, Hoen AG, Lundgren SN, Farzan SF, Cottingham KL, Morrison HG, et al. Association of Cesarean Delivery and Formula Supplementation With the Intestinal Microbiome of 6-Week-Old Infants. JAMA pediatrics. 2016;170(3):212-9.
24. Fouhy F, Ross RP, Fitzgerald GF, Stanton C, Cotter PD. Composition of the early intestinal microbiota: knowledge, knowledge gaps and the use of high-throughput sequencing to address these gaps. Gut microbes. 2012;3(3):203-20.
25. Vangay P, Ward T, Gerber JS, Knights D. Antibiotics, pediatric dysbiosis, and disease. Cell host & microbe. 2015;17(5):553-64.
26. Doan T, Arzika AM, Ray KJ, Cotter SY, Kim J, Maliki R, et al. Gut Microbial Diversity in Antibiotic-Naive Children After Systemic Antibiotic Exposure: A Randomized Controlled Trial. Clinical infectious diseases : an official publication of the Infectious Diseases Society of America. 2017;64(9):1147-53.
27. Azad MB, Konya T, Persaud RR, Guttman DS, Chari RS, Field CJ, et al. Impact of maternal intrapartum antibiotics, method of birth and breastfeeding on gut microbiota during the first year of life: a prospective cohort study. BJOG : an international journal of obstetrics and gynaecology. 2016;123(6):983-93.
28. Gomez de Aguero M, Ganal-Vonarburg SC, Fuhrer T, Rupp S, Uchimura Y, Li H, et al. The maternal microbiota drives early postnatal innate immune development. Science. 2016;351(6279):1296-302.
29. Honda K, Littman DR. The microbiota in adaptive immune homeostasis and disease. Nature. 2016;535(7610):75-84.
30. Huda MN, Lewis Z, Kalanetra KM, Rashid M, Ahmad SM, Raqib R, et al. Stool microbiota and vaccine responses of infants. Pediatrics. 2014;134(2):e362-72.
31. Harris VC, Armah G, Fuentes S, Korpela KE, Parashar U, Victor JC, et al. Significant Correlation Between the Infant Gut Microbiome and Rotavirus Vaccine Response in Rural Ghana. J Infect Dis. 2017;215(1):34-41.
32. Olotu A, Lusingu J, Leach A, Lievens M, Vekemans J, Msham S, et al. Efficacy of RTS,S/AS01E malaria vaccine and exploratory analysis on anti-circumsporozoite antibody titres and protection in children aged 5-17 months in Kenya and Tanzania: a randomised controlled trial. The Lancet infectious diseases. 2011;11(2):102-9.
33. Agnandji ST, Lell B, Soulanoudjingar SS, Fernandes JF, Abossolo BP, Conzelmann C, et al. First results of phase 3 trial of RTS,S/AS01 malaria vaccine in African children. The New England journal of medicine. 2011;365(20):1863-75.

 © University of Oxford, 2019

34. Magnusson SE, Reimer JM, Karlsson KH, Lilja L, Bengtsson KL, Stertman L. Immune enhancing properties of the novel Matrix-M adjuvant leads to potentiated immune responses to an influenza vaccine in mice. Vaccine. 2013;31(13):1725-33.
35. Reimer JM, Karlsson KH, Lovgren-Bengtsson K, Magnusson SE, Fuentes A, Stertman L. Matrix-M adjuvant induces local recruitment, activation and maturation of central immune cells in absence of antigen. PloS one. 2012;7(7):e41451.
36. Radosevic K, Rodriguez A, Mintardjo R, Tax D, Bengtsson KL, Thompson C, et al. Antibody and T-cell responses to a virosomal adjuvanted H9N2 avian influenza vaccine: impact of distinct additional adjuvants. Vaccine. 2008;26(29-30):3640-6.
37. Madhun AS, Haaheim LR, Nilsen MV, Cox RJ. Intramuscular Matrix-M-adjuvanted virosomal H5N1 vaccine induces high frequencies of multifunctional Th1 CD4+ cells and strong antibody responses in mice. Vaccine. 2009;27(52):7367-76.
38. Collins K, Cottingham, M.G., Gilbert, S.C. & Hill, A. . R21, a new particulate immunogen based on the P. falciparum circumsporozoite protein. Unpublished data 2011.
39. Hutchings CL, Birkett AJ, Moore AC, Hill AV. Combination of protein and viral vaccines induces potent cellular and humoral immune responses and enhanced protection from murine malaria challenge. Infection and immunity. 2007;75(12):5819-26.
40. Cox RJ, Pedersen G, Madhun AS, Svindland S, Saevik M, Breakwell L, et al. Evaluation of a virosomal H5N1 vaccine formulated with Matrix M adjuvant in a phase I clinical trial. Vaccine. 2011;29(45):8049-59.
41. Collins KA, Snaith R, Cottingham MG, Gilbert SC, Hill AVS. Enhancing protective immunity to malaria with a highly immunogenic virus-like particle vaccine. Scientific reports. 2017;7:46621.
42. Plotkin SA, Wiktor T. Rabies vaccination. Annual review of medicine. 1978;29:583-91.
43. Cox JH, Schneider LG. Prophylactic immunization of humans against rabies by intradermal inoculation of human diploid cell culture vaccine. Journal of clinical microbiology. 1976;3(2):96-101.
44. WHO. Burkina Faso: Surveillance Hebdomadaire Du Paludisme A La Semaine. 2018.

Primary Care
Clinical Trials Unit

STATISTICAL ANALYSIS PLAN

VAC 076

Version: 2.0

Date: 27th January 2020

	NAME	TITLE	SIGNATURE	DATE
Written by:	Nicola Williams	Senior Trial Statistician		
Reviewed by:	Katie Ewer<br><br>Mehreen Datoo	Co-investigators		12th Nov 2020
Approved by:	Adrian Hill	Chief Investigator		12th Nov 2020

<u>Version History</u>

Version:	Version Date:	Changes:
0.1	5.6.19	First version
0.2	13.6.19	Changes made following comments on version 1 from Katie Ewer and Mehreen Datoo<br><br>Addition of mixed effects model for immunogenicity outcomes measured at multiple time points<br><br>Additional interim analyses provided by Rachel Roberts in email dated 7.6.19

		Updated consort diagram to 3 arms<br><br>Addition of safety subgroup analyses by gender
0.3	25.6.19	Changes made following meeting with KE, RR and MD: Updates to population to be analysed (modified ITT); addition of subgroup analyses for age and sex
0.4	13.8.19	Changes made following comments from KE, RR, MD:<br><br>Modified ITT primary analysis and per protocol secondary/sensitivity.<br><br>Interim analysis section removed<br><br>Additions made to changes from protocol section
0.5	4.9.19	Changes made following comments from RR and MD – additional safety analysis regarding meningitis
0.6	11.9.19	Changes made following comments from RR and MD<br><br>Addition of number of episodes of malaria to outcomes list and analysis<br><br>Bednet use adjustment clarified<br><br>Age categories for adjustment clarified
0.7	7.11.19	Changed efficacy analysis from 14 days after the last vaccination rather than the day of final vaccination throughout at request of Adrian Hill<br><br>Specified the 31 December secondary endpoint at request of Adrian Hill

1.0	26.11.19	Approved version converted to version 1.0
1.1	26.01.20	Specification that the time to event analysis (by Cox regression), specified as the primary endpoint in the protocol, should be undertaken for the analysis to 31 December 2019 in addition to the proportions (of subjects with a first episode) analysis.
1.2	22.4.20	SAP updated to reflect new version of Protocol v4.0 9th September 2019 (adding booster dose of vaccine)
2.0	12th Nov 2020	Version 1.2 cleaned and approved

TABLE OF CONTENTS

TABLE OF CONTENTS **4**

INTRODUCTION **5**

1.1 PREFACE 5
1.2 PURPOSE AND SCOPE OF THE PLAN 5
1.3 TRIAL OVERVIEW 5
1.4 OBJECTIVES 5
1.4.1 Primary Objective *5*
1.4.2 Secondary Objectives *6*
1.4.3 Exploratory Objectives *6*

2 TRIAL DESIGN **7**

2.1 OUTCOMES MEASURES 7
2.1.1 Primary outcome *7*
2.1.2 Secondary outcomes *7*
2.2 TARGET POPULATION 9
2.3 SAMPLE SIZE 10
2.4 RANDOMISATION AND BLINDING IN THE ANALYSIS STAGE 10

3 ANALYSIS – GENERAL CONSIDERATIONS **11**

3.1 CHARACTERISTICS OF PARTICIPANTS 11
3.2 DEFINITION OF POPULATION FOR ANALYSIS 11
3.2.1 Efficacy Outcomes *11*
3.2.2 Immunology Outcomes *12*
3.2.3 Safety Outcomes *12*
3.2.4 Groups for comparison and time points for analysis *12*
3.3 DATA MONITORING COMMITTEE AND INTERIM ANALYSES 12
3.3.1 Data Safety Monitoring Board *12*

4 PRIMARY ANALYSIS **12**

4.1 PRIMARY OUTCOME 12
4.2 HANDLING MISSING DATA 13
4.3 MULTIPLE COMPARISONS AND MULTIPLICITY 13
4.4 MODEL ASSUMPTIONS 13

5 SECONDARY ANALYSIS **13**

5.1 PRIMARY OUTCOME 13
5.2 SECONDARY OUTCOMES 13

6 SENSITIVITY ANALYSIS **14**

7 SUBGROUP ANALYSES **14**

8 SAFETY ANALYSIS **14**

9 VALIDATION **15**

10 CHANGES TO THE PROTOCOL OR PREVIOUS VERSIONS OF SAP **15**

11 APPENDICES **16**

INTRODUCTION

1.1 PREFACE

Study Physician: Dr Athanase M.Some

Principal Investigator: Dr Halidou Tinto

Project Manager: Ms Rachel Roberts

This SAP supports version 4.0 of the protocol dated 9th September 2019.

1.2 PURPOSE AND SCOPE OF THE PLAN

This document details the proposed analysis of the main paper(s) reporting results from the EDCTP funded phase Ib/IIb trial exploring the safety, immunogenicity and efficacy of a candidate malaria vaccine, R21 adjuvanated with Matrix-M, in 5-17 month old children in Nanoro, Burkina Faso. The results reported in these papers should follow the strategy set out here. Subsequent analyses of a more exploratory nature will not be bound by this strategy, though they are expected to follow the broad principles laid down here. The principles are not intended to curtail exploratory analysis (for example, to decide cut-points for categorisation of continuous variables), nor to prohibit accepted practices (for example, data transformation prior to analysis), but they are intended to establish the rules that will be followed, as closely as possible, when analysing and reporting the trial.

The analysis strategy will be available on request when the principal papers are submitted for publication in a journal. Suggestions for subsequent analyses by journal editors or referees, will be considered carefully, and carried out as far as possible in line with the principles of this analysis strategy; if reported, the source of the suggestion will be acknowledged.

Any deviations from the statistical analysis plan will be described and justified in the final report of the trial. The analysis should be carried out by an identified, appropriately qualified and experienced statistician, who should ensure the integrity of the data during their processing. Examples of such procedures include quality control and evaluation procedures.

1.3 TRIAL OVERVIEW

This is a Phase Ib/IIb randomised controlled trial of the safety, immunogenicity and efficacy of a candidate malaria vaccine, R21 adjuvanted with Matrix-M (R21/MM), in 5-17 month old children in Nanoro, Burkina Faso.

1.4 OBJECTIVES

1.4.1 PRIMARY OBJECTIVE

To assess the protective efficacy against clinical malaria of R21 adjuvanted with Matrix-M in 5-17 months old children living in a malaria-endemic area, for 6 months from 14 days after the third vaccination.

1.4.2 Secondary Objectives

- Duration of Protective efficacy against clinical malaria

To assess the protective efficacy against clinical malaria of R21 adjuvanted with Matrix-M in 5-17 months old children living in a malaria-endemic area, for 12 months from 14 days after administration of the third dose of vaccine.

To assess the protective efficacy against clinical malaria of R21 adjuvanted with Matrix-M in 5-17 months old children living in a malaria-endemic area, for 6 months after a booster vaccination.

To assess the protective efficacy against clinical malaria of R21 adjuvanted with Matrix-M in 5-17 months old children living in a malaria-endemic area, for 12 months after a booster vaccination.

- Efficacy against asymptomatic P. falciparum infection

To assess the protective efficacy against asymptomatic P. falciparum infection of R21 adjuvanted with Matrix-M in 5-17 months old children living in a malaria-endemic area, at 12 months from 14 days after administration of the third dose of vaccine.

To assess the protective efficacy against asymptomatic *P. falciparum* infection of R21 adjuvanted with Matrix-M in 5-17 months old children living in a malaria-endemic area, at 12 months after administration of the booster dose of vaccine.

- Safety Objectives

To assess the safety and reactogenicity of R21 adjuvanted with Matrix-M in 5-17 months old children living in a malaria-endemic area, in the month following each vaccination and at 12 months after administration of the third dose of vaccine.

To assess the safety and reactogenicity of R21 adjuvanted with Matrix-M in 5-17 months old children living in a malaria-endemic area, in the month following each vaccination and at 12 months after administration of the booster dose of vaccine.

- Immunogenicity Objectives

To assess the humoral immunogenicity of R21 adjuvanted with Matrix-M in 5-17 months old children living in a malaria-endemic area.

1.4.3 Exploratory Objectives

Efficacy against incident cases of severe malaria

1. To assess the protective efficacy against severe malaria of R21 adjuvanted with Matrix-M in 5-17 months old infants living in a malaria-endemic area, at 6 months from 14 days after administration of the third dose of vaccine.
2. To assess the protective efficacy against severe malaria of R21 adjuvanted with Matrix-M in 5-17 months old infants living in a malaria-endemic area, at 6 months after administration of the booster dose of vaccine
3. To evaluate cellular immunogenicity and other exploratory immunological end points.

2 TRIAL DESIGN

This is a double blind randomised controlled trial with 3 arms. Participants will be randomised 1:1:1 to receive vaccination with the IMP (R21 adjuvanted with Matrix-M; Groups 1&2) or control vaccination with Rabies Vaccine (Group 3). Participants and investigators will be blinded to group allocation for each participant. Efficacy of vaccination will be assessed by comparing the development of malaria between Groups 1 & 2 versus Group 3 participants.

There are two study vaccines: the IMP, R21 adjuvanted with Matrix-M; and Rabies Vaccine. Group 1 (active vaccine group) participants will receive 3 vaccinations of R21 5µg with 25µg Matrix-M and Group 2 will receive 3 vaccinations of R21 5µg with 50µg Matrix-M , 4 weeks apart via the intramuscular route. The same thigh will be used for vaccinations. Group 3 (control group) participants will receive three vaccinations with rabies vaccine, four weeks apart, all given intramuscularly. The same thigh will be used for these vaccinations.

All groups will receive a fourth booster vaccination before the malaria season commences the following year.

Week	0	4	8	Boost
**Group 1**<br><br>**n=150**	5µg R21/25µg Matrix-M	5µg R21/25µg Matrix-M	5µg R21/25µg Matrix-M	5µg R21/25µg Matrix-M
**Group 2**<br><br>**n=150**	5µg R21/50µg Matrix-M	5µg R21/50µg Matrix-M	5µg R21/50µg Matrix-M	5µg R21/50µg Matrix-M
**Group 3**<br><br>**n=150**	(Control vaccine)	(Control vaccine)	(Control vaccine)	(Control vaccine)

Planned study procedures and timings are shown in Appendix I.

2.1 OUTCOMES MEASURES

2.1.1 PRIMARY OUTCOME

The primary outcome is time to first episode of malaria, meeting the primary case definition of clinical malaria episode, over a period of 6 months of follow up from 14 days after the third vaccination.

2.1.2 SECONDARY OUTCOMES

2.1.2.1 EFFICACY OUTCOMES

- Time to first episode of malaria meeting the primary case definition of clinical malaria episode, over a period of 12 months of follow up from 14 days after third vaccination.
- Time to first episode of malaria meeting the primary case definition of clinical malaria episode over the period from 14 days after third vaccination to 31 December 2019.

- Time to first episode of malaria meeting the primary case definition of clinical malaria episode, over a period of 6 months of follow up from 14 days after third vaccination.
- Time to first episode of malaria meeting the primary case definition of clinical malaria episode, over a period of 6 months of follow up from 14 days after booster vaccination.
- Time to first episode of malaria meeting the primary case definition of clinical malaria episode, over a period of 12 months of follow up from 14 days after booster vaccination.
- Proportion of participants with an episode of malaria meeting the primary case definition of clinical malaria episode, over a period of 6 months of follow up from 14 days after third vaccination.
- Proportion of participants with an episode of malaria meeting the primary case definition of clinical malaria episode, over the period from 14 days post third vaccination to 31 December 2019.
- Proportion of participants with an episode of malaria meeting the primary case definition of clinical malaria episode, over a period of 12 months of follow up from 14 days after the third vaccination.
- Proportion of participants with an episode of malaria meeting the primary case definition of clinical malaria episode, between day 14 post 2nd vaccination and 14 days after the 3rd vaccination.
- Proportion of participants with an episode of malaria meeting the primary case definition of clinical malaria episode, over a period of 6 months of follow up from 14 days after booster vaccination.
- Proportion of participants with an episode of malaria meeting the primary case definition of clinical malaria episode, over a period of 12 months of follow up from 14 days after booster vaccination.
- Time to first episode of malaria meeting the primary case definition of clinical malaria episode, over the period from 14 days after the 2nd vaccination to 14 days after the 3rd vaccination.
- Proportion of participants with an episode of malaria meeting the primary case definition of clinical malaria episode, between 28 days after 3rd vaccination and 6 months post 3rd vaccination.
- Number of episodes of malaria meeting the primary case definition of clinical malaria episode, over a period of 12 months of follow up from 14 days after third vaccination
- Proportion of participants meeting the primary case definition of asymptomatic P. falciparum infection, at study days 236 and 421 (6 and 12 months post third vaccination).
- Proportion of participants meeting the primary case definition of asymptomatic P. falciparum infection 12 months after 14 days post booster vaccination
- Time to first episode of malaria meeting the secondary case definition of clinical malaria episode, over a period of 6 months of follow up from 14 days after third vaccination.
- Time to first episode of malaria meeting the secondary case definition of clinical malaria episode, over a period of 12 months of follow up from 14 days after third vaccination.
- Time to first episode of malaria meeting the secondary case definition of clinical malaria episode over the period from 14 days after third vaccination to 31 December 2019.
- Proportion of participants with an episode of malaria meeting the secondary case definition of clinical malaria episode, over the period from 14 days after the third vaccination to 31 December 2020.
- Proportion of participants with an episode of malaria meeting the secondary case definition of clinical malaria episode, over a period of 6 months of follow up from 14 days after third vaccination.
- Proportion of participants with an episode of malaria meeting the secondary case definition of clinical malaria episode, over a period of 12 months of follow up from 14 days after third vaccination.
- Number of cases of clinical malaria, as in the primary case definition, averted per 1000 children vaccinated at 6 and 12 months post 3rd vaccination (calculated as the number of cases in the control group minus the number of cases in the vaccine group, expressed per 1000 participants vaccinated (as reported in journals.plos.org/plosmedicine/article?id=10.1371/journal.pmed.1001685)).

2.1.2.2 IMMUNOGENICITY OUTCOMES

- Total IgG (ELISA units) antibodies against the NANP repeat region of CSP and antiHBs.
- Avidity of IgG antibodies against the NANP repeat region of CSP
- Frequency of memory B and plasma cells (measured by flow cytometry)
- Frequency of T follicular helper cell subsets
- The magnitude of these humoral and cellular immune responses will be evaluated for correlation with malaria episodes

2.1.2.3 SAFETY OUTCOMES

- Any SAEs occurring from first vaccination until the end of the study.
- Local and systemic solicited adverse events, occurring from first vaccination until 7 days post vaccination. These adverse events are collected following each vaccination for 7 days.
- All unsolicited adverse events, occurring from first vaccination until 28 days post third vaccination (study day 84). These will also be broken down into the following categories: 1) occurring between day of 1st vaccination and day of 2nd vaccination; 2) occurring between day of 2nd vaccination and day of 3rd vaccination; ; 3) occurring between 3rd vaccination and 28 days post 3rd vaccination.
- Local and systemic solicited and unsolicited adverse events,occurring from booster vaccination until 28 days post boost vaccination.
- Safety laboratory measures (haematological parameters measured by full blood count and biochemistry – creatinine, ALT, AST, glucose and bilirubin).

2.1.2.4 EXPLORATORY OUTCOMES

- Time to first episode of severe malaria, meeting the primary case definition of severe malaria over a period of 6 months of follow up from 14 days after third vaccination
- Proportion of participants with an episode of severe malaria meeting the primary case definition of severe malaria, over a period of 6 months of follow up from 14 days after third vaccination.
- Time to first episode of severe malaria, meeting the secondary case definition of severe malaria over a period of 6 months of follow up from 14 days after third vaccination
- Proportion of participants with an episode of severe malaria meeting the secondary case definition of severe malaria, over a period of 6 months of follow up from 14 days after third vaccination.
- Time to first episode of severe malaria, meeting the primary case definition of severe malaria over a period of 6 months of follow up from 14 days after booster vaccination
- Proportion of participants with an episode of severe malaria meeting the primary case definition of severe malaria, over a period of 6 months of follow up from 14 days after booster vaccination.
- Time to first episode of severe malaria, meeting the secondary case definition of severe malaria over a period of 6 months of follow up from 14 days after booster vaccination
- Proportion of participants with an episode of severe malaria meeting the secondary case definition of severe malaria, over a period of 6 months of follow up from 14 days after booster vaccination

2.2 TARGET POPULATION

The inclusion criteria will be used at screening to identify participants eligible for the study, and will be checked prior to vaccination to confirm ongoing eligibility. Eligible infants will fulfil all of the inclusion criteria and none of the exclusion criteria.

Inclusion Criteria

- Healthy child aged 5-17 months at the time of first study vaccination
- Provide written Informed consent of parent/guardian
- Child and parent/guardian resident in the study area villages and anticipated to be available for vaccination and follow-up for 2 years following last dose of vaccination

Exclusion Criteria

- Clinically significant skin disorder (psoriasis, contact dermatitis etc.), immunodeficiency, cardiovascular disease, respiratory disease, endocrine disorder, liver disease, renal disease, gastrointestinal disease, neurological illness.
- Weight-for-age *Z* score of less than –3 or other clinical signs of malnutrition.
- History of allergic reaction, significant IgE-mediated event, or anaphylaxis to immunisation.
- History of allergic disease or reactions likely to be exacerbated by any component of the vaccines, e.g. egg products, neomycin.
- Clinically significant laboratory abnormality as judged by the study clinician.
- Blood transfusion within one month of enrolment.
- Administration of immunoglobulins and/or any blood products within the three months preceding the planned administration of the vaccine candidate.
- Previous vaccination with experimental malaria vaccines.
- Participation in another research study involving receipt of an investigational product in the 30 days preceding enrolment, or planned use during the study period.
- Current participation in another clinical trial, or within 12 weeks of this study.
- Known maternal HIV infection (No testing will be done by the study team).
- Any confirmed or suspected immunosuppressive or immunodeficient state, including HIV infection; asplenia; recurrent, severe infections and chronic (more than 14 days) immunosuppressant medication within the past 6 months (For corticosteroids, this will mean prednisone, or equivalent, ≥ 0.5 mg/kg/day. Inhaled and topical steroids are allowed).
- Any significant disease, disorder or situation which, in the opinion of the Investigator, may either put the participants at risk because of participation in the trial, or may influence the result of the trial, or the participant's ability to participate in the trial.

2.3 Sample size

The primary endpoint is the time to first episode of malaria meeting the primary case definition of clinical malaria episode, over a period of 6 months from 14 days after the third vaccination. The study is powered to provide an initial point estimate of the efficacy of the malaria vaccine, assuming that the vaccine efficacy over 6 months will be greater than 50%. We will aim to time vaccinations so that the final vaccination happens preferably near the beginning of the malaria season, which runs roughly from June to November.

2.4 Randomisation and blinding in the analysis stage

Double-blinding will be used to reduce bias in evaluating the study endpoints. Double-blinding in this context means that the vaccine recipient, their parent(s)/guardian(s), all investigators and the study team responsible for the evaluation of efficacy, safety and immunogenicity endpoints will all be unaware of the exact treatment, (IMP or rabies vaccine) given to the participant. The only study staff aware of the vaccine assignment for IMP or rabies vaccine will be those responsible for the storage and preparation of vaccines; these staff will play no other role in the study. The vaccines will be different in terms of volume and colour. Therefore, the contents of the

syringe will be masked with an opaque label to ensure that parent(s)/guardian(s), as well as nurse administering the vaccine are blinded.

Consenting participants who have satisfied all the eligibility criteria and completed the baseline assessment will be individually randomised to one of three study groups using a pre-printed envelope system. Participants will not be randomised until after consent has been taken and baseline assessments have been completed. Randomisation will use a 1:1:1 allocation when all participants have been recruited.

Allocation will be carried out using using sequentially numbered opaque sealed envelopes. An independent statistician will generate a random allocation list using block randomisation with variable block sizes. A person independent of the trial will prepare and seal the envelopes using this list, and then provide to the investigator. The independent statistician will not be part of the study team.

The study pharmacists will only be allowed to access to open an envelope after ensuring that the child before them has met all eligibility criteria and has been given a study ID number. For each child, eligibility will have to be counter checked and signed by a second person before allocation of study ID number. All envelopes will be retained to be checked by the clinical monitor.

The local safety monitor, who is independent from the study team, will also be provided with the allocations of Groups 1,2 &3. If deemed necessary for reasons such as safety, the Local Safety monitor will unblind the specific enrolled subject without revealing the study group to the investigators.

3 Analysis – General considerations

3.1 Characteristics of participants

Summary statistics of baseline demographic and clinical variables by allocation group will be assessed to ensure balance of these characteristics between the three randomised groups.

Frequencies and percentages will be reported for categorical variables, means and standard deviation will be reported for continuous variables if normally distributed, median and interquartile range if skewed. Number with missing data for each characteristic will be presented. No formal statistical testing will be applied to test for any difference between randomised groups with respect to the baseline characteristics and no confidence intervals will be presented.

Patient flow from screening through randomisation, follow up and analysis will be presented in a CONSORT flow chart (Appendix II).

3.2 Definition of population for analysis

3.2.1 Efficacy Outcomes

The primary analysis will be based on a modified intention-to-treat population. That is, all participants will be analysed in the groups to which they were randomised, regardless of which vaccine they received. They will remain in the analysis regardless of how many trial visits they have attended, but only if they have received all 3 vaccinations.

An unadjusted analysis of the primary outcome will also be carried out on the per-protocol population. This will include all participants who are eligible to participate, and received all allocated vaccinations within the specified time window periods of 4 week intervals plus or minus 3 days, without any contraindications to vaccine administration.

3.2.2 Immunology Outcomes

Participants will be included in the analysis of immunology outcomes if they have received a minimum of 3 vaccines to which they have been allocated. They do not need to have attended all follow up visits. The immunology outcomes will also be analysed separately for those receiving 2 and 3 vaccines.

3.2.3 Safety Outcomes

The safety analysis will be based on the per protocol population (receiving all 3 doses of allocated vaccine). A secondary safety analysis will include participants who have received at least one of the 3 vaccinations, regardless of how many trial visits they have attended.

3.2.4 Groups for comparison and time points for analysis

The primary groups for comparison will be group 1 vs 3 and group 2 vs 3. If no statistically significant difference is found between groups 1 and 2 then a further analysis will be carried out comparing groups 1 and 2 vs. group 3.

Analysis of the 6 month outcomes will be carried out once the final participant has completed their 6 month post 14 days after dose three assessment. The 12 month outcomes will be analysed once the final participant has completed their 12 month post 14 days post dose three assessment.

3.3 Data Monitoring Committee And Interim Analyses

3.3.1 Data Safety Monitoring Board

The interim safety data to Day 14 of trial participation after the first 30 vaccinations of participants across all groups (1-3) will be presented to the DSMB for review of safety. There will be no planned pause of the trial to allow review but if there are any concerns, the DSMB is able to pause or stop the trial and/or decode the vaccination groups.

Every SAE will be reported to the DSMB within 24 hours and the DSMB may ask for the study to be stopped, or for an extended study hold to be applied while further data and information are sought.

The reports for the DSMB will contain details of vaccinations to date, all solicited AEs collected for 7 days after every vaccination and unsolicited AEs collected for 28 days after vaccination. SAEs will be collected for the duration of the trial. Laboratory AEs (if applicable) will also be detailed in the report as well as any medications the participants may have taken.

4 PRIMARY ANALYSIS

4.1 Primary outcome

The primary outcome is time to first episode of malaria, meeting the primary case definition of clinical malaria episode, over a period of 6 months of follow up from 14 days after last vaccination. The time will be calculated in days as the difference between the date of first episode of malaria and 14 days post the date of last

vaccination. Those without an episode of malaria will be censored at either 6 months post 14 days after last vaccination, or at the date of withdrawal/loss to follow up.

Kaplan Meier curves will be presented. A Cox regression model will be used to test whether time to malaria differs between the randomised groups. The median (interquartile range) for each randomised group will be presented. The model will include randomised groups. Hazard ratios and 95% confidence intervals will be reported to present the difference in time to event between groups 1 and 3 and groups 2 and 3.

Vaccine efficacy will be calculated as 1-HR.

4.2 Handling missing data

The numbers (with percentages) of losses to follow-up (defaulters and withdrawals) will be reported by randomised group at each assessment time point. The number (percentage) of vaccines received will also be presented by randomised group. Missing data will not be imputed in any way.

4.3 Multiple comparisons and multiplicity

The protocol clearly states the primary outcome that is to be compared between the randomised groups. Only one primary outcome has been specified, therefore there are no issues of multiple comparisons and multiplicity. Interpretation of significant secondary analyses will be made with caution.

4.4 Model assumptions

The Cox model assumes that the hazards across the 3 groups are proportional. If this is not the case then alternative survival models will be explored.

5 SECONDARY ANALYSIS

All secondary analyses will be carried out on the modified ITT population.

5.1 Primary outcome

A second analysis will be carried out, following the method detailed above, but adjusting for the confounding factors of gender, age at randomisation (categorised as 5-9 months, 10-12 months and >12 months) and bed net use (days bed net use between randomisation and 14 days post last vaccination).

The analysis detailed above will also be carried out based on the per protocol population described in section 3.2.1, rather than the modified ITT population used in the primary analysis.

5.2 Secondary outcomes

Time to event outcomes will be analysed in the same way as the primary outcome, using both unadjusted and adjusted Cox models.

Binary outcomes will be analysed using a log binomial model, including randomised group as a covariate. Relative risks and 95% confidence intervals will be reported comparing groups 1 and 3 and groups 2 and 3. The analysis will also be carried out adjusting for the confounding factors of gender, age at randomisation (categorised as 5-9 months, 10-12 months and >12 months) and bed net use (days bed net use between randomisation and last vaccination).

Continuous outcomes will be reported as the mean and standard deviation for each group and the difference and 95% CI will be computed using linear regression with and without adjustment for confounding factors. Assumptions of linear regression will be assessed and if violated the data will be log transformed. If the assumptions of linear regression are still not met then a Kruskall Wallis test will be adopted and the median (IQR) will be used to summarise the data and difference in medians (95%CI) will be reported. If the Kruskall Wallis test suggests a difference between the 3 groups then the Mann-Whitney test will be used to compare groups 1 and 3 and groups 2 and 3.

For continuous variables measured at multiple time points (specifically the immunogenicity outcomes) a mixed effect linear regression model will be fitted. The model will utilise data collected at baseline, D28, D84 and D236 (and D421 if the trial is extended), The dependent variable will be the outcome of interest. Participant will be included as a random effect. Fixed effects will include randomised group, baseline value of the outcome of interest, age (categorised as 5-9 months, 10-12 months and >12 months), gender, bednet use (days bed net use between randomisation and 14 days after last vaccination), time and a time by randomised group interaction term to allow estimation of treatment effect at each time point. The difference between groups 1 and 3 and 2 and 3 in mean change at the time points of interest will be reported along with 95% confidence intervals. Durability is determined by the response at D236 (and D421 if the trial is extended).

Number of episodes of malaria will be presented as the number and percentage of children with 0, 1, 2, 3 etc. episodes by randomised group. The groups will be compared using a negative binomial regression analysis and treatment effect reported as incidence rate ratios with 95% confidence intervals. The analysis will also be carried out adjusting for the confounding factors of gender, age at randomisation (categorised as 5-9 months, 10-12 months and >12 months) and bed net use (days bed net use between randomisation and 14 days after last vaccination).

6 SENSITIVITY ANALYSIS

No sensitivity analyses have been specified in the protocol.

7 SUBGROUP ANALYSES

In order to assess whether the vaccine effect differs between males and females, the primary and safety analyses will be run again, including an interaction term between randomised group and gender.

In order to assess whether the vaccine effect differs depending on the age of the child, the primary analysis will be run again, including an interaction term between randomised group and age category. Age will be categorised as 5-9 months, 10-12 months and >12 months.

8 SAFETY ANALYSIS

All solicited and unsolicited local and systemic adverse events (including results of clinical laboratory investigations where deemed adverse events) will be listed. They will be presented according to whether they are possibly, probably or definitely related to vaccination and by vaccination group.

The proportion of patients in each group reporting any local reaction will be compared using the chi-squared test and the difference in proportions with 95% confidence intervals will be presented (comparing groups 1 and 3 and groups 2 and 3). This will be repeated for systemic reactions.

All SAEs will be described in detail for each participant. The proportion of patients in each group reporting at least one SAE will be compared using the chi-squared test and the difference in proportions with 95% confidence intervals will be presented (comparing groups 1 and 3 and groups 2 and 3).

Where a patient reports more than one of the same type of event, separate tables will be presented showing a) counts of events and b) counts of participants experiencing at least one type of this event.

9 VALIDATION

The primary and safety analyses will be validated by a senior trial statistician or an appropriately qualified delegate.

10 CHANGES TO THE PROTOCOL OR PREVIOUS VERSIONS OF SAP

- Change efficacy outcomes to be measured from 14 days after final vaccination rather than from day of final vaccination
- Addition of efficacy outcome: Proportion of participants with an episode of malaria and time to first episode of malaria meeting the primary case definition of clinical malaria episode, over the period from 14 days post final vaccination to 31 December 2019.
- The interim analyses and subgroup analyses outlined in this document were not mentioned in the protocol but were defined prior to end of data collection and before any data had been seen.
- The intention to treat population specified in the protocol has been amended slightly with regards to vaccinations received and visits attended. In the protocol it was specified that the per protocol population would be used for the primary analysis. This has been changed to a secondary analysis, with the modified intention to treat population forming the basis of the primary analysis. This was defined prior to end of data collection and before any data had been seen.
- Secondary efficacy outcomes 4, 5 and 6 and the breakdown of serious adverse events and unsolicited adverse events were not stated in the protocol but have been defined prior to the end of data collection and before data lock.
- Groups for comparison were specified in the protocol as group 1 vs 3, group 2 vs 3 and groups 1 and 2 vs 3 (assuming no difference between groups 1 and 2). This has since been changed to a primary comparison of group 1 vs. 3 and group 2 vs. 3 and, if no difference is found between groups 1 and 2, then a further, secondary analysis will be carried out comparing groups 1 and 2 vs. group 3.
- Number of cases of clinical malaria, as in the primary case definition, averted per 1000 children has been added to the efficacy outcomes.

11 Appendices

Appendix I. Timeline of study visits and procedures for participants in Groups 1, 2 & 3

Study visit number	S	1	2-7	8	9	10-15	16	17	18-23	24	25	26	27	28	29	30	31
**Clinic visit**	X	X		X	X		X	X		X	X					X	X
**Home visit**			X			X			X			X	X	X	X		
**Day of visit**	D-30-D-1	D0	D1-6	D7	D28	D29-34	D35	D56	D57-62	D63	D84	D114	D144	D174	D204	D236	D421
**Window period**				+/- 1	+/- 3		+/- 1	+/- 3		+/- 1	+/- 3	+/- 7	+/- 7	+/- 7	+/- 7	+/- 28	+/- 28
**Vaccination**		X			X			X									
**Inclusion/Exclusion criteria**	X	X			X			X									
**Informed consent**	X																
**Medical history**	X	(X)		(X)	(X)		(X)	(X)		(X)	(X)	(X)	(X)	(X)	(X)	(X)	(X)
**Physical examination**	X	(X)		(X)	(X)		(X)	(X)		(X)	(X)	(X)	(X)	(X)	(X)	(X)	(X)
**Review contraindications to vaccination**		X			X			X									

**Recording of concomitant medication**	X	X		X	X		X	X		X	X	X	X	X	X	X	X
**Recording of solicited AEs**			X	X		X	X		X	X							
**Recording of unsolicited AEs**			X	X	X	X	X	X	X	X	X						
**Recording of SAEs**			X	X	X	X	X	X	X	X	X	X	X	X	X	X	X
**Rectal swab/faecal sample**		X															
**Blood film for Plasmodium species**	X															X	X
**Blood film for Plasmodium species if axillary temp** ≥37.5 and/or history of fever within last 24 hours				X	X		X	X		X	X	X	X	X	X		
**Blood sampling**	X				X						X					X	X

S: Screening Visit; X: procedure takes place, (X): procedure takes place as required at the discretion of the investigators;

D : Day.

Timeline of Study Visits showing blood sampling and laboratory investigations for participants in Groups 1, 2 & 3 receiving a booster (fourth) vaccination prior to the malaria season, the year after the first 3 vaccinations

Day of visit	B-30-B-1	B0	B1-6	B7	B28	B168	B336
Window period				+/- 1	+/- 3	+/- 28	+/- 28
Vaccination		X					
Blood film for Plasmodium species	X					X	X
Blood film for Plasmodium species if axillary temp ≥37.5 and/or history of fever within last 24 hours		X		X	X		
Haematology & Biochemistry	X				X	X	X
Immunology	X				X	X	X

Appendix II. Flow diagram of trial participants

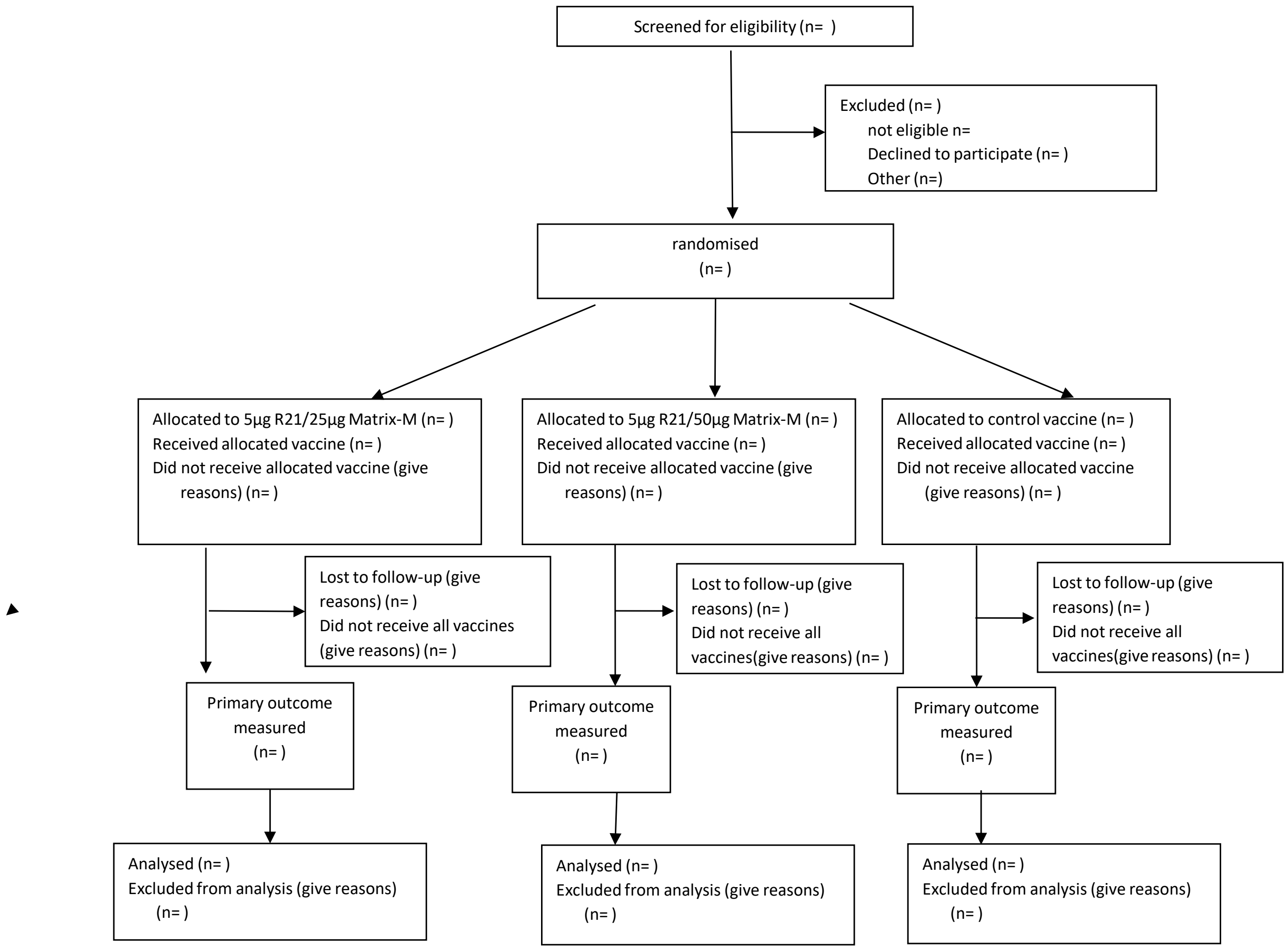

Supplement: Supplementary appendix [file mmc1.pdf]
